# Fluorescence detected circular dichroism (FDCD) for supramolecular host–guest complexes†

**DOI:** 10.1039/d1sc01411k

**Published:** 2021-06-09

**Authors:** Amrutha Prabodh, Yichuan Wang, Stephan Sinn, Paolo Albertini, Christian Spies, Eduard Spuling, Liu-Pan Yang, Wei Jiang, Stefan Bräse, Frank Biedermann

**Affiliations:** Karlsruhe Institute of Technology (KIT), Institute of Nanotechnology (INT) Hermann-von-Helmholtz-Platz 1, 76344 Eggenstein-Leopoldshafen Germany frank.biedermann@kit.edu; Karlsruhe Institute of Technology (KIT), Institute of Organic Chemistry Fritz-Haber-Weg 6 76131 Karlsruhe Germany stefan.braese@kit.edu; JASCO Europe Srl Via Luigi Cadorna 1 23894 Cremella Italy; JASCO Deutschland GmbH Robert-Bosch-Str. 14, 64319 Pfungstadt Germany; Southern University of Science and Technology, Department of Chemistry Xueyuan Boulevard 1088, Nanshan District 518055 Shenzhen China; Karlsruhe Institute of Technology (KIT), Institute of Biological and Chemical Systems – Functional Molecular Systems (ICBS-FMS) Hermann-von-Helmholtz-Platz 1 76344 Eggenstein-Leopoldshafen Germany

## Abstract

Fluorescence-detected circular dichroism (FDCD) spectroscopy is applied for the first time to supramolecular host–guest and host–protein systems and compared to the more known electronic circular dichroism (ECD). We find that FDCD can be an excellent choice for common supramolecular applications, *e.g.* for the detection and chirality sensing of chiral organic analytes, as well as for reaction monitoring. Our comprehensive investigations demonstrate that FDCD can be conducted in favorable circumstances at much lower concentrations than ECD measurements, even in chromophoric and auto-emissive biofluids such as blood serum, overcoming the sensitivity limitation of absorbance-based chiroptical spectroscopy. Besides, the combined use of FDCD and ECD can provide additional valuable information about the system, *e.g.* the chemical identity of an analyte or hidden aggregation phenomena. We believe that simultaneous FDCD- and ECD-based chiroptical characterization of emissive supramolecular systems will be of general benefit for characterizing fluorescent, chiral supramolecular systems due to the higher information content obtained by their combined use.

## Abstract 2

Fluorescence-detected circular dichroism (FDCD) spectroscopy is applied for the first time to supramolecular host–guest and host–protein systems and compared to the more known electronic circular dichroism (ECD).

## Graphical abstract

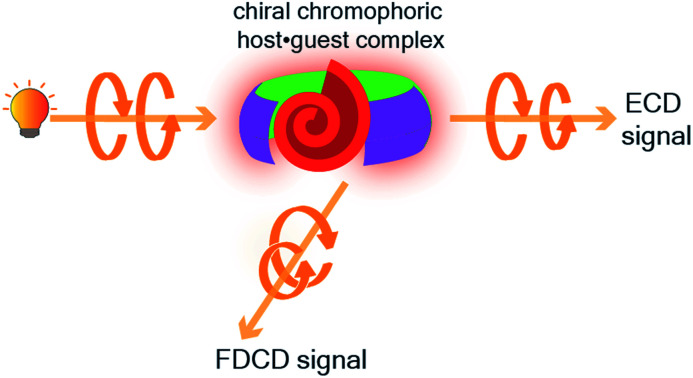

## Introduction

**Fig. 1:**
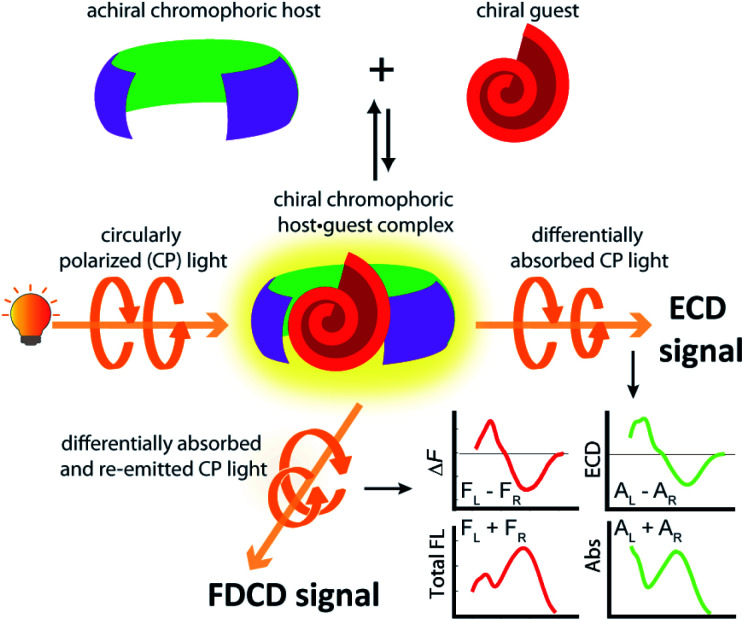
Complexation of a chiral guest by an achiral chromophoric and emissive host can induce ECD and FDCD signal generation. The ECD signal measures the difference in absorption of left-handed and right-handed circularly polarized light by the chiral species whereas FDCD reports on the differential fluorescence intensity that results from excitation with left-handed and right-handed circularly polarized light.

Investigations into the chirality of (bio)chemical systems and monitoring of chiral transformations have provided useful lessons for the design of drugs and functional materials and enriched the general understanding of molecular recognition principles.^1–3^ Electronic circular dichroism (ECD) spectroscopy, which measures the difference in the absorption of left and right circularly polarized light (Fig. 1), has been extensively used for the characterization of chiral, light-absorbing molecules.^4–7^

**Fig. 2:**
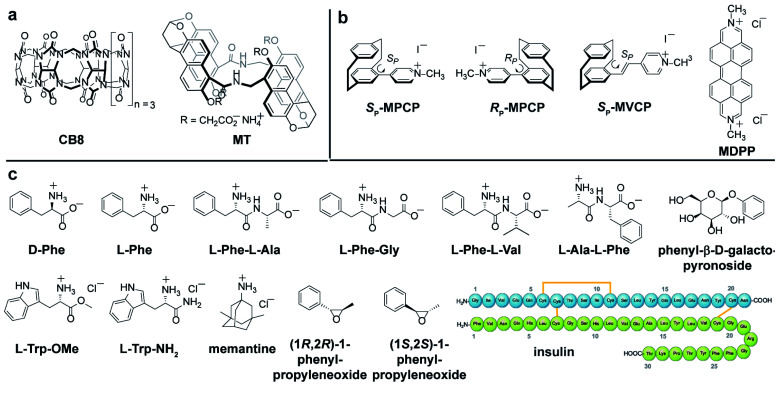
Chemical structure of (a) host, (b) dye molecules and (c) investigated chiral analytes and the achiral drug memantine utilized in this study. Their protonation state represents their occurrence under the measurement conditions. The hosts and the self-assembled CB8·MDPP receptor complex are depicted in bold in the manuscript whereas for the guests the normal font is used.

Most of the (bio)chemical compounds of interest lack a strong chromophoric group and hence, do not produce ECD signals in the practically preferable near UV or visible wavelength region, or are even completely ECD silent. This has prompted the development of chromophoric probes and chemosensors which engage in covalent or non-covalent interactions with the chiral analyte.^8–11^ The complexation of a chiral analyte by an achiral chromophoric “binder” can generally be expected to give rise to chiroptical signals because the chromophore is then situated in a chiral environment.^12–14^ Practically, suitably strong emerging ECD signals are usually obtained if an electronic-coupling between the chromophoric host and the chiral guest occurs, or if the host deforms into a chiral conformation upon analyte binding.^15^ In favourable cases, ECD spectra can contain analyte-specific “induced ECD fingerprints”, which can be utilized for analyte identification and differentiation.^14–16^ Representative supramolecular ECD-based probes and chemosensors were described by Berova,^12,17^ Borhan,^18–20^ Wolf,^16,21–23^ Anslyn,^13,24–26^ Canary,^26–28^ Nau,^14,29,30^ Jiang,^11^ our group^14,15^ and others.^31–33^ Chirality-sensors that operate in aqueous media are particularly attractive because of their application potential in the Life Sciences and for diagnostics.^11,14,34^ For instance, chemosensors that were modularly assembled from the large macrocycle cucurbit[8]uril (CB8, see Fig. 2a) and dicationic reporter dyes in water, respond with induced, analyte-indicative ECD signals to the presence of biorelevant chiral aromatic metabolites such as Trp-containing peptides.^14^

The concentration range where ECD measurements can be applied depends on the intensity of dichroic absorption, and is thus rather narrow.^35,36^ Specifically, the ECD detection limit is set by the molar extinction coefficient of the chromophoric host (or guest), which rarely reaches 10^5^ M^−1^ cm^−1^. Consequently, receptor and analyte concentrations ≥10 μM, oftentimes even >100 μM, have to be used for generating meaningful ECD signals.^11,14,37^ However, many diagnostically relevant analytes only occur in the low micromolar to nanomolar regime in biofluids, and thus escape ECD-based detection protocols. Moreover, most biomolecules and artificial supramolecular systems are prone to aggregate in aqueous environments, which prohibits the use of high concentrations.

The use of fluorescence-based chiroptical methods comes to mind in an effort to improve the sensitivity of chiroptical supramolecular assays. Both fluorescence-detected circular dichroism (FDCD) spectroscopy^38–40^ as well as circularly polarized luminescence (CPL) spectroscopy^41–46^ are worth considering, with the latter receiving a revival in recent years. In essence, CPL measures the circularly polarized emission from a chiral emitter,^47^ while FDCD is probing differences in the excitation spectrum when the sample is irradiated with circularly polarized light, see Fig. 1.^48,49^ However, a bibliometric analysis of the available literature revealed that less than 70 studies have mentioned the term ‘fluorescence-detected circular dichroism’ in the abstract while over 1500 studies have mentioned ‘circularly polarized luminescence’ and more than 2600 studies have referred to the term ‘electronic circular dichroism’ (with over 60 000 mentioning ‘circular dichroism’, which is often used as a synonym for ECD).^50^ One of the first reports on FDCD dates back to 1974, where the chiral fluorophore tryptophan was selectively detected in a mixture containing the non-emissive chromophore cysteine.^38,48^ Later on, exciton coupled FDCD measurements were, for instance directed towards tertiary structure analysis of proteins,^51^ the stereochemical analysis of steroids covalently tethered to a porphyrin centre^38^ and for investigating the formation of DNA bichromophore assemblies.^52^

In this study, we investigated into fluorescence-detected circular dichroism (FDCD) spectroscopy for representative supramolecular host–guest systems and present a case study for the combined use of FDCD and ECD spectroscopy for characterising fluorescent supramolecular chiral systems.

## Results

### Compound selection

Self-assembled CB8·MDPP chemosensor and endo-functionalized molecular tube (MT) were chosen as achiral chromophoric and emissive hosts, and CB8 was used as a non-chromophoric achiral host for comparison. New planar-chiral paracyclophane dyes are introduced as chiral indicators. As guests, a wide range of biorelevant chiral compounds was used, including amino acids (*e.g.* Phe, Trp), amino acid derivatives, peptides and synthetic intermediates (chiral epoxides). The protein insulin, that bind to the host CB8·MDPP was also investigated. The chemical structures of the hosts, guests and dyes are depicted in Fig. 2.

### Synthesis of new chiral paracyclophane dyes

**Scheme 1:**
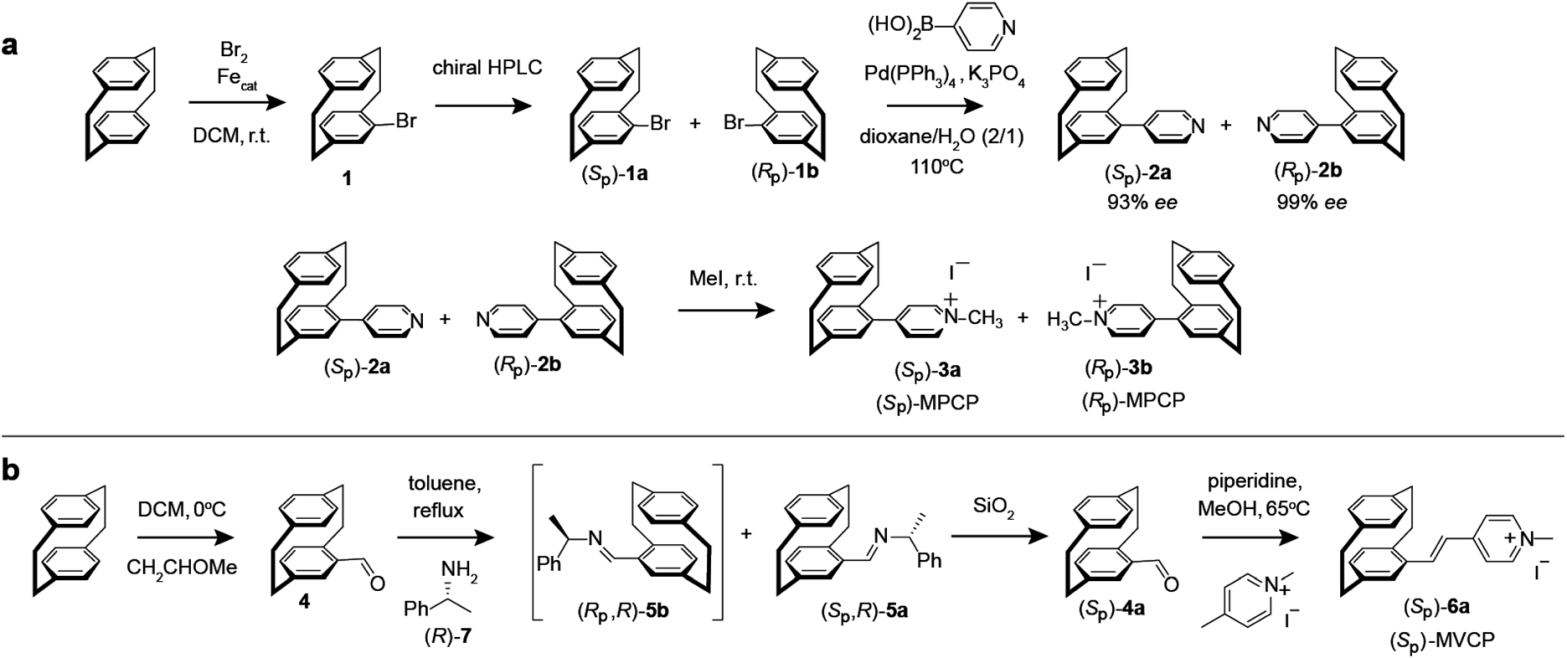
Synthesis route of (a) MPCP dye enantiomers, (*S*_P_)-MPCP and (*R*_P_)-MPCP and (b) enantiopure (*S*_P_)-MVCP dye.

The enantiomers of the paracyclophane-derived dye MPCP, (*S*_P_)-MPCP ((*S*_p_)-3a) and (*R*_P_)-MPCP ((*R*_p_)-3b) were prepared through a stepwise coupling procedure following the synthetic route shown in Scheme 1a. Starting from unsubstituted [2.2]paracyclophane, an aromatic substitution with bromine under iron catalysis resulted in (*rac*)-4-bromo[2.2]paracyclophane (1) in excellent yield,^53^ followed by a preparative HPLC with a chiral stationary phase^54^ to separate the two enantiomers of racemic 1, (*S*_p_)-1a and (*R*_p_)-1b. Subsequently a Pd-catalysed Suzuki cross-coupling of (*S*_p_)-1a and (*R*_p_)-1b with 4-pyridyl boronic acid was carried out to obtain (*S*_p_)-2a and (*R*_p_)-2b with 93% ee and 99% ee respectively and 79% yield. Afterwards, (*S*_p_)-2a and (*R*_p_)-2b was treated with methyl iodide in the final quaternization step in order to obtain the MPCP dye enantiomers (*S*_P_)-MPCP ((*S*_p_)-3a) and (*R*_P_)-MPCP ((*R*_p_)-3b) in 55% yield (see Section 4 in ESI† for the synthetic details).

The new, chiral paracyclophane dye, (*S*_P_)-MVCP ((*S*_p_)-6a) was designed in order to obtain a dye with longer excitation wavelength, following the synthetic route shown in Scheme 1b. 4-Formyl[2.2]paracyclophane 4 was initially obtained from unsubstituted [2.2]paracyclophane through a Rieche formylation in quantitative yield.^55^ The enantiomers of the aldehyde 4 were then prepared following the literature procedure,^56^*i.e.*4 was condensed with (*R*)-7 by refluxing in toluene to obtain the diastereomeric imines 5, which were then separated by fractional crystallization in *n*-hexane to obtain the analytically pure (*S*_p_,*R*)-5a in 27% yield. Acid-labile compound 5a was hydrolysed by filtration over a column of silica using dichloromethane as the eluent to obtain the enantiopure aldehyde (*S*_p_)-4a with 98% *ee*.^56^ (*S*_p_)-4a was then treated with 1,4-dimethylpyridinium iodide in a Knoevenagel condensation with piperidine in methanol to yield chiral paracyclophane (*S*_P_)-MVCP dye ((*S*_p_)-6a) in 22% yield (see Section 5 in ESI† for the synthetic details).

### General protocol for ECD and FDCD measurements

In contrast to ECD, measurement protocols for FDCD measurements were lacking in the literature, which was particularly hindering because FDCD is more complex and can be artefact-prone than ECD measurements (see below). We thus first optimized the measurement parameters for FDCD (procedures, parameters, conditions), providing a transferable and general FDCD measurement protocol which will be of utility even beyond supramolecular systems (see further below and Section 7 in ESI†).

It is well known that ECD measurements should be performed on samples with an absorbance value between 0.4 to 1 (theoretically a value of 0.87 is optimal for ECD measurements).^35,36^ Technically, ECD measurements are conducted at a fixed direct current (DC) voltage by automatically varying the high tension (HT) voltage on the photomultiplier (PM) tube of the ECD detector. The obtained ECD signal is proportional to the concentration of the chiral chromophore.

FDCD measurements can be performed on a CD spectrometer that is equipped with an FDCD accessory, for instance where a long-pass filter (LP-filter) and lenses are installed in a 90-degree geometry to the excitation light source, directing the fluorescence light towards an additional light detector, *e.g.* a PM tube,^57^ see also Fig. 1. Contrary to ECD, FDCD measurements are conducted at a varying DC voltage by fixing the HT voltage on the PM tube of the FDCD detector. There are two different fluorescent-based chiroptical parameters that can be obtained from fluorescence-detected circular dichroism spectroscopy. (i) The “differential circularly polarized fluorescence excitation” (often termed as Δ*F*) can be defined as the fluorescence intensity difference resulting from excitation with left (*F*_L_) and right (*F*_R_) circularly polarized light, see eqn (1). The Δ*F* value is concentration dependent and can be considered as the analogue to the ECD value (also known as the ellipticity, *θ*), even though both quantities have different units. (ii) In order to arrive at a concentration-independent quantity, the Δ*F* value can be normalized by the total fluorescence of the sample (*F*_L_ + *F*_R_), which is measured as the DC component, see eqn (2). This parameter is typically referred to as the FDCD value. Albeit the derivation is mathematically more complex, the FDCD value can be considered as the fluorescence-based chiroptical analogue of the concentration-independent molar circular dichroism (Δ*ε*), see also Section 7.3 in the ESI.†

1 Δ*F* = *F*_L_ − *F*_R_

2
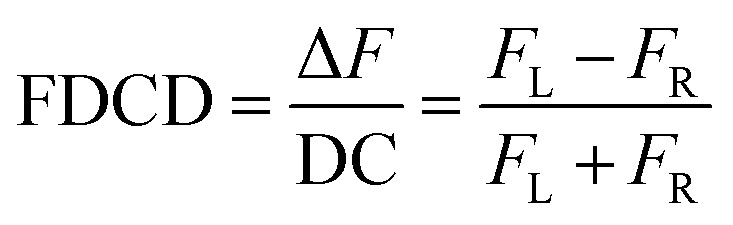


Practically, we recommend that the FDCD measurement set-up is made as such that both the Δ*F* signal and the DC voltage (= total fluorescence) are collected in two separate channels, and that these measurements are performed for both the sample and solvent (“blank”). The FDCD spectra are then obtained by first subtracting the solvent spectra from the sample spectra, arriving at baseline-corrected Δ*F* and DC spectra. From this, normalized FDCD spectra are obtained by dividing the baseline-corrected Δ*F* spectra by the corrected DC voltage.

Unlike ECD measurements, recording Δ*F* offers additional variability in choosing measurement parameters. For instance, the HT voltage can be tuned independently from the bandwidth (BW), through which the signal intensity can be adjusted and thereby a wider measurement range than available for ECD measurements can be reached – examples are shown in the next section.^38^ Few studies have focused on using the Δ*F* component of the FDCD data directly for sensing.^58,59^ Therefore, we first optimized the measurement parameters for the Δ*F* signal with respect to bandwidth and HT voltage. Recommended measurement options and empirical correlation curves between signal intensity, HT voltage and bandwidth are discussed in the ESI† (see Section 7). In general, we suggest that both Δ*F* and FDCD spectra are considered for data analysis and evaluation.

### Comparison of sensitivity for FDCD and ECD measurements

**Fig. 3:**
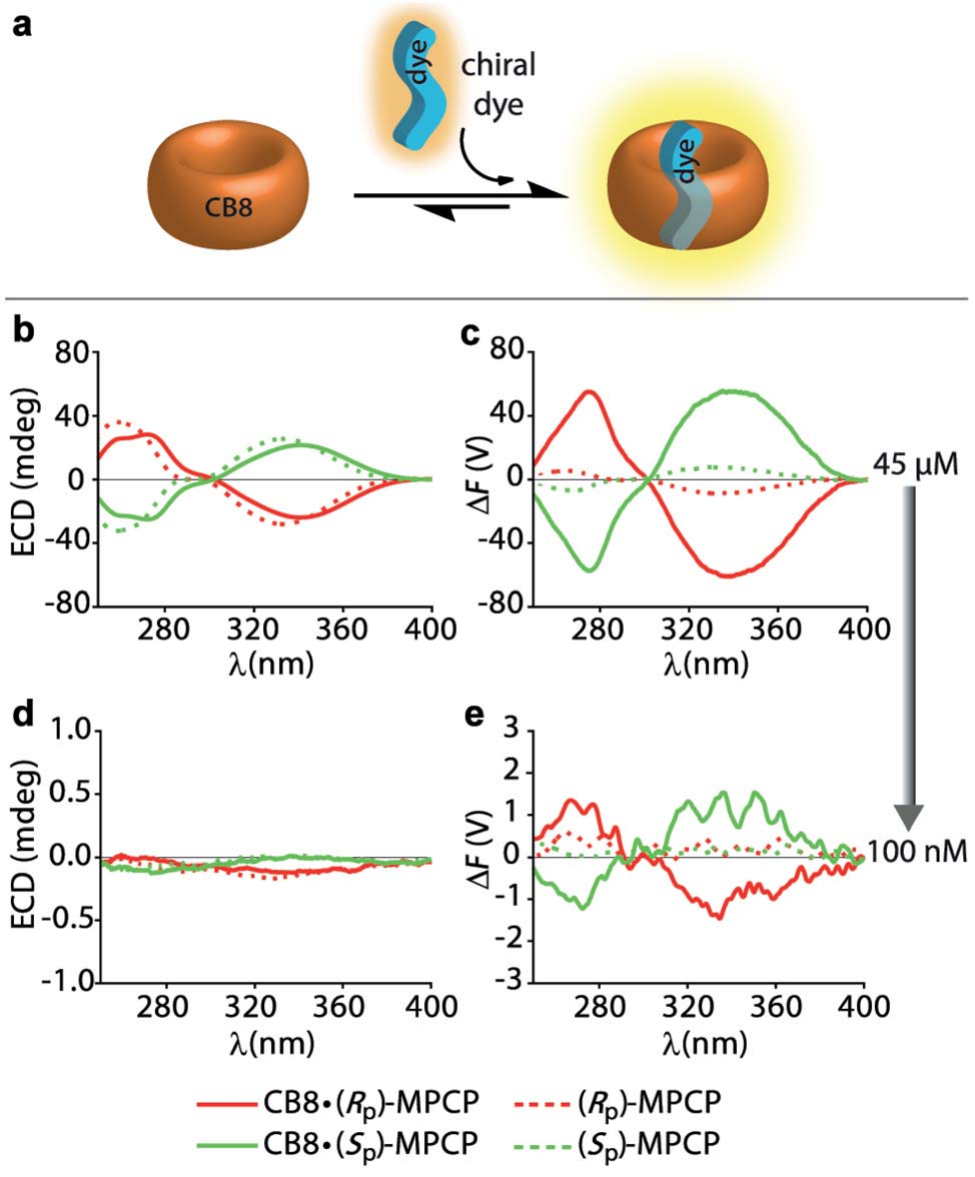
(a) Schematic representation of a 1 : 1 host–dye complex formation between the chiral chromophoric MPCP dye and the non-chromophoric host CB8. (b) ECD and (c) Δ*F* spectra of the MPCP dye enantiomers (*R*_p_)-MPCP (45 μM) and (*S*_p_)-MPCP (45 μM) in the absence (dashed lines) and presence of CB8 (solid lines) (45 μM) in water. Parameters used: HT = 650 V, BW = 4 nm, Acc = 20, LP-filter = 420 nm. (d) ECD and (e) Δ*F* spectra of MPCP dye enantiomers (*R*_p_)-MPCP (100 nM) and (*S*_p_)-MPCP (100 nM) in the absence and presence of CB8 (100 nM) in water. Parameters used: HT = 800 V, BW = 4 nm, Acc = 20, LP-filter = 420 nm. The measurement parameters, HT voltage (HT), bandwidth (BW), number of accumulations (Acc) as well as the long-pass filter (LP-filter) were kept constant for each set of experiments.

In order to compare the sensitivity of FDCD and ECD measurements, we first investigated a simple 1 : 1 host–dye complex of a chiral chromophoric dye with a non-chromophoric host (see Fig. 3a). As chiral chromophoric indicator dyes, the MPCP dye enantiomers, (*S*_P_)- and (*R*_P_)-MPCP were utilized with CB8 as the non-chromophoric host (see Fig. 2a and b). Both (*S*_P_)- and (*R*_P_)-MPCP were found to display strong ECD bands on account of their rigid, planar-chiral structure. Recently, we have shown that racemic MPCP forms an inclusion complex with CB8, which is extremely stable, *K*_a_ = 3.89 (±0.99) × 10^12^ M^−1^ in water.^53^ Thus, in the absence of competing guests the CB8·MPCP complex remains fully intact even upon dilution to the nanomolar concentration regime, which was verified by fluorescence titration experiments. This supramolecular system, extended now to the use of chiral dyes, therefore provided an ideal starting point to evaluate if the fluorescence-based Δ*F* quantity can be also under practical conditions more sensitive than ECD. At micromolar concentrations, both the Δ*F* and ECD spectra of the complexes of (*S*_P_)- or (*R*_P_)-MPCP and CB8 show clearly defined signals with maxima at 275 nm and 340 nm for the bound, and at 264 nm and 333 nm for the unbound guest, see Fig. 3b, c and S22.† Note the stronger signal differences in the Δ*F* than in ECD between CB8-bound and free MPCP; host–guest binding commonly leads to more pronounced changes in the emission than the absorbance spectra, and thus one can generally expect to find larger signal differences in Δ*F* than in ECD. This feature can be advantageous for sensing applications that rely on signal differences, *e.g.* Δ*F*-based quantification of the drug memantine through the CB8·MPCP reporter pair in blood serum, see further below. For comparing spectra obtained at different concentrations and measurement settings, the conversion of the Δ*F* spectra into concentration-independent FDCD spectra is useful; see Fig. S22 in the ESI.† These FDCD spectra can be related to the measured ECD spectra through converting each of them into molar circular dichroism (Δ*ε*) values, see Section 7.3 in the ESI.† Indeed, FDCD and ECD data conversion for the CB8·MPCP reporter pair yielded very similar Δ*ε*-spectra (Fig. S23†), as is theoretically expected.^38,48,49^

The new obtained (*S*_P_)-MVCP dye (see Fig. 2b) was also investigated to study its binding properties with CB8. (*S*_P_)-MVCP shows absorption bands in the 350 nm to 450 nm region, which is by 46 nm red-shifted absorption compared to the MPCP dye. Similar to MPCP, (*S*_P_)-MVCP shows a strong fluorescence enhancement on binding to CB8 (Fig. S24c†). At micromolar concentrations of (*S*_P_)-MVCP and CB8, both the Δ*F* and ECD spectra show a clearly defined signal with signal maxima at 299 nm and 386 nm for the bound, and at 285 nm and 363 nm for the unbound guest (Fig. S24†). Binding to CB8 also resulted in an enhancement in the Δ*F* signal accompanied by a bathochromic shift in both ECD and Δ*F* signals. The red-shifted absorption of CB8·(*S*_P_)-MVCP complex compared to CB8·MPCP can be advantageous for sensing studies in real biological media, see below.

The comparison of the resulting Δ*F* and ECD spectra for the CB8·MPCP system at submicromolar concentration are striking: while it is not possible to detect clear ECD signals at low concentrations (<1 μM), Δ*F* signals remain readily measurable even at 100 nM (Fig. 3d, e and S25†), pointing to at least an order of magnitude higher sensitivity of FDCD over ECD measurements. Δ*F* and FDCD spectra of the CB8·MPCP complex showed a more “noisy” character than ECD spectra, which also holds true for the other systems that we have investigated and that were reported in the literature.^38,58^ This obstacle can be overcome by either increasing the number of accumulations (Acc) in the measurement (Fig. S25c and d†) or by utilizing Δ*F* in the single-wavelength measurement mode, as is pointed out on several occasions in the main text and ESI.† For *e.g.*, investigations of the CB8·(*R*_p_)-MPCP and CB8·(*S*_p_)-MPCP complexes upon dilution to 50 nM provided single-wavelength Δ*F* values that were reproducible and consistent. As an additional test, the calculated FDCD value was constant across a wide concentration range, ruling out photophysical or other artefacts. Conversely, no meaningful ECD signal could be reproducibly obtained in the nanomolar concentration range (see Tables S2 and S3 in ESI†).

**Fig. 4:**
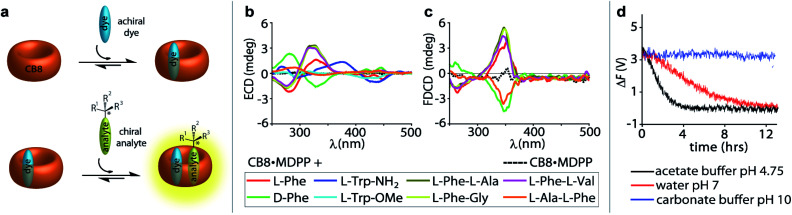
(a) Schematic representation of a 1 : 1 : 1 ternary complex formation between the achiral chromophoric CB8·MDPP receptor and the chiral analyte. (b) ECD and (c) FDCD spectra of CB8·MDPP receptor (20 μM) in the absence (dashed lines) and presence (solid lines) of several amino acids, amino acid derivatives and dipeptides (50 μM) in water. Parameters used: HT = 520 V and 510 V (for dipeptides), BW = 4 nm, Acc = 20, LP-filter = 515 nm. (d) Time course Δ*F* measurement of CB8·MDPP (20 μM) in the presence of (1*R*,2*R*)-PPO (100 μM) in different solvent systems: 50 mM acetate buffer at pH 4.75 (black), water at pH 7 (red) and 50 mM carbonate buffer at pH 10 (blue) when monitored at 350 nm. Parameters used: HT = 520 V, BW = 4 nm, Acc = 20, LP-filter = 515 nm, data pitch = 5 s, D. I. T = 8 s, *t*_measure_ = 14 h. The Δ*F* signals were corrected for the photoselection artefacts arising from the CB8·MDPP receptor, see Section 9.1.1 in ESI.†

The sensitivity of FDCD *vs.* ECD measurements was also assessed for self-assembled chemosensors composed of CB8 and the dicationic racemic dye MDPP (see Fig. 2a and b), which are known to subsequently bind chiral aromatic guests, *e.g.* Phe, Trp and many other aryl-moiety containing species.^14^ Our previous investigations have shown that these 1 : 1 : 1 hetero-ternary complexes (see Fig. 4a) show characteristic induced ECD signals, which can be used for analyte differentiation and reaction monitoring at micromolar concentrations (20 to 500 μM).^14^ Full spectral measurements and single-wavelength Δ*F* recordings were then utilized to compare the sensitivity of Δ*F* over ECD measurements (Fig. S27, Tables S4, S5, S8 and S9 in ESI†). Again, a remarkably higher sensitivity of Δ*F* over ECD was evidenced by the results, where only Δ*F* measurements gave reliable values at the low micromolar concentration regime; see for instance Table S4† that depicts the data for CB8·MDPP receptor complex with the peptidic guest l-Phe-l-Ala. Excess of the chiral guest over the host was used in these examples to ensure a sufficient degree of complexation of the chromophoric and emissive host.

Because achiral chromophoric/emissive hosts do not contribute to the chiroptical signals, they can be used in excess in both ECD and FDCD measurements to enhance the degree of analyte complexation, see for instance the ECD- and Δ*F*-monitored titration of CB8·MDPP receptor to l-Phe–Gly in Fig. S28.† A rise of the ECD and Δ*F* values by increasing the receptor concentration was observed until the degree of complexation reached unity, beyond which the ECD and Δ*F* signals saturated. Conversely, in conventional absorbance- or emission-based assays, such an approach is generally infeasible as it causes an undesirable signal increase proportional to the concentration of unbound host. For instance, the fluorescence intensity displayed a rather linear increase for the aforementioned system (Fig. S28c†).

### Detection of chiral chromophoric analytes as well as label-free endpoint and continuous reaction monitoring

Combining FDCD and ECD measurements is potentially more informative, thereby providing additional, useful chiroptical information about the analyte present than the individual methods on their own. We evaluated this for the system composed of the achiral chromophoric CB8·MDPP chemosensor interacting with chiral Phe- and Trp-containing species resulting in 1 : 1 : 1 hetero-ternary complexes in aqueous media (see Fig. 4a).^14^ Unlike the results obtained in ECD measurements where all combinations gave rise to chiroptical signals, FDCD is far more selective, where only the combination of CB8·MDPP with Phe but not with Trp species gave rise to induced chiroptical FDCD effects (Fig. 4b, c and S26†). This can be understood because Trp quenches the emission of CB8·MDPP while Phe binding leads to a slight bathochromic shift and emission increase (Fig. S26c†). Furthermore, it was found that N-terminal Phe containing dipeptides can be distinguished from C-terminal Phe variants both by FDCD and ECD, see Fig. 4b, c and S27.† (However, different Phe–X dipeptides remained indistinguishable by both techniques utilizing CB8·MDPP as the host.) Combined information of FDCD and ECD measurements were for instance useful to verify that the CB8·MDPP receptor targets phenylalanine and not the multiple tyrosine residues present in the protein insulin (see the amino acid sequence shown in Fig. 2c), because binding to tyrosine would have resulted in no FDCD signals due to emission quenching,^60^ see further below. This finding agrees with the binding geometry deduced from the crystal structure of the host CB7 with insulin.^61^

Practically important, the combined use of FDCD and ECD will be advantageous for a sensitive and selective endpoint and label-free continuous reaction monitoring of (bio)chemical and (bio)physical processes in real time. Several examples for both purposes, utilizing supramolecular receptors *e.g.* cucurbit[*n*]urils,^62–64^ calix[*n*]arenes,^64,65^ molecular tubes,^11,66^ and CB8·dye chemosensors^14,60^ reported so far are based on absorbance, emission and ECD spectroscopy. We demonstrate here representative examples for the use of FDCD in combination with supramolecular hosts for (i) endpoint and (ii) continuous reaction monitoring:

(i) The racemisation of amino acids remains an important obstacle under synthetic conditions in organic solvents. In our previous study we have adopted ECD measurements in the presence of CB8·MDPP for monitoring the base-catalysed racemisation of both l-Phe and l-Phe–Gly in water, DMF and ethylene glycol at elevated temperatures.^15^ Herein, the Δ*F* signal was utilized for the endpoint monitoring of racemisation of amino acids using the CB8·MDPP reporter pair. In practice, aliquots of the reaction mixture were added into an aqueous solution of the CB8·MDPP chemosensor, and the Δ*F* and ECD signal at 338 nm (for l-Phe) and 333 nm (for l-Phe–Gly), was measured before and after the chemical reaction (Fig. S29 and S30†). Pleasingly, the influence of the reaction conditions on the racemisation of amino acids monitored *via* Δ*F* measurements are in full agreement with the more cumbersome HPLC-based literature procedure.^67^ The chemosensor-based approach verifies that water suppresses the racemisation of l-Phe and l-Phe–Gly while DMF leads to the fast loss of the chirality. Under the conditions tested, both Δ*F* and ECD were equally suitable. However, Δ*F* has the additional advantage of its higher sensitivity, *e.g.* for detections at lower concentration ranges.

(ii) If the supramolecular chemosensors are compatible with the reaction conditions (solvent, pH, additives, temperature *etc.*) they can also be directly added to the reaction mixture and a continuous signal read out can be recorded. For instance, adding 20 μM of CB8·MDPP receptor to 100 μM (1*R*,2*R*)-1-phenylpropylene oxide ((1*R*,2*R*)-PPO) in deionized water (pH 7), 50 mM acetate buffer (pH 4.5) and 50 mM carbonate buffer (pH 10) allowed for *in situ* monitoring of the hydrolysis of the epoxide. Both time course Δ*F* and ECD spectral measurements revealed that complete hydrolysis and racemisation occurred in acidic environment after 4 h and under neutral conditions after 12 h, while the epoxide did not hydrolyse under basic conditions, see Fig. 4d, S31 and S32.† This observation is in agreement with expectations for secondary-carbon containing epoxides that likely follow a proton catalysed S_N_1 type hydrolysis mechanism (see Scheme S1 in ESI†).^68,69^

Similarly, the hydrolysis of phenyl-β-d-galactopyranoside by β-galactosidase, which is a commonly studied reaction in cell biology,^70,71^ can be monitored with the help of CB8·MDPP receptor by Δ*F* and ECD recordings. The time course Δ*F* and ECD measurements shows a gradual decrease in the intensity of both induced Δ*F* and ECD signals upon addition of β-galactosidase, enabling real time monitoring of the enzymatic conversion (Fig. S33 and S34†).

### Uncovering of hidden aggregation phenomena by FDCD

**Fig. 5:**
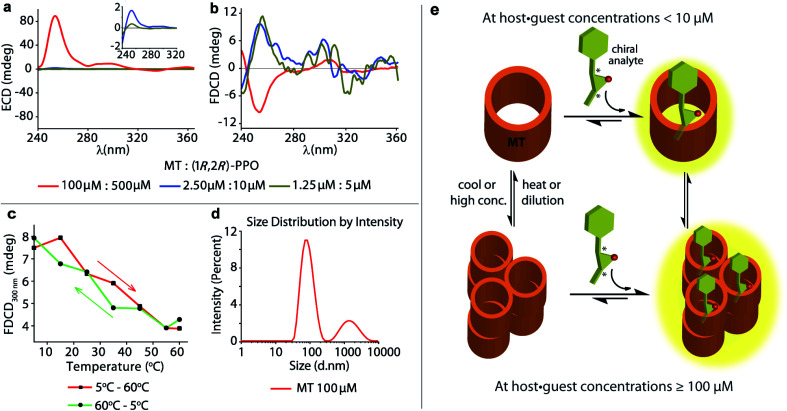
Concentration dependent (a) ECD and (b) FDCD spectra of freshly prepared MT receptor in presence of (1*R*,2*R*)-PPO in water. The inset show the zoomed in ECD signals in the 240 nm to 320 nm region. Parameters used: BW = 4 nm, Acc = 20, LP-filter = 380 nm. (c) FDCD signal at 300 nm of the MT receptor (100 μM) in water on increasing the temperature from 5 °C to 60 °C (red line) and on cooling the solution back from 60 °C to 5 °C (green line). (d) DLS measurements showing intensity distribution *versus* particle diameter of MT receptor solution (100 μM) in water. Parameters used: material RI = 1.45, dispersant RI = 1.330, viscosity = 0.8872 cP, measurement position = 4.65 mm, attenuator = 11. (e) Schematic representation showing the complex formation between the achiral chromophoric MT host and the chiral epoxides in their non-aggregated state at low concentrations and upon aggregation at higher concentrations and lower temperature.

The achiral emissive endo-functionalized molecular tube MT (see Fig. 2a) is known to bind selectively polar guests with hydrogen-bond accepting capacities such as dioxane, esters and epoxides in aqueous media with *K*_a_ up to 10^5^ M^−1^.^11,72,73^ For instance, both the chiral guest (1*R*,2*R*)-1-phenylpropylene oxide ((1*R*,2*R*)-PPO) and its enantiomer (1*S*,2*S*)-1-phenylpropylene oxide ((1*S*,2*S*)-PPO) are complexed with *K*_a_ = 8.97 (±0.9) × 10^4^ M^−1^ by MT in water.^11^ This interaction gives rise to a strong ECD signal,^11^*e.g.* approx. ±89 mdeg at 254 nm at 100 μM host and 500 μM of the guest.^11^ Applying FDCD measurements to this system, we were surprised that the induced FDCD signals were markedly weaker, approx. ±10 mdeg at 254 nm at the same concentrations, see Fig. 5a, b and S35.† Besides, the FDCD spectra of the control experiment with achiral MT receptor showed pronounced signals in the excitation peak region for the achiral host alone (see Fig. S35d†), which – of course – do not reflect chiroptical properties but must arise from anisotropic excitation, also known as photoselection, and from instrument-related artefacts.^38,74,75^ In essence, the larger the emissive compound, *e.g.* the macrocyclic host, and the higher the viscosity of the medium, the larger photoselection artefacts will be observed due to restricted rotation of the emitter^38,76^ (see Section 9.1 in ESI† for a detailed explanation on photoselection artefacts in FDCD and suggested artefact-subtraction procedures). Striking changes to the FDCD and ECD spectra were observed upon dilution. A strong reduction in the ECD signals corresponding to the decrease in host–guest concentration was observed, while the FDCD signal even inverted its sign upon dilution, see Fig. 5a, b, S36 and S37.† The influence of photoselection artefacts or anisotropy of the system in the measured ECD and FDCD spectra at higher concentrations were evaluated by measuring the linear polarization components, *i.e.* linear dichroism (LD)^77,78^ and linear birefringence (LB)^78,79^ in case of ECD, and fluorescence-detected linear dichroism (FDLD)^38,80^ in case of FDCD (Fig. S39 and S40†). The measured LD and LB values are only modest in magnitude, indicating that the observed ECD signal are direct measure of the chiroptical behaviour of the system. In contrast, FDLD spectra showed strong signals in the region where the FDCD bands are found for the achiral MT receptor (Fig. S40†), indicating a strong influence of fluorescence anisotropy. Clearly, the FDCD signal should be considered for this system as an apparent one which does not directly report on chiroptical properties but also contains other contributions that are indicative of fluorophore orientation. Thus, measurements for the MT·(1*R*,2*R*)-PPO complex were also carried out on an artefact-free FDCD spectrometer utilizing an ellipsoidal mirror,^81^ at receptor concentration of 100 μM. In this case, the FDCD spectrum obtained at a high micromolar concentration (100 μM) resembles the FDCD spectrum collected at lower concentrations on the standard FDCD spectrometer (Fig. S36 and S43†). We concluded that something unusual occurs at higher concentration of MT and guests that was undetected previously by ECD, fluorescence, absorbance and NMR measurements.^11^ We propose that MT and the MT·PPO complexes form supramolecular aggregates at higher micromolar concentrations (see Fig. 5e) that lead to an enhanced ECD signal and sizeable fluorescence-detected linear dichroism contributions to the FDCD band. MT is hydrophobic, such that aggregation formation in water is plausible. Additional experiments support the aggregation hypothesis: Dynamic Light Scattering (DLS) data (Fig. 5d and S48†) and FDCD measurements at longer time intervals (Fig. S44†) revealed an “aging phenomenon” of the MT receptor stock solution. Moreover, the observed FDCD signals for the MT receptor were temperature-dependent (Fig. 5c and S45†) and that the fluorescence intensity–concentration plot shows a concave curvature (Fig. S46†). Concentration-dependent DOSY NMR spectra in D_2_O displayed a slower diffusion rate of MT at higher concentrations (Fig. S47†), which also support the aggregation hypothesis. Clearly, FDCD-based investigations can uncover interesting supramolecular phenomena that were invisible to ECD and other spectroscopic techniques alone.

### Background reduction in complex systems and chromophoric biofluids

**Fig. 6:**
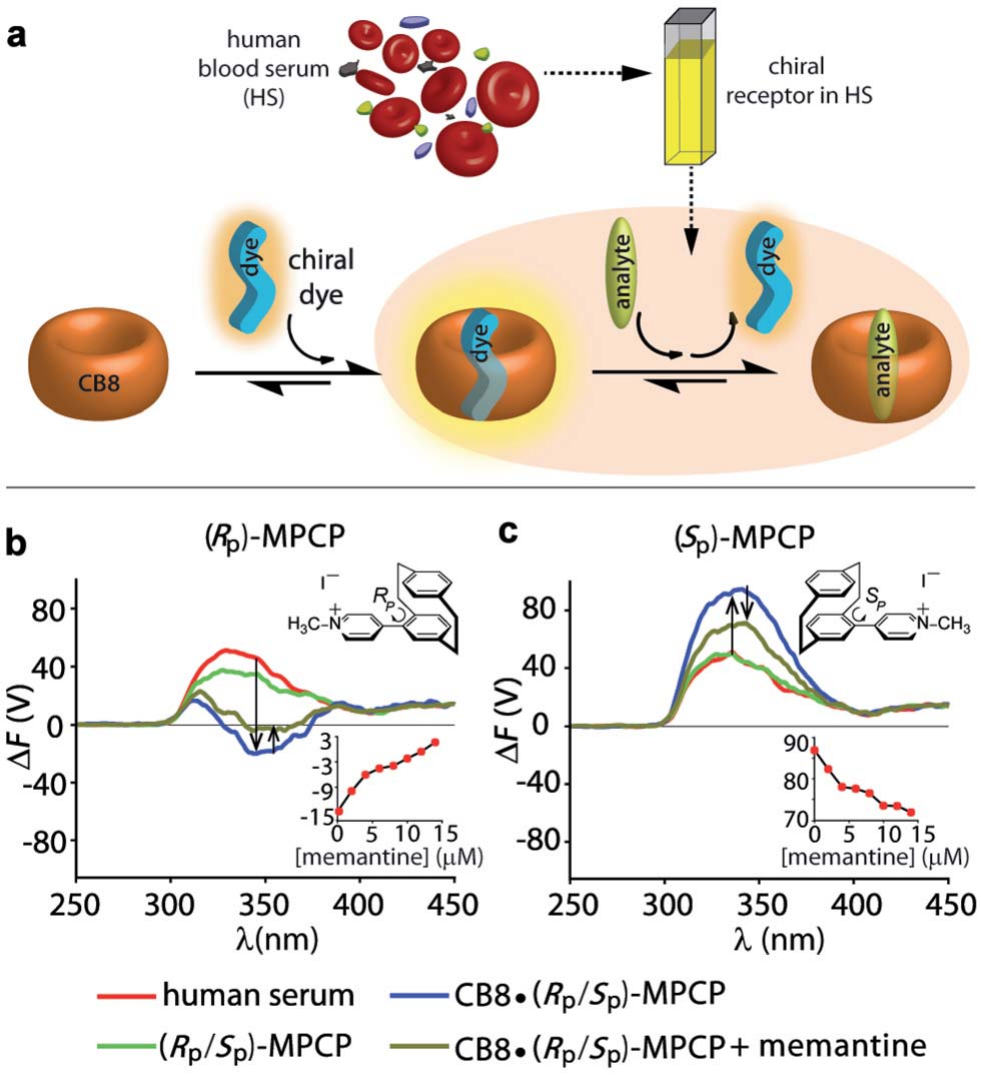
(a) Schematic representation depicting the formation of a 1 : 1 CB8·MPCP complex, followed by the displacement of MPCP from the host cavity upon addition of a higher binding analyte like memantine. Δ*F* spectra of (b) CB8·(*R*_P_)-MPCP (20 μM) and (c) CB8·(*S*_P_)-MPCP (20 μM) in human blood serum before and after addition of memantine (20 μM). Parameters used: HT = 800 V, BW = 4 nm, Acc = 20, LP-filter = 515 nm. The insets show the variation in Δ*F* signal at 340 nm on stepwise addition of memantine.

It is now shown that the host–guest binding coupled FDCD technique can provide background-reduced signals compared to ECD and standard absorbance or fluorescence measurements. This is of relevance if a chiral analyte should be detected in the presence of chiral (and chromophoric) substances (*e.g.* proteins, DNA) or complex media such as biofluids that show strong absorbance, emission and ECD signals. As model cases, two supramolecular systems were investigated:

(i) The binding of the protein insulin by the chemosensor CB8·MDPP can be followed by ECD,^16^ but one finds some contribution from the ECD signal of the protein at <310 nm, see Fig. S49 in ESI.† Conversely, for FDCD, a protein-based chiroptical signal contribution was completely absent when utilizing a 515 nm long-pass filter and only the induced FDCD signal from CB8·MDPP complex bound to and located in the chiral protein-environment is observed (Fig. S49†).

(ii) To showcase the power of FDCD-based detection schemes, human blood serum (HS) was utilized as strongly chromophoric and autoemissive biofluid that contains many chiral components. We have recently introduced the first emission-based supramolecular assay for the detection of Alzheimer's drug memantine in human blood serum using the aforementioned CB8·MPCP reporter pair.^53^ In the fluorescence-based assay mode, alterations of the total fluorescence background due to sample-to-sample differences can complicate the situation. With the FDCD technique, that is selectively reporting only on chiral and emissive species, the background was expectedly much lower than for conventional emission or ECD spectroscopy. In fact, only by Δ*F* and not by ECD, was it possible to quantitatively detect the achiral drug memantine in human blood serum in the low micromolar concentration regime (Fig. 6a, c and S50–S53†). For instance, the addition of 15 μM of memantine to a solution of CB8·(*R*_P_)-MPCP or CB8·(*S*_P_)-MPCP (reporter pair at 20 μM) in human blood serum led to a Δ*F* signal change by +16 V or −15 V respectively, upon displacement of the chiral emissive dye from the CB8 cavity, while the ECD signals did not show any significant alteration (Fig. 6b, c and S50–S53†). Curiously, in the case of using the enantiomeric indicator dyes (*S*_P_)-MPCP and (*R*_P_)-MPCP the behaviour was similar but not identical, see the ECD and Δ*F* spectra and titration curves in Fig. 6b, c and S50–S53 in ESI.† These differences may be originating from the diastereomeric interaction of the chiral indicator dye MPCP with the chiral components in blood serum, *e.g.* human serum albumin and other chiral proteins with large binding pockets. For developing practical assays in biofluids containing chiral compounds, it is therefore advisable to access both chiral forms of a dye or host, as the combined chiroptical information obtained by both enantiomers can enrich the understanding of the system and help to identify artefacts.

Both CB8·(*R*_P_)-MPCP and CB8·(*S*_P_)-MPCP are from a photophysical point of view not ideally suited for sensing in HS because the ECD and Δ*F* signals of the complex lie in the same region as the background Δ*F* and ECD signals arising from HS alone. The CB8·(*S*_P_)-MVCP reporter pair, which shows a more red-shifted absorption compared to the CB8·MPCP system, therefore appears to be an interesting candidate for sensing studies in human blood serum. The addition of 16 μM of memantine to a solution of CB8·(*S*_P_)-MVCP (reporter pair at 20 μM) in human blood serum led to a Δ*F* signal change by −4 V at 407 nm monitoring wavelength, see Fig. S54 and S55 in ESI.† Interestingly, when using CB8·(*S*_P_)-MVCP instead of CB8·(*S*_P_)-MPCP as a reporter pair, also ECD-based detection of memantine in blood serum is feasible, see Fig. S54 and S55 in ESI.†

## Discussion

The commonly applied chiral-analytical tools in drug discovery, molecular configurational and conformational analysis include ECD, chiral HPLC, NMR, Raman optical activity (ROA) and optical rotatory dispersion (ORD). However, despite being widely used, these methods come with their own drawbacks. For example, NMR and chiral HPLC analysis require access to high-cost equipment and are not suitable for high-throughput measurements. Another important limitation is the low sensitivity, *e.g.* of ORD, thereby requiring high sample concentration for the measurements. This has impeded the investigation of biological analytes at their clinically relevant concentration levels in solution, as well as of large biomolecules and aggregation-prone systems that needs to be measured at low concentrations. Furthermore, real biological media that contain a range of chromophoric chiral substances give rise to strong signal backgrounds in the ECD, ROA and ORD spectra, making the analysis practically difficult. Most of these limitations can be overcome by the use of emission based spectroscopic techniques such as FDCD and CPL, which combine the advantages of both chiroptical and fluorescence techniques. FDCD (and CPL) possess additional merit for sensing applications in (complex) biofluids as only the compounds that are both chiral and fluorescent give rise to an emission-based chiroptical signal.^82–84^ We believe that FDCD has an edge over CPL for the differentiation of analytes by spectroscopic fingerprints: firstly, excitation spectra are typically structured and reflect several S_0_ → S_*n*_ (*n* ≥ 1) transitions while emission spectra arise usually only from the S_1_ → S_0_ (or T_1_ → S_0_) transition(s) and are typically featureless.^57,85^ Secondly, FDCD measurements can be readily obtained (even simultaneously to ECD) by upgrading commercial ECD spectrometers with a FDCD set-up,^57,58,86^ while CPL measurements are often difficult to carry out as they require purchase of a high-cost stand-alone CPL equipment or specialized expertise to construct home-build CPL accessories.^58,87–89^ Despite these potential advantages, FDCD measurements had only rarely been performed, mostly on chiral chromophores,^90,91^ protein–ligand^51,92,93^ and nucleobase systems.^94,95^

The sensitivity of a supramolecular assay is obviously also limited by the degree of complexation, which is a function of the binding affinity and concentration of the host and analyte. In spectroscopic assays, the common strategy to enhance the degree of complexation is the increase of the concentration of the spectroscopically silent component, which is mostly the analyte. However, in some cases this strategy is flawed, *e.g.* if the spectroscopically silent component is expensive, shows solubility limitations,^96^ shows undesirable aggregation tendencies (see above) or is present in very low concentration in the analytical sample.^15,53^ In contrast, FDCD analysis can conveniently be carried out in the presence of excess of fluorescent host, allowing for an increase of the degree of complexation of the chiral target analyte. Thus, FDCD-based sensing can be a convenient choice to study the interaction of chromophoric receptors with biomolecules or proteins whose concentration can be kept low. For comparison, in standard fluorescence-based assay, it is generally infeasible to use a high excess of the fluorescent compound. The design of new novel chiral indicator dyes as well as protein- and analyte-binding emissive hosts with improved photophysical properties will increase the scope of FDCD-based (and ECD-based) supramolecular applications in biorelevant media.

## Conclusion

Herein, we provide for the first time an exploration of FDCD measurements on (synthetic) supramolecular complexes in aqueous media. At least an order of magnitude higher sensitivity can be exploited with FDCD compared to ECD measurements, and even the nanomolar sensitivity can be reached by FDCD in favourable circumstances, which is beyond the scope of most other techniques used for characterization of supramolecular systems. The high sensitivity of FDCD will be advantageous for sensing in real biological media where the analytes are mostly present in the low micromolar to nanomolar concentration regime.

In addition to a sensitivity enhancement, the combined use of FDCD and ECD measurements can uncover supramolecular processes, *e.g.* aggregation phenomena and provide complementary information that is useful for distinguishing chiral analytes from each other, and through which the target binding sites of the host can be identified, *e.g.* Phe- from Trp residues in proteins. FDCD spectra can be informative, but it is important to consider anisotropic excitation/photoselection and instrument-related artefacts that can cause apparent FDCD signals, unless a dedicated artefact-free FDCD setup is utilized. This work also established FDCD measurements for label-free reaction monitoring, both in an endpoint assay version and for continuous reaction monitoring even in the presence of other chromophoric compounds, as found in biofluids such as human blood serum, which can be employed for faster and more facile analysis of chemical or enzymatic transformations compared to other established methods such as HPLC-MS.

In summary, FDCD was shown to be a highly sensitive and information-rich spectroscopic method for the selective chiroptical analysis of fluorescent supramolecular host–guest systems both in aqueous solutions and biorelevant media. This work not only established the use of FDCD (and ECD) for a range of host–guest and host–protein complexes, but also provides a comprehensive user guide and recommendation for the most effective and general use of FDCD spectroscopy. We believe that the combined use of FDCD and ECD has a wide applicability and can be readily applied to other supramolecular chiral systems.

## Abbreviations

ECD: Electronic circular dichroism
FDCD: Fluorescence-detected circular dichroism
CPL: Circularly polarized luminescence
LD: Linear dichroism
LB: Linear birefringence
FDLD: Fluorescence-detected linear dichroism
CP: Circularly polarized
DC: Direct current
HT: High tension
LP-filter: Long-pass filter
PM: Photomultiplier
BW: Bandwidth
Acc: Accumulations
D. I. T: Digital integration time
*t*_measure_
: Measuring time
NMR: Nuclear magnetic resonance
DOSY: Diffusion-ordered spectroscopy
DLS: Dynamic light scattering
HPLC-MS: High performance liquid chromatography-mass spectrometry
MT: Endo-functionalized molecular tube
Trp: Tryptophan
Phe: Phenylalanine
PPO: 1-Phenylpropylene oxide
MPCP: *N*-Methyl-4-pyridinylium[2.2]paracyclophane
(*S*_*P*_)-MVCP: (*E*)-4-((*S*_*P*_)-4-Vinyl [2.2]paracyclophane)-1-methyl pyridin-1-ium

## Data availability

Data for this paper, including synthesis of the chiral paracyclophane type indicator dyes are available at Chemotion at https://dx.doi.org/10.14272/reaction/SA-FUHFF-UHFFFADPSC-BAIBHOHKSY-UHFFFADPSC-NUHFF-NUHFF-NUHFF-ZZZ.3 and https://dx.doi.org/10.14272/reaction/SA-FUHFF-UHFFFADPSC-GRWXNDHDQX-UHFFFADPSC-NUHFF-MABGN-NUHFF-ZZZ. In addition, the datasets for all the spectra in the manuscript are provided as additional supplementary material.

## Author contributions

Amrutha Prabodh: conceptualization, methodology, general measurement protocol and user guide for FDCD measurements, design of experiments and experimental investigation, validation, data analysis, visualization, manuscript writing. Yichuan Wang: synthesis and characterization of (*S*p)-MVCP dye. Stephan Sinn: conceptualization, methodology, data analysis. Paolo Albertini and Christian Spies: experimental investigation and cross-validation of selected systems, LD, LB, FDLD and fluorescence anisotropy measurements and analysis. Eduard Spuling: synthesis and characterization of MPCP dye enantiomers. Liu-Pan Yang: synthesis and characterization of MT receptor, DOSY NMR measurements for MT aggregation study. Wei Jiang: conceptualization, supervision, and funding acquisition. Stefan Bräse: conceptualization, supervision, and funding acquisition. Frank Biedermann: conceptualization and methodology, supervision, manuscript writing, funding acquisition. All authors contributed to the appropriate data analysis and in manuscript review and editing.

## Conflicts of interest

There are no conflicts to declare.

## Supplementary Material

SC-012-D1SC01411K-s001
