# Supplementary material for: Fluorescence detected circular dichroism (FDCD) for supramolecular host–guest complexes
Source: Chem Sci. 2021 Jun 9;12(27):9420–31. doi: 10.1039/d1sc01411k (PMC8278969; doi:10.1039/d1sc01411k)
Supplement: SC-012-D1SC01411K-s001 [file SC-012-D1SC01411K-s001.pdf]

Electronic Supplementary Material (ESI) for Chemical Science.
This journal is © The Royal Society of Chemistry 2021

ELECTRONIC SUPPORTING INFORMATION

Fluorescence-Detected Circular Dichroism (FDCD) for Supramolecular Host-Guest Complexes

Amrutha Prabodh,[a] Yichuan Wang,[a, b] Stephan Sinn,[a] Paolo Albertini,[c] Christian Spies,[d] Eduard Spuling,[b] Liu-Pan Yang,[e] Wei Jiang,[e] Stefan Bräse,[b,f*] and Frank Biedermann[a*]

a A. Prabodh, Y. Wang, Dr. S. Sinn, Dr. F. Biedermann
Karlsruhe Institute of Technology (KIT), Institute of Nanotechnology (INT), Hermann-von-Helmholtz-Platz 1, 76344 Eggenstein-Leopoldshafen, (Germany)
E-Mail: frank.biedermann@kit.edu

b Y. Wang, E. Spuling, Prof. S. Bräse
Karlsruhe Institute of Technology (KIT), Institute of Organic Chemistry, Fritz-Haber-Weg 6, 76131 Karlsruhe (Germany)
E-Mail: stefan.braese@kit.edu

c P. Albertini
JASCO Europe srl, via Luigi Cadorna 1, 23894 Cremella (Italy)

d C. Spies
JASCO Deutschland GmbH, Robert-Bosch-Str. 14, 64319 Pfungstadt (Germany)

e L. Yang, W. Jiang
South University of Science and Technology of China, Department of Chemistry, Xueyuan Boulevard 1088, Nanshan District, Shenzhen 518055, China

f Prof. S. Bräse
Karlsruhe Institute of Technology (KIT), Institute of Biological and Chemical Systems – Functional Molecular Systems (IBCS-FMS), Hermann-von-Helmholtz-Platz 1, 76344 Eggenstein-Leopoldshafen (Germany).

Table of Contents

1. Abbreviation 3
2. Materials 5
3. Instrumentation 5
4. Synthesis of (S_p)-MPCP (3a) and (R_p)-MPCP (3b) 7
4.1. Synthesis of (*rac*/S_P/R_P)-4-bromo[2.2]paracyclophane (1, 1a and 1b) 7
4.2. Synthesis of (S_P/R_P)-4-(4'-pyridyl)[2.2]paracyclophane (2a and 2b) 10
4.3. Synthesis of (S_P/R_P)-N-methyl-4-pyridylium[2.2]paracyclophane iodide ((S_p)-MPCP (3a) and (R_p)-MPCP (3b)) 13
5. Synthesis of (S_p)-MVCP (6a) 15
5.1. Synthesis of (*rac*)-4-formyl[2.2]paracyclophane (4) 15
5.2. Synthesis of (S_P,*R*)-[N-1-(phenylethyl)]-4-[2.2]paracyclophanyl methanimine (5a) 17
5.3. Synthesis of (S_P)-4-formyl[2.2]paracyclophane (4a) 19
5.4. Synthesis of (*E*)-4-((S_P)-4-vinyl[2.2]paracyclophane)-1-methyl pyridin-1-ium iodide((S_p)-MVCP (6a)) 19
6. Sample Preparation 22
7. FDCD characteristics 24
7.1. General measurement protocol for FDCD measurements 24

7.1.1. User guide for FDCD measurements 25
7.2. Comparison of Δ*F* signal collected at different HT voltages and correction function 26
7.3. Conversion of ECD and FDCD ellipticity values to molar circular dichroism values 29
8. FDCD Measurements 31
8.1. Comparison of sensitivity for FDCD and ECD measurements and their combined use for detection of chiral analytes 31
8.1.1. Spectra of chiral MPCP and MVCP dye with CB8 receptor 31
8.1.2. Analyte spectra with CB8•MDPP receptor 36
8.1.3. FDCD and ECD measurements for rapid, label-free end-point and continuous reaction monitoring using CB8•MDPP receptor 43
8.1.3.1. Racemisation of amino acids and dipeptides 43
8.1.3.2. Hydrolysis of chiral epoxides 45
8.1.3.3. Monitoring of enzymatic reactions 47
8.2. Uncovering of hidden aggregation phenomena by FDCD 49
8.2.1. Analyte spectra with MT receptor 49
8.2.2. Aggregation Studies of MT receptor 60
8.3. FDCD measurements for background reduction in complex systems and chromophoric biofluids 66
8.3.1. Detection of insulin by CB8•MDPP receptor 66
8.3.2. Detection of memantine in blood serum by CB8•MPCP and CB8•MVCP receptor 68
9. FDCD limitations and alternatives 73
9.1. Photoselection artefacts in FDCD 73
9.1.1. Correction of signal artefacts in FDCD 76
9.2. Comparison of FDCD signal for spectra measured at different spectrometers 77
10. References 79

1. Abbreviation

ECD	electronic circular dichroism
FDCD	fluorescence-detected circular dichroism
LD	linear dichroism
FDLD	fluorescence-detected linear dichroism
LB	linear birefringence
CB8	cucurbit[8]uril
MT	molecular tube
MDPP	*N*,*N'*-dimethyl-2,9-diazaperopyrenium dication
(R_p)-MPCP	(R_p)-*N*-methyl-4-pyridinylium[2.2]paracyclophane
(S_p)-MPCP	(S_p)-*N*-methyl-4-pyridinylium[2.2]paracyclophane
(S_p)-MVCP	(*E*)-4-((S_P)-4-vinyl[2.2]paracyclophane)-1-methyl pyridin-1-ium
D-Phe	D-phenylalanine
L-Phe	L-phenylalanine
L-Phe-L-Ala	L-phenylalanyl-L-alanine
L-Phe-Gly	L-phenylalanylglycine
L-Phe-L-Val	L-phenylalanyl-L-valine
L-Ala-L-Phe	L-alanyl-L-phenylalanine
L-Trp-NH_2	L-tryptophanamide hydrochloride
L-Trp-OMe	L-tryptophan methyl ester hydrochloride
BC	berberine chloride
DI-Water	deionized water
HS	human blood serum
DC	direct current
PMT	photomultiplier tube
HT	high tension voltage
BW	bandwidth
Acc	accumulations
LP-Filter	long-pass filter
D.I.T	digital integration time
$t_{measure}$	measuring time
ΔF corr.	HT voltage corrected ΔF
V	volts
h	hours
λ	wavelength
λ_{obs}	monitoring wavelength
ε	molar extinction coefficient

λ_{exc}	excitation wavelength
λ_{ems}	emission wavelength
FL	fluorescence
T	temperature
S.D	standard deviation
R_f	retention value
M.p.	melting point
NMR	nuclear magnetic resonance
DEPT	distortionless enhancement by polarization transfer
DOSY	diffusion-ordered spectroscopy
IR	infrared radiation
ATR	attenuated total reflection
MS	mass spectrometry
EI	electron ionization
HRMS	high resolution mass spectrometry
HPLC	high performance liquid chromatography
DLS	dynamic light scattering
RI	refractive index
ee	enantiomeric excess

2. Materials

All solvents were used as received from Aldrich or Fluka without further purification. All chemicals were purchased and used as received unless stated otherwise. **CB8** was synthesized according to literature procedures[1] and was also purchased from Strem or Sigma. The MPCP dye enantiomers (R_p)-MPCP and (S_p)-MPCP, and the (S_p)-MVCP dye were synthesized according to the procedures described in section 4 and 5, respectively. MDPP was synthesized from 1,3,8,10-tetrahydro-2,9-dimethyl-2,9-diazadibenzoperylene, according to literature procedures.[2]

3. Instrumentation

Absorption spectra were measured on a Jasco V-730 double-beam UV–VIS spectrophotometer and baseline corrected. Steady-state emission spectra were recorded on a Jasco FP-8300 fluorescence spectrometer equipped with a 150 W xenon arc lamp, single-grating excitation, and emission monochromators. Emission and excitation spectra were corrected for source intensity (lamp and grating) and the emission spectral response (detector and grating) by standard correction curves. ECD, LD, FDCD and FDLD spectra were recorded on a Jasco J-1500 CD spectrometer equipped with a Peltier-thermostated cell holder and an emission optical kit including a collecting lens and a filter holder. The spectrometer contains two PMT detectors: a standard detector and a dedicated FDCD detector, which allows the simultaneous measurement of ECD/LD and FDCD/FDLD signals. Appropriate long pass filters were used for the FDCD/FDLD measurements to avoid the scattered light from excitation wavelength, which could contaminate the observed emission, and to maximize the emitted light signal. The HT voltage applied to the PMT of the ECD detector (standard detector) was kept in auto mode and the HT voltage applied to the PMT of the FDCD detector was kept in manual mode and adjusted accordingly so that a fluorescence signal saturation is not reached in each set of measurements. The applied HT voltage is reported for all experiments. The ECD/LD and FDCD/FDLD spectra reported were baseline corrected for the appropriate solvent system used. As the FDCD measurements were conducted in manual mode (varying DC voltage by fixing HT voltage on PMT of FDCD detector), the instrument initially outputs the fluorescence intensity difference for the two circularly polarized excitations ($F_L - F_R = \Delta F$) which is then corrected for the total fluorescence ($F_L + F_R$ = DC voltage). The FDCD spectrum was hence obtained by dividing the baseline corrected ΔF signal with the baseline corrected DC voltage (= total fluorescence collected from the sample) for each measurement. The ΔF spectra reported were corrected for the signal artefacts if any, arising from the achiral host or dye molecule alone, in cases when the artefacts overlapped with the induced chiroptical bands for the host-guest complexes. Similarly, for the FDLD

measurements conducted in manual mode (varying DC voltage by fixing HT voltage on PMT of FDCD detector), the instrument initially outputs the fluorescence intensity difference for the two linearly polarized excitations ($F_{\parallel} - F_{\perp}$), which is then baseline corrected and divided by the baseline corrected DC voltage (= total fluorescence collected from the sample) to obtain the FDLD spectrum. The LB spectra were measured on a JASCO J-1500 CD spectrometer at a JASCO facility in Pfungstadt by placing a Glan-Taylor polarizer behind the sample at 45° into the beam path. The fluorescence anisotropy spectra were recorded on a JASCO FP-8500 fluorescence spectrometer using FDP-837 automatic excitation and emission polarizers at the JASCO facility in Pfungstadt.

All the spectral measurements were conducted at 25°C unless stated otherwise. For measurements conducted in water, deionized water was used in all cases. Blank measurements of water or buffer provided no induced ECD/LD and FDCD/FDLD effects in the regions examined. For spectroscopy analysis in quartz cuvettes, suprasil (type 111-QS) emission cuvettes with a light path of 10 mm and dimensions of 10x10 mm from Hellma-Analytics were utilized. Dynamic Light Scattering (DLS) experiments were carried out on a Malvern Zetasizer Nano ZS in disposable polystyrene cuvettes with a light path of 10 mm and dimensions of 10x10 mm at 25°C.

The NMR spectra of the compounds described herein were recorded on a Bruker Avance 400 NMR instrument at 400 MHz for ^{1}H NMR and 101 MHz for ^{13}C NMR. The NMR spectra were recorded at room temperature in deuterated solvents acquired from Eurisotop. The chemical shift δ is displayed in parts per million [ppm] and the references used were the ^{1}H and ^{13}C peaks of the solvents themselves. For the characterization of centrosymmetric signals, the signals median point was chosen, for multiplets the signal range. Signals of the ^{13}C spectrum were assigned with the help of distortionless enhancement by polarization transfer spectra DEPT90 and DEPT135. The DOSY NMR spectra was recorded on a Bruker Ascend TM 400 NMR spectrometer at 25°C.

The infrared spectra (IR) were recorded with a Bruker, IFS 88 instrument. Solids were measured by attenuated total reflection (ATR) method. The positions of the respective transmittance bands are given in wave numbers $\tilde{\nu}$ (cm^{-1}). Characterization of the transmittance bands was done in sequence of transmission strength T with following abbreviations: vs (very strong, 0−9% T), s (strong, 10−39% T), m (medium, 40−69% T), w (weak, 70−89% T), vw (very weak, 90−100% T) and br (broad).

Mass Spectrometry (MS): Electron ionization (EI) and fast atom bombardment (FAB) experiments were conducted using a Finnigan, MAT 90 (70 eV) instrument, with 3-nitrobenzyl alcohol (3-NBA) as matrix and reference for high resolution. For the interpretation of the spectra, molecular peaks $[M]^{+}$, peaks of

protonated molecules $[M+H]^+$ and characteristic fragment peaks are indicated with their mass-to-charge ratio (m/z) and in case of EI their intensity in percent, relative to the base peak (100%) is given. In case of high-resolution measurements, the tolerated error is 0.0005 m/z. APCI and ESI experiments were recorded on a Q-Exactive (Orbitrap) mass spectrometer (Thermo Fisher Scientific, San Jose, CA, USA) equipped with a HESI II probe to record high resolution. The tolerated error is 5 ppm of the molecular mass. Again, the spectra were interpreted by molecular peaks $[M]^+$, peaks of protonated molecules $[M+H]^+$ and characteristic fragment peaks and indicated with their mass-to-charge ratio (m/z).

Purification of chiral intermediates were conducted using two preparative HPLC setups:

1) JASCO HPLC System (LC-NetII/ADC) equipped with two PU-2087 Plus pumps, a CO-2060 Plus thermostat, an MD-2010 Plus diode array detector and a CHF-122SC fraction collector of ADVANTEC. For the purification of the (*rac*)-4-bromo[2.2]paracyclophane, a Daicel Chiralpak (AZ-H 30 × 250 mm, particle size of 5 µm) was used with HPLC-grade acetonitrile as mobile phase. Detection was conducted at 218 nm.

2) PuriFlash 4125 by Interchim, equipped with InterSoft V5.1.08, a UV diode array detector (200–600 nm), detection was set to 200 nm and 256 nm. The YMC CHIRAL ART Amylose-SA (10 × 250 mm, particle size of 5 µm) was used as stationary phase. A gradient of HPLC-grade n-hexane/ethyl acetate; 90:10 to 80:20 was used. Analysis of the enantiomeric excess was conducted using an AGILENT HPLC 1100 series system with a G1322A degasser, a G1211A pump, a G1313A autosampler, a G1316A column oven and a G1315B diode array system. Chiralpak AS (4.6 × 250 mm, 5 µm particle size) and Chiralpak OD-H (4.6 × 250 mm, 5 µm particle size) columns were used with HPLC-grade n-hexane/isopropanol as mobile phase, respectively.

4. Synthesis of (S_p)-MPCP (3a) and (R_p)-MPCP (3b)

4.1. Synthesis of (*rac*/S_P/R_P)-4-bromo[2.2]paracyclophane (1, 1a and 1b)

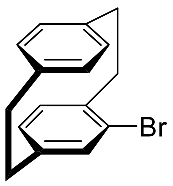

1

A solution of bromine (3.77 mL, 11.8 g, 73.7 mmol, 1.02 equiv.) in 80 mL of dichloromethane was prepared in a dropping funnel and 5 mL of this solution were added to iron filings (10.0 mg, 2 mol%, 1.44 mmol) in a 500 mL three-necked flask and stirred for 1 h at room temperature. Then 250 mL of dichloromethane and [2.2]paracyclophane (15.0 g, 72.0 mmol, 1.00 equiv.) were added and the reaction

mixture was stirred for another 30 min. The remaining bromine solution was added dropwisely over a period of 5 h and the mixture was stirred for 3 days. The reaction was quenched with saturated Na_2SO_3 solution (200 mL) and stirred for 30 min until full discoloration of the mixture occurred. The organic phase was separated, washed with brine (200 mL) and dried over Na_2SO_4. The solvent was removed under reduced pressure to yield 20.2 g of the title compound (**1**, 70.3 mmol, 98%) as a white solid.

R_f = 0.63 (cyclohexane/ethyl acetate; 50:1). – ^{1}H NMR (400 MHz, $CDCl_3$) δ = 7.19 (dd, *J* = 7.9, 2.0 Hz, 1H), 6.58 (dd, *J* = 7.8, 2.0 Hz, 1H), 6.56 – 6.52 (m, 2H), 6.52 – 6.45 (m, 3H), 3.48 (ddd, *J* = 13.0, 10.1, 2.2 Hz, 1H), 3.22 (ddd, *J* = 13.1, 10.1, 6.1 Hz, 1H), 3.17 – 3.02 (m, 4H), 2.98 – 2.78 (m, 2H) ppm. – ^{13}C NMR (101 MHz, $CDCl_3$) δ = 141.73 (C_{quat}), 139.43 (C_{quat}), 139.22 (C_{quat}, 2C), 137.37 (+, $C_{Ar}H$), 135.17 (+, $C_{Ar}H$), 133.43 (+, $C_{Ar}H$), 133.03 (+, $C_{Ar}H$), 132.37 (+, $C_{Ar}H$), 131.59 (+, $C_{Ar}H$), 128.81 (+, $C_{Ar}H$), 127.09 (C_{quat}), 35.98 (–, CH_2), 35.61 (–, CH_2), 34.95 (–, CH_2), 33.61 (–, CH_2) ppm. – IR (ATR) $\tilde{\nu}$ (cm^{-1}) = 2924 (w), 2849 (w), 1585 (w), 1541 (w), 1497 (w), 1475 (w), 1431 (w), 1408 (w), 1390 (w), 1186 (w), 1092 (vw), 1034 (m), 941 (w), 869 (w), 839 (m), 793 (w), 708 (m), 668 (w), 640 (m), 576 (w), 514 (m), 473 (w), 404 (vw), 382 (w) cm^{-1}. – MS (EI, 70 eV) *m/z* (%) = 288 (27) $[M(^{81}Br)]^+$, 286 (28) $[M(^{79}Br)]^+$, 184 (17) $[C_8H_7{}^{81}Br]^+$, 182 (17) $[C_8H_7{}^{79}Br]$, 104 (100) $[C_8H_8]^+$. – HRMS (EI, $C_{16}H_{15}{}^{79}Br$) calc. 286.0352; found 286.0350. The analytical data is consistent with literature.[3]

Chiral preparative HPLC separation (Figure S 1) of the 4-bromo[2.2]paracyclophane racemate was conducted on a JASCO HPLC system (LC-NetII/ADC) equipped with two PU-2087 puls pumps, a CO-2060 plus thermostat, a MD-2010 plus diode array detector and a CHF-122SC fraction collector from ADVANTEC. Absorption was detected at 200, 218 and 256 nm. As a stationary phase, a CHIRALPAK AZ-H (Daicel Corporation) column (5 µm, 30 mm × 250 mm) was used with acetonitrile as eluent, following group procedure.[4]

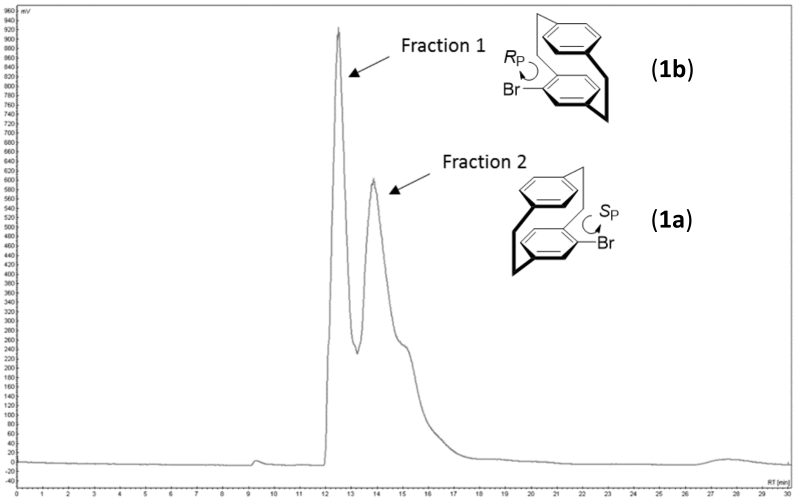

Figure S 1: Chiral preparative HPLC run of 4-bromo [2.2]paracyclophane (**1**).

The absolute configuration was determined by comparison of the electronic circular dichroism (ECD) spectra (Figure S 2) with literature-known experimental and calculated data,[5]as well as with theory.[6]

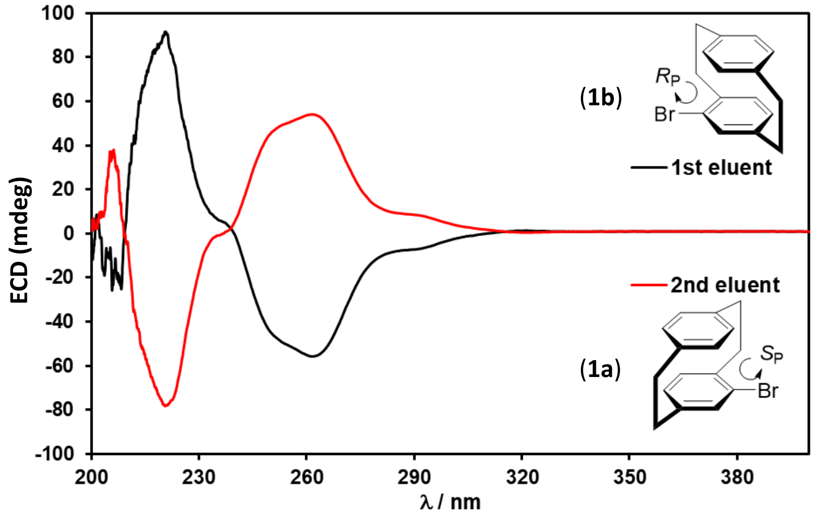

Figure S 2: ECD measurements in acetonitrile of 4-bromo[2.2]paracyclophane (**1a** and **1b**)

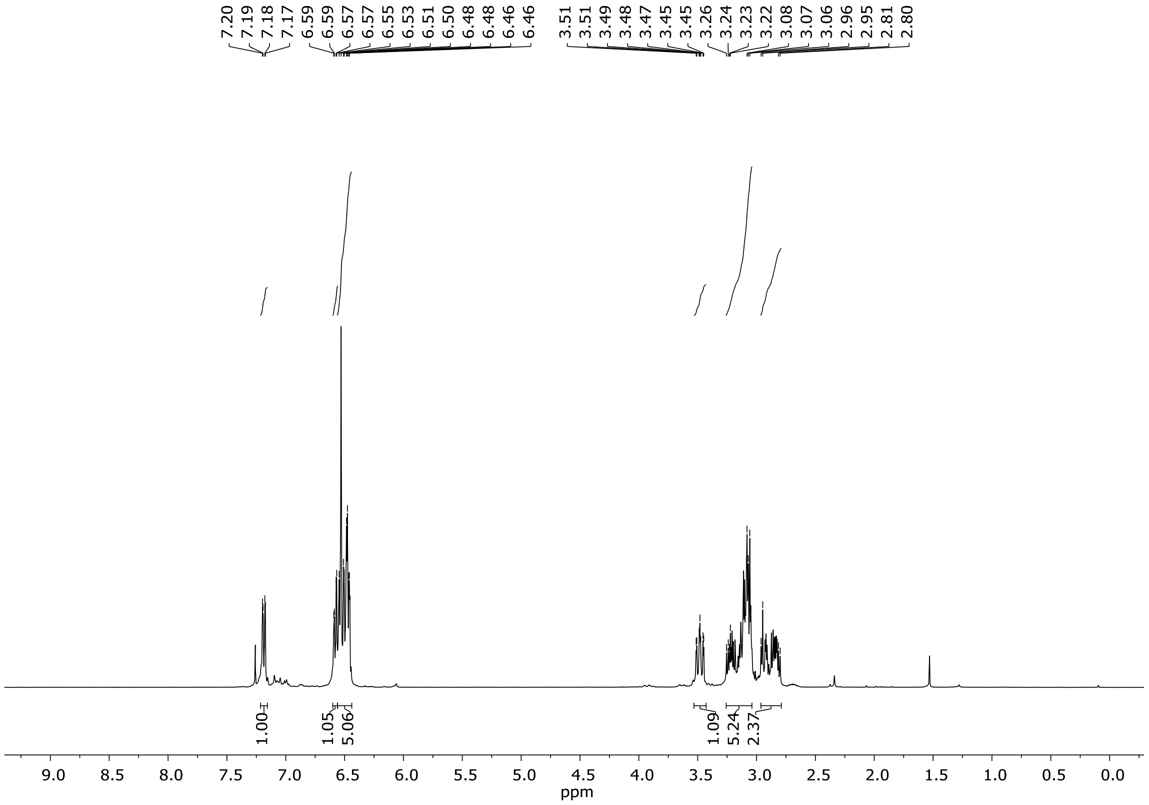

Figure S 3: ^{1}H NMR (400 MHz, $CDCl_3$) spectrum of (*rac*)-4-bromo[2.2]paracyclophane (**1**)

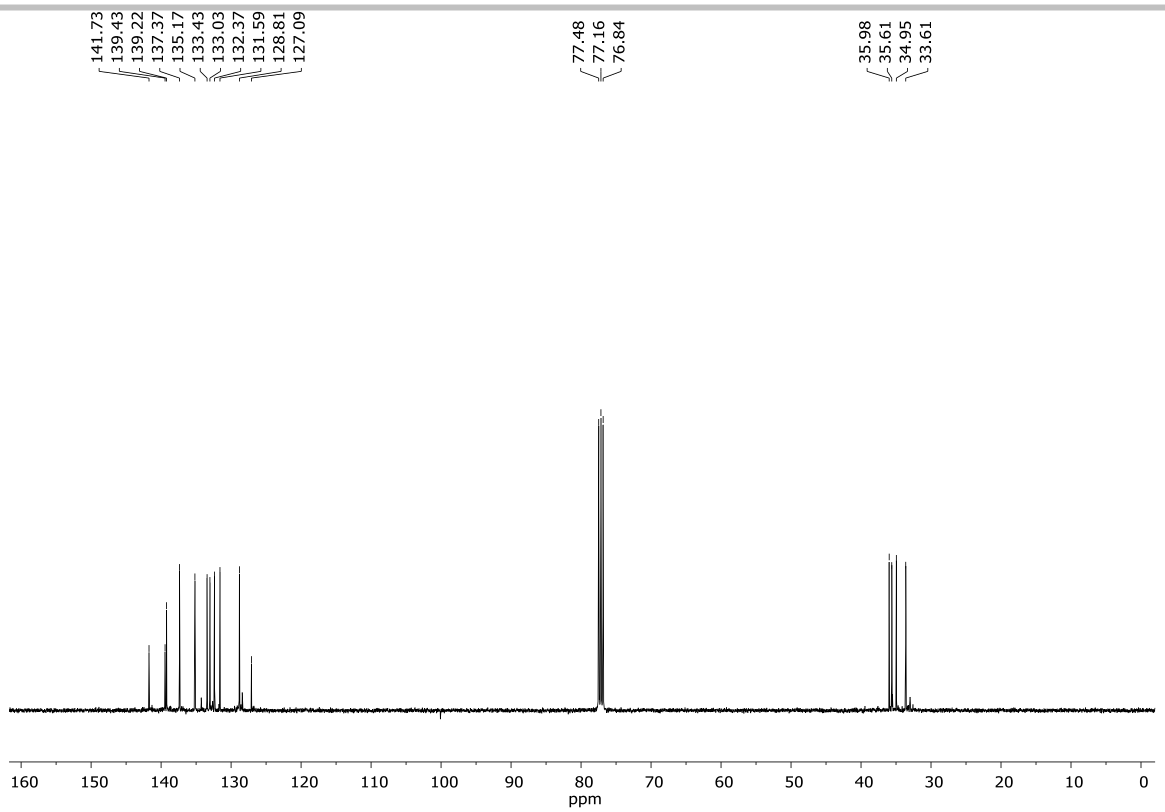

Figure S 4: ^{13}C NMR (101 MHz, $CDCl_3$) spectrum of (*rac*)-4-bromo[2.2]paracyclophane (**1**)

4.2. Synthesis of (S_P/R_P)-4-(4'-pyridyl)[2.2]paracyclophane (2a and 2b)

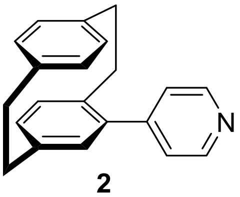

2

A sealable vial was charged with (S_P/R_P)-4-bromo[2.2]paracyclophane (**1a/1b**, 200 mg, 0.70 mmol, 1.00 equiv.), 4-pyridylboronic acid (170 mg, 1.40 mmol, 2.00 equiv.), K_3PO_4 (220 mg, 1.05 mmol, 1.50 equiv.) and $Pd(PPh_3)_4$ (50.0 mg, 0.04 mmol, 6.0 mol%). The sealed vial was evacuated and purged with argon three times. Then 3 mL of dioxane/water (2:1) were added *via* the septum and the reaction mixture was heated to 110°C and stirred for 24 h. It was diluted with water (20 mL) and extracted with ethyl acetate (3 × 25 mL). The combined organic layers were dried over Na_2SO_4, filtered and the solvent was removed under reduced pressure. The residue war purified by column chromatography (silica, cyclohexane/ethyl acetate 2:1) to give 45.1 mg of the title compound (**2a/2b**, 158 mg, 79%) as white solid.

R_f = 0.43 (cyclohexane/ethyl acetate; 2:1). – M.p. 115–118°C. – ^{1}H NMR (400 MHz, $CDCl_3$) δ = 8.70 (d, J = 4.8 Hz, 2H), 7.40 (d, J = 4.8 Hz, 2H), 6.65–6.62 (m, 2H), 6.59–6.56 (m, 3H), 6.54 (s, 2H), 3.40 (ddd, J = 12.5, 10.1, 2.9 Hz, 1H), 3.21–3.12 (m, 3H), 3.09–2.87 (m, 3H), 2.66 (ddd, J = 13.0, 10.0, 4.5 Hz, 1H) ppm. – ^{13}C NMR (101 MHz, $CDCl_3$) δ = 150.2 (+, $C_{Ar}H$), 148.7 (C_{quat}), 140.3 (C_{quat}), 139.7 (C_{quat}, 2C), 139.3 (C_{quat}), 137.4 (C_{quat}), 136.3 (+, $C_{Ar}H$), 133.6 (+, $C_{Ar}H$), 133.4 (+, $C_{Ar}H$), 132.9 (+, $C_{Ar}H$), 132.2 (+, $C_{Ar}H$), 132.1

(+, $C_{Ar}H$), 129.9 (+, $C_{Ar}H$), 124.6 (+, $C_{Ar}H$), 35.6 (–, CH_2), 35.4 (–, CH_2), 35.1 (–, CH_2), 34.1 (–, CH_2) ppm. – IR (ATR) $\tilde{\nu}$ (cm^{-1}) = 2925 (w), 2850 (w), 1895 (w), 1594 (w), 1497 (w), 1474 (vw), 1400 (w), 1211 (vw), 1093 (w), 1066 (vw), 991 (w), 939 (vw), 905 (w), 850 (w), 825 (w), 716 (w), 667 (vw), 646 (w), 622 (w), 594 (w), 563 (w), 515 (w), 483 (w), 423 (vw) cm^{-1}. – MS (EI, 70 eV) *m/z* (%) = 286 (25) $[M+H]^+$, 285 (100) $[M]^+$, 181 (62) $[M–C_8H_7]$+, 180 (100) $[M–C_8H_8]^+$, 105 (51) $[C_8H_9]^+$, 104 (61) $[C_8H_8]^+$. – HRMS (EI, $C_{21}H_{19}N$) calc. 285.1512, found 285.1511. The analytical data is consistent with literature.[3]

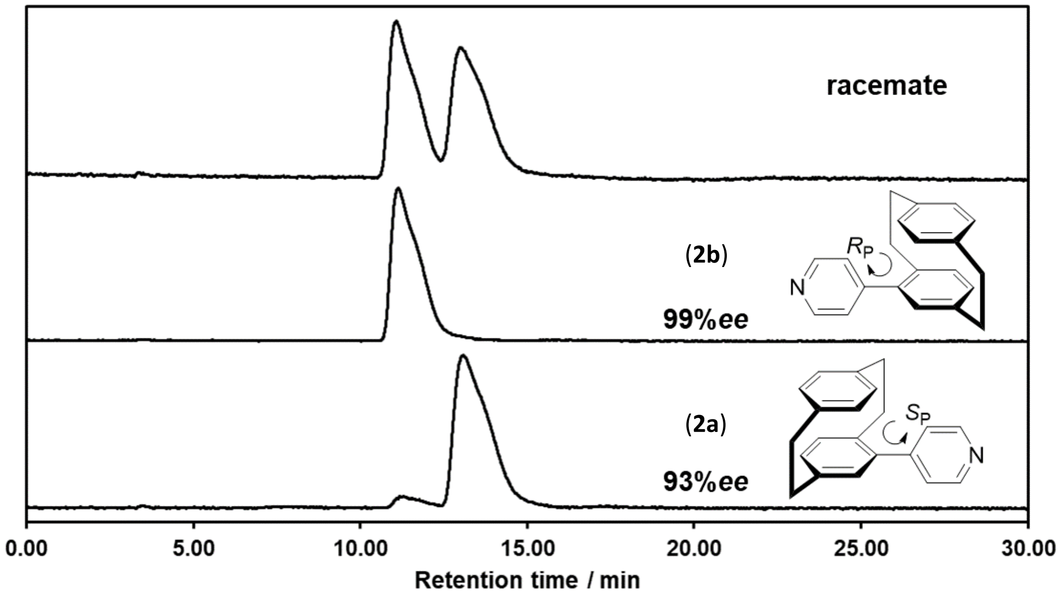

Figure S 5: Analytical HPLC run of enantioenriched 4-pyridyl[2.2]paracyclophanes on a OD-H column using 2% isopropanol in *n*-hexane with a flow rate of 1 mL/min. Detection was performed at 330 nm. All *%ee* values were measured via integration of each enantiomeric peak.

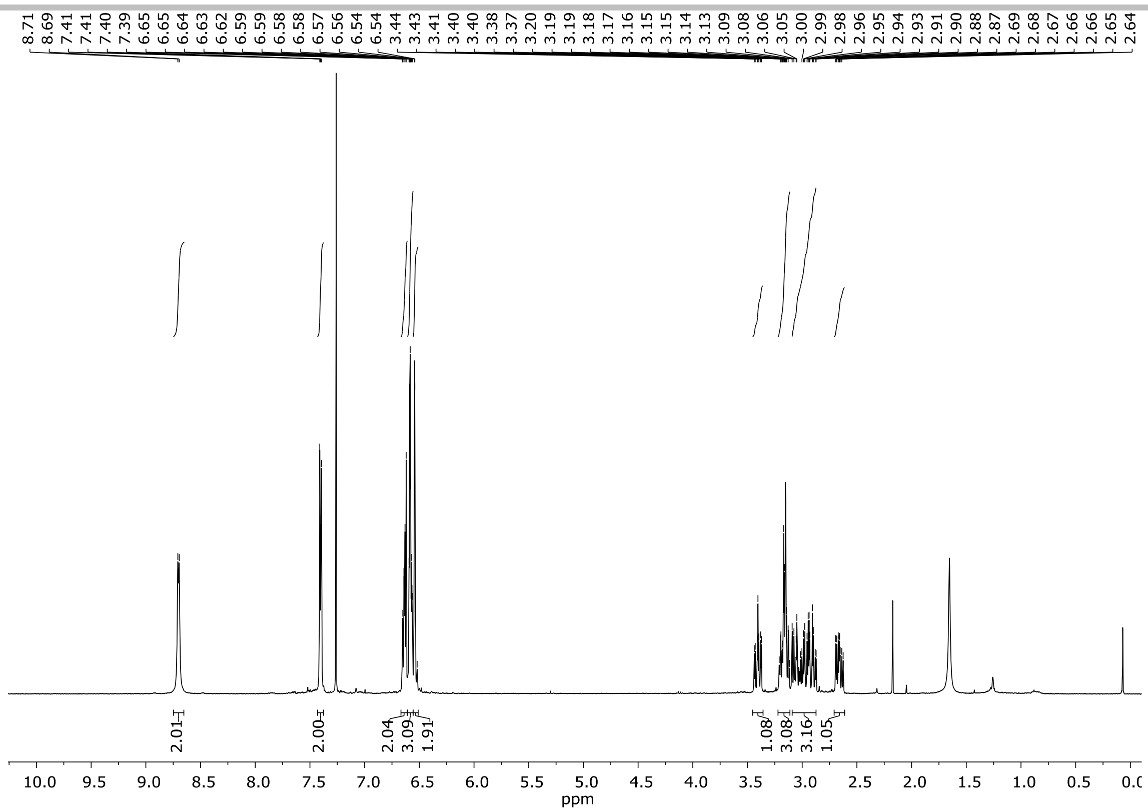

Figure S 6: ^{1}H NMR (400 MHz, $CDCl_3$) spectrum of 4-pyridyl[2.2]paracyclophane (**2**).

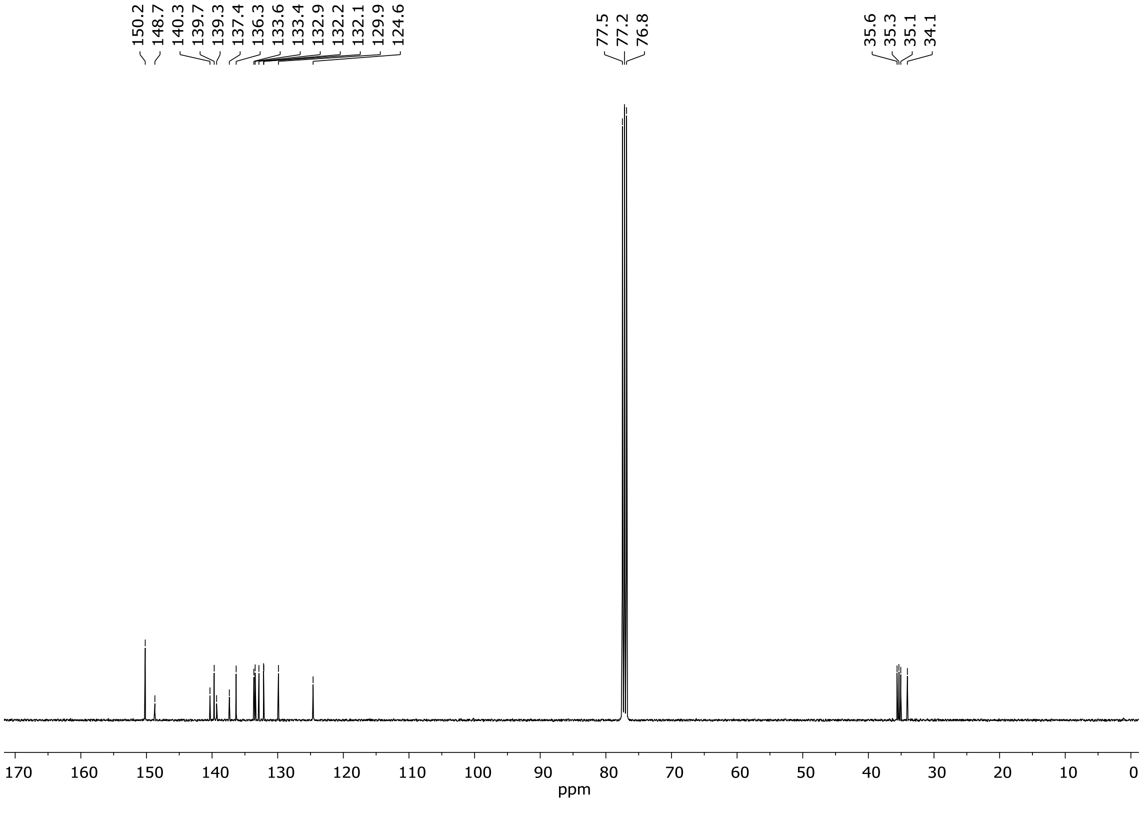

Figure S 7: ^{13}C NMR (101 MHz, $CDCl_3$) spectrum of 4-pyridyl[2.2]paracyclophane (**2**).

4.3. Synthesis of (*S*P/*R*P)-N-methyl-4-pyridylium[2.2]paracyclophane iodide ((*S*p)-MPCP (3a) and (*R*p)-MPCP (3b))

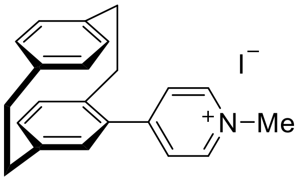

3

In a round-bottom flask, (*S*P/*R*P)-4-(4'-pyridyl)[2.2]paracyclophane (**2a/2b**, 50.0 mg, 180 µmol, 1.00 equiv.) was dissolved in 1.5 mL DCM. Methyl iodide (1.00 mL, 2.28 g, 16.1 mmol, 894 equiv.) was added at room temperature resulting in a yellow solution. The crude mixture was allowed to react for 3 days, while two portions of MeI (1 mL) were additionally added after each day. The reaction mixture was extracted three times with water. After the removal of water by lyophilization, 42.1 mg of the title compound (**3a/3b**, 98.5 µmol, 55%) was obtained as a yellow powder.

^{1}H NMR (500 MHz, D_2O) δ = 8.76 (d, *J* = 6.4, 2H), 8.14 (d, *J* = 6.5, 2H), 6.86 (d, *J* = 11.5, 3H), 6.80 – 6.68 (m, 3H), 6.53 (d, *J* = 7.9, 1H), 4.38 (s, 3H), 3.45 – 3.35 (m, 1H), 3.25 – 3.06 (m, 5H), 3.03 – 2.94 (m, 1H), 2.62 – 2.52 (m, 1H) ppm. – ^{13}C NMR (126 MHz, D_2O) δ = 144.6 (+, CArH), 141.6 (C_{quat}), 140.6 (C_{quat}), 140.0 (C_{quat}), 138.8 (C_{quat}), 137.0 (+, CArH), 135.9 (+, CArH), 135.8 (C_{quat}), 133.7 (+, CArH), 133.2 (+, CArH), 132.4 (+, CArH), 131.8 (+, CArH), 129.5 (+, CArH), 127.7 (+, CArH), 47.2 (+, CH_3), 34.6 (–, CH_2), 34.3 (–, CH_2), 34.2 (–, CH_2), 33.4 (–, CH_2) ppm. – IR (ATR) $\tilde{\nu}$ (cm^{-1}) = 3386 (vw), 3032 (vw), 2920 (vw), 2850 (vw), 2573, (vw), 2322 (vw), 2168 (vw), 2020 (vw), 1918 (vw), 1639 (vw), 1516 (vw), 1454 (vw), 1332 (vw), 1198 (vw), 1096 (vw), 949 (vw), 843 (w), 720 (vw), 642 (vw), 488 (vw) cm^{-1}. – HRMS (ESI, $C_{22}H_{22}N^{+}$) calc. 300.1747, found 300.1737. The analytical data is consistent with literature.[7]

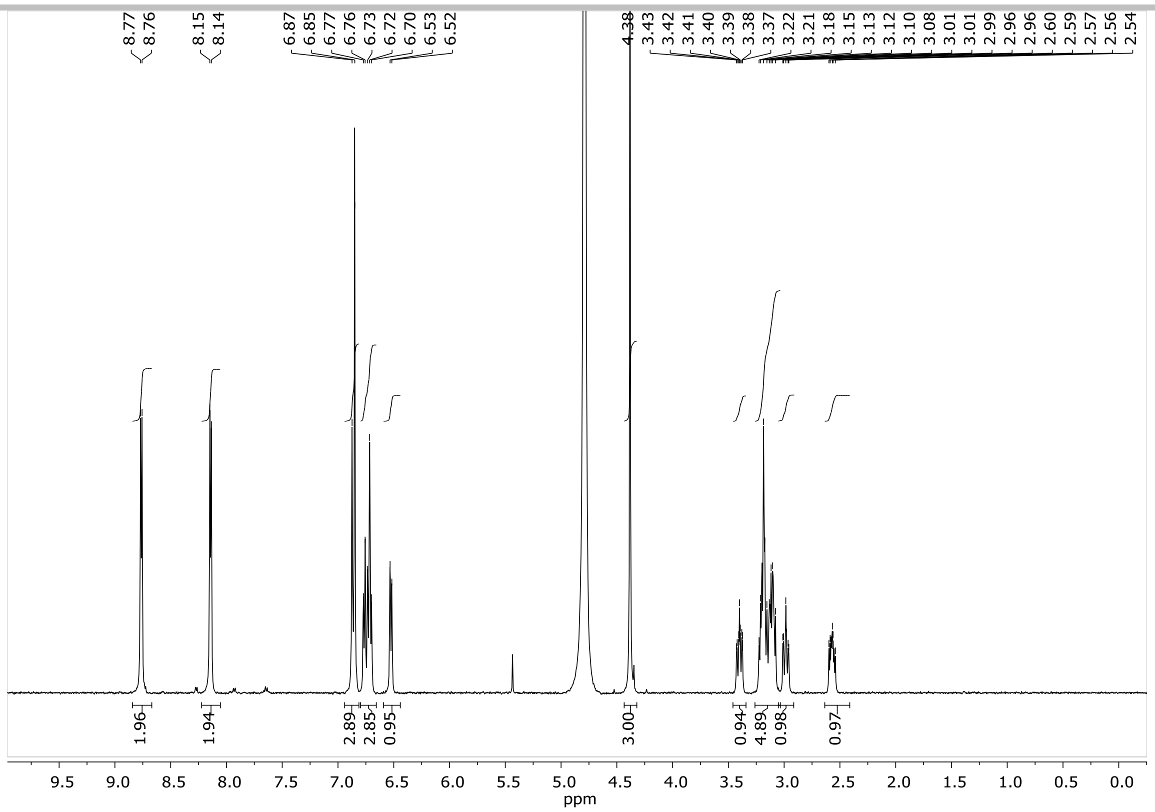

Figure S 8: ^{1}H NMR (500 MHz, D_2O) spectrum of *N*-methyl-4-pyridinylium[2.2]paracyclophane (**3**, **MPCP**)

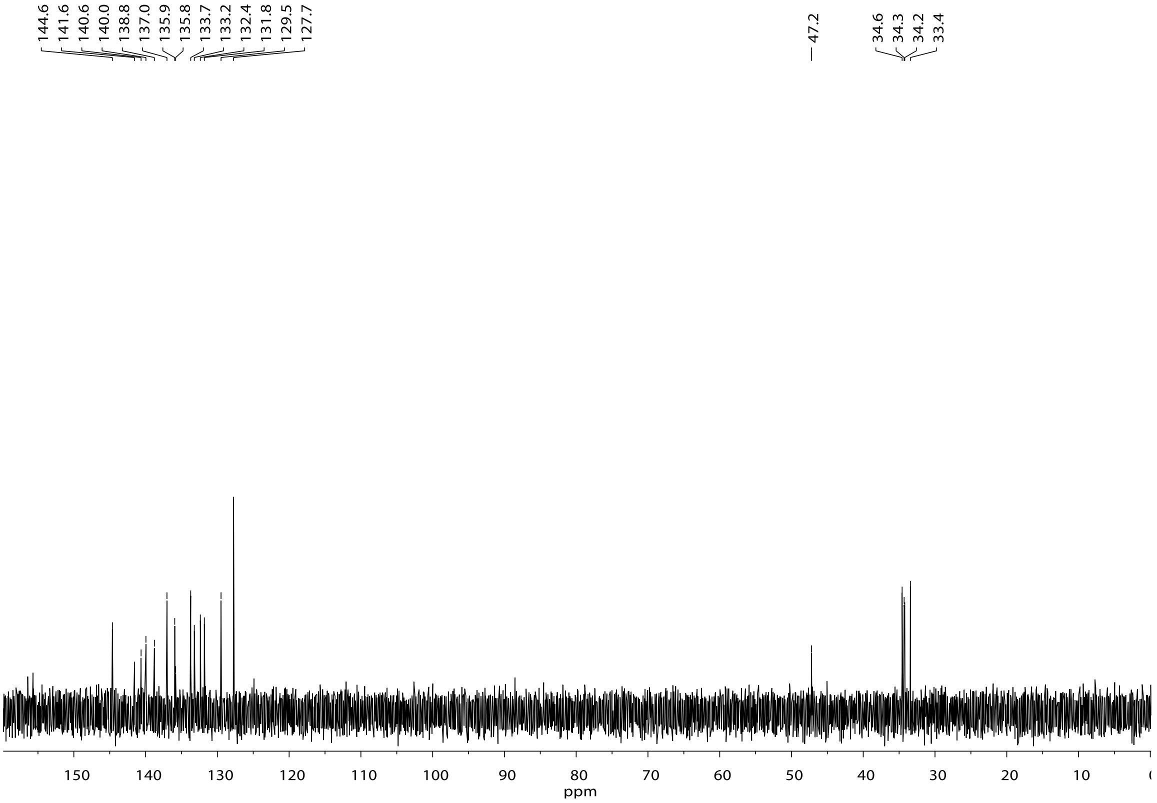

Figure S 9: ^{13}C NMR (126 MHz, D_2O) spectrum of *N*-methyl-4-pyridinylium[2.2]paracyclophane (**3**, **MPCP**).

5. Synthesis of (S_p)-MVCP (6a)

5.1. Synthesis of (*rac*)-4-formyl[2.2]paracyclophane (4)

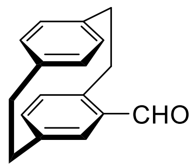

4

In argon environment, [2.2]paracyclophane (12.0 g, 58 mmol) was dissolved in CH_2Cl_2 (300 mL) and cooled to 0°C. Titanium(IV)chloride (12.63 mL, 115 mmol) and dichloromethoxymethane (5.5 mL, 60.5 mmol) were added subsequently. The mixture was stirred at 0°C to room temperature for 6 h, poured into ice water (200 mL), and stirred for another 1 h. The two phases were separated, and the aqueous phase was extracted with CH_2Cl_2 (100 mL). The combined organic phases were dried over $MgSO_4$ and concentrated under reduced pressure. The crude product was purified by column chromatography on silica (cyclohexane/ ethyl acetate 20:1) followed by recrystallization in 200 mL hot cyclohexane to give 12.8 g (*rac*)-4-formyl[2.2]paracyclophane (**4**, 54.1 mmol, 94%) as white solid.

R_f = 0.42 (cyclohexane/ethyl acetate 10:1). – ^1H NMR (400 MHz, $CDCl_3$) δ = 9.95 (s, 1H, O=CH), 7.02 (d, J = 2.0 Hz, 1H, H^{Ar}), 6.73 (dd, J = 7.8, 2.0 Hz, 1H, H^{Ar}), 6.59 (d, J = 7.8 Hz, 1H. H^{Ar}), 6.56 (d, J = 1.9 Hz, 1H, H^{Ar}), 6.50 (dd, J = 7.8, 1.8 Hz, 1H, H^{Ar}), 6.43 (dd, J = 7.9, 1.9 Hz, 1H, H^{Ar}), 6.38 (dd, J = 7.9, 1.9 Hz, 1H, H^{Ar}), 4.11 (ddd, J = 13.1, 9.9, 1.8 Hz, 1H, H^{PC}), 3.28 – 3.15 (m, 3H, H^{PC}), 3.13 – 3.02 (m, 3H, H^{PC}), 2.95 (ddd, J = 13.1, 10.2, 6.7 Hz, 1H, H^{PC}). – ^{13}C NMR (101 MHz, $CDCl_3$) δ = 192.1 (+, CH, C=O), 143.4 ($C_{quat.}$, C^{Ar}), 140.8 ($C_{quat.}$, C^{Ar}), 139.6 ($C_{quat.}$, C^{Ar}), 139.6 ($C_{quat.}$, C^{Ar}), 138.2 (+, CH, C^{Ar}), 136.7 ($C_{quat.}$, C^{Ar}), 136.5 (+, CH, C^{Ar}), 136.2 (+, CH, C^{Ar}), 133.4 (+, CH, C^{Ar}), 133.0 (+, CH, C^{Ar}), 132.5 (+, CH, C^{Ar}), 132.3 (+, CH, C^{Ar}), 35.4 (–, CH_2), 35.3 (–, CH_2), 35.1 (–, CH_2), 33.7 (–, CH_2). – IR (ATR) $\tilde{\nu}$ (cm^{-1}) = 2924 (w), 2850 (w), 1677 (m), 1588 (w), 1554 (w), 1497 (w), 1436 (w), 1409 (w), 1282 (w), 1226 (w), 1180 (w), 1115 (w), 906 (w), 874 (w), 795 (w), 772 (w), 658 (w), 635 (m), 623 (m), 516 (m) cm^{-1}. – MS (EI, 70 eV), m/z (%) = 236 (76) $[M]^+$, 104 (100) $[C_8H_8]^+$. – HRMS ($C_{17}H_{16}O$) calc. 236.1201; found 236.1201.

The analytical data and spectral properties are consistent with literature.[8]

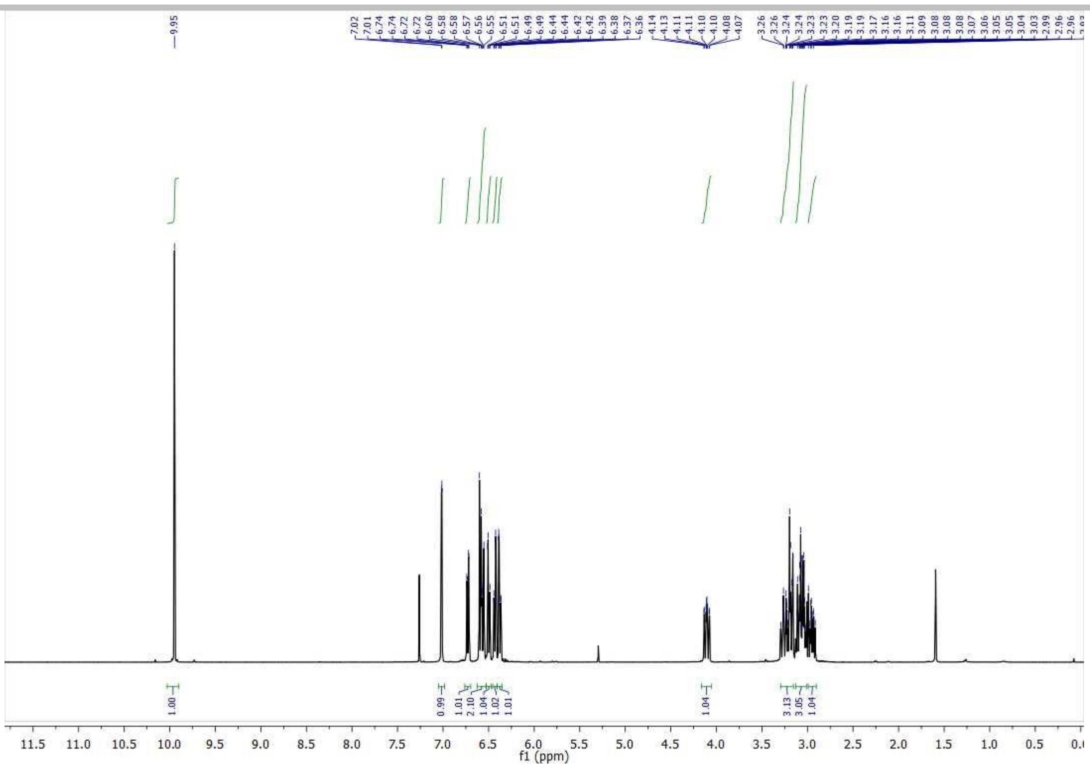

Figure S 10: ^{1}H NMR (400 MHz, $CDCl_3$) spectrum of (*rac*)-4-formyl[2.2]paracyclophane (**4**)

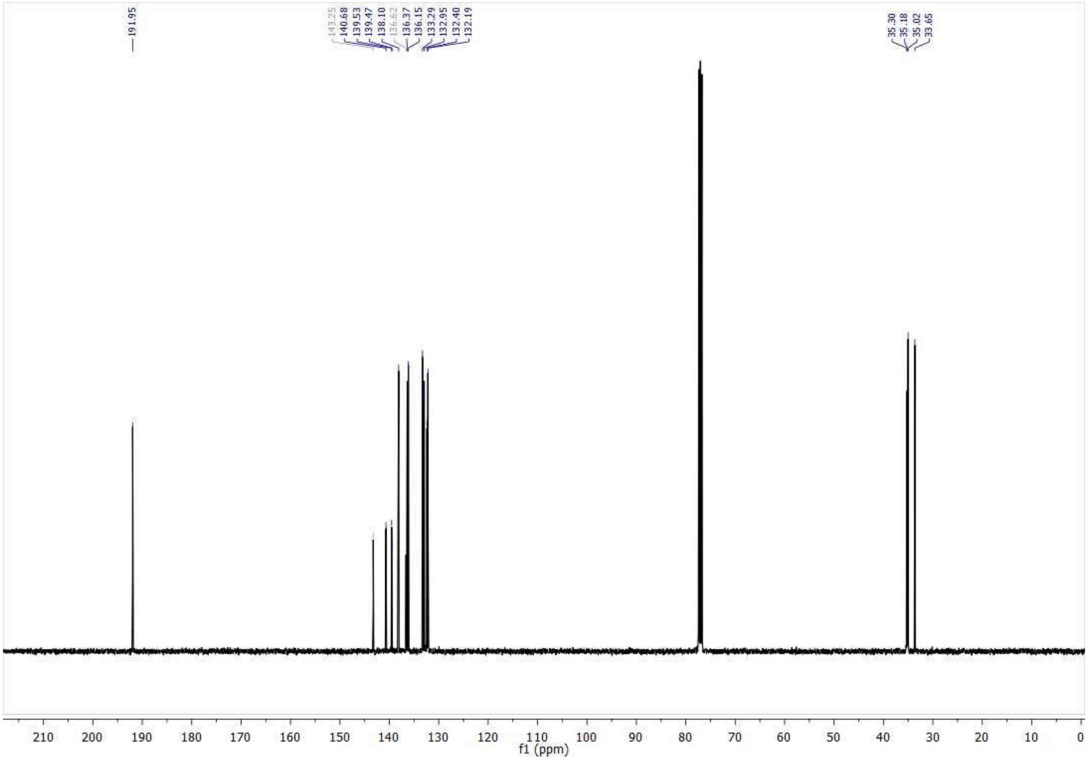

Figure S 11: ^{13}C NMR (101 MHz, $CDCl_3$) spectrum of (*rac*)-4-formyl[2.2]paracyclophane (**4**)

5.2. Synthesis of (S_P,*R*)-[N-1-(phenylethyl)]-4-[2.2]paracyclophanyl methanimine (5a)

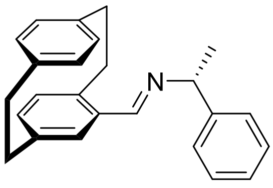

(**5a**, S_p,*R*)

A solution of racemic 4-formyl[2.2]paracyclophane (23.5 g, 99 mmol) and *R*-(+)-phenylethylamine (12.1 g, 12.66 mmol) in 300 mL of toluene was refluxed under magnetic stirring. The course of the reaction was followed by ^{1}H NMR because the reaction product proved to be unstable under, either SiO_2 or thin layer chromatography (TLC). After 5 h the reaction was completed, the solvent was evaporated in vacuo and the crude product was crystallized from 250 mL of *n*-hexane affording a white solid. After filtration the solid was recrystallized from 150 mL of *n*-hexane. Hence the residue was recrystallized twice out of hot hexane to obtain 13.16 g (S_P,*R*)-[N-1-(phenylethyl)]-4-[2.2]paracyclophanyl methanimine (**5a,** 26.7 mmol, 27%) as a light-yellow solid determined by ^{1}H NMR spectroscopy.

^{1}H NMR (400 MHz, $CDCl_3$) δ 8.37 (s, 1H, N=C*H*), 7.56 – 7.49 (m, 2H, 2H, H^{Ph}), 7.40 (dd, J = 8.5, 6.9 Hz, 2H, H^{Ph}), 7.31 – 7.27 (m, 1H, H^{Ph}), 7.00 (d, J = 2.0 Hz, 1H, H^{Ar}), 6.58 (dd, J = 7.8, 2.0 Hz, 1H, H^{Ar}), 6.53 – 6.46 (m, 3H, H^{Ar}), 6.38 (dt, J = 7.9, 1.1 Hz, 1H, H^{Ar}), 6.29 (dt, J = 7.7, 1.1 Hz, 1H, H^{Ar}), 4.57 (q, J = 6.6 Hz, 1H, N-C*H*PhCH3), 3.98 – 3.83 (m, 1H, H^{PC}), 3.23 – 2.98 (m, 5H, H^{PC}), 2.93 – 2.80 (m, 2H, H^{PC}), 1.66 (d, J = 6.6 Hz, 3H, C*H*3). ^{13}C NMR (101 MHz, $CDCl_3$) δ 158.59 (+, CH, C=N), 145.70 (Cquat., C^{Ar}), 140.46 (Cquat., C^{Ar}), 140.05(Cquat., C^{Ar}), 139.42(Cquat., C^{Ar}), 139.35(Cquat., C^{Ar}), 136.05(Cquat., C^{Ar}), 135.32(+, CH, C^{Ar}), 134.31(+, CH, C^{Ar}), 133.57(+, CH, C^{Ar}), 133.21(+, CH, C^{Ar}), 132.89(+, CH, C^{Ar}), 131.99(+, CH, C^{Ar}), 131.13(+, CH, C^{Ar}), 128.47(+, CH, 2C, C^{Ph}), 126.82(+, CH, C^{Ph}), 126.57(+, CH, 2C, C^{Ph}), 70.35 (+, CH, N-*C*HPhCH3), 35.35 (–, CH2), 35.10 (–, CH2), 34.99 (–, CH2), 33.94 (–, CH2), 25.02 (+, CH3). – IR (ATR) $\tilde{\nu}$ (cm^{-1}) = 2955 (s), 2919 (vs), 2869 (m), 2850 (vs), 1641 (w), 1461 (w), 1457 (w), 1377 (m), 1228 (w), 1206 (w), 1183 (w), 1164 (w), 1156 (w), 1121 (w), 1114 (w), 1103 (w), 1091 (w), 1077 (w), 1067 (w), 1032 (w), 1018 (w), 971 (w), 719 (w), 700 (w) cm^{-1}. – MS (EI, 70 eV), m/z (%) = 340.2 (100) [M+H]$^+$. – HRMS ($C_{25}H_{26}N^+$) calc. 340.2060; found 340.2075.

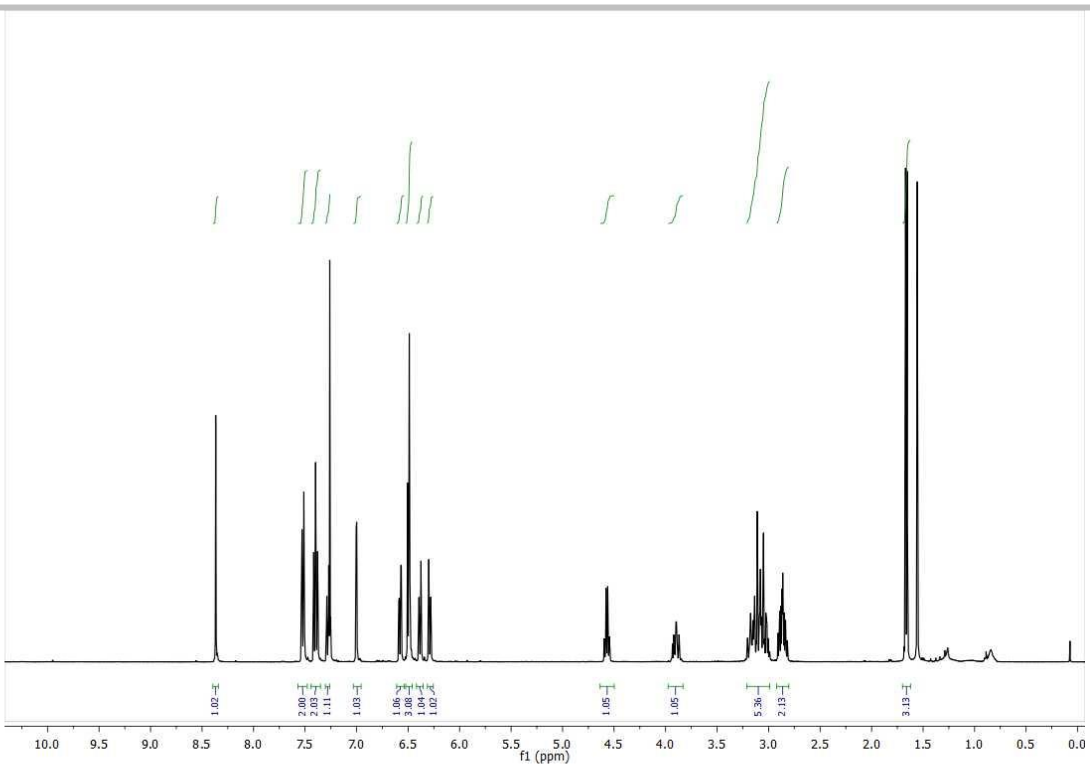

Figure S 12: ^{1}H NMR (400 MHz, $CDCl_3$) spectrum of (S_P,*R*)-[N-(1-(phenylethyl)]-4-[2.2]paracyclophanyl methanimine (**5a**)

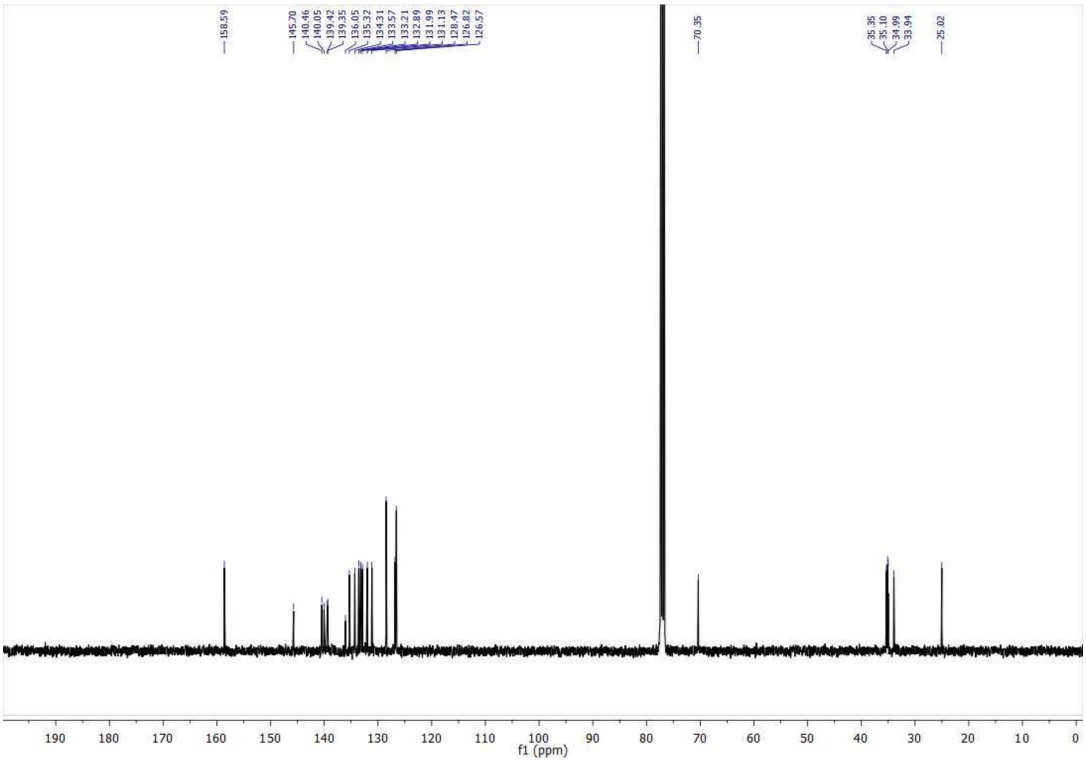

Figure S 13: ^{13}C NMR (101 MHz, $CDCl_3$) spectrum of (S_P,*R*)-[N-(1-(phenylethyl)]-4-[2.2]paracyclophanyl methanimine (**5a**)

5.3. Synthesis of (S_P)-4-formyl[2.2]paracyclophane (4a)

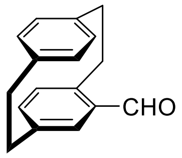

(**4a**, S_p)

13.16 g (S_p, R)-[N-1-(Phenylethyl)]-4-[2.2]paracyclophanyl methanimine (**5a**, 38.8 mmol) were hydrolyzed by column chromatography on silica using dichloromethane as eluent to get 8.98 g (S_p)-4-formyl[2.2]paracyclophane (**4a**) as white solid with 98%*ee*.

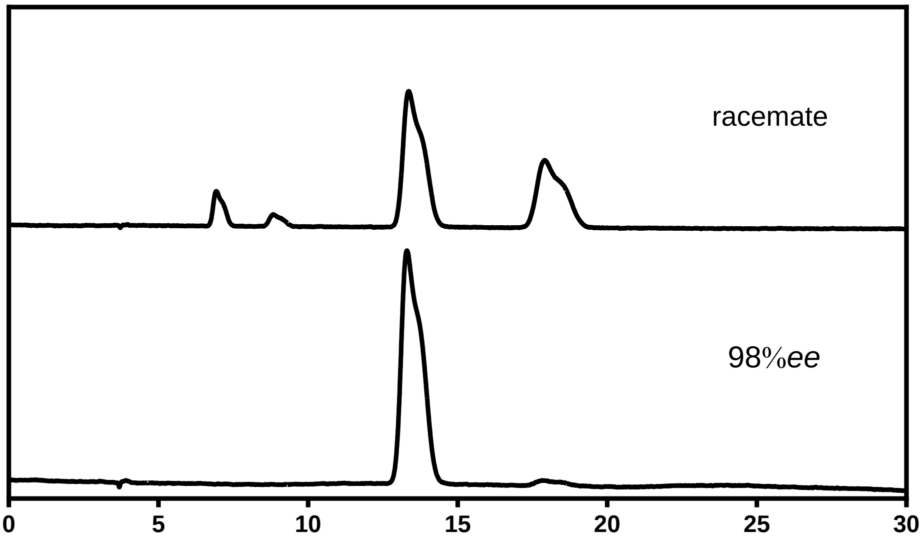

Figure S 14: Analytical HPLC run of 4-formyl[2.2]paracyclophane on a chiral OD-H column using 1% isopropanol in *n*-hexane with a flow rate of 1 mL/min. Detection was performed at 330 nm. The *%ee* values were measured via integration of enantiomeric peak.

The enantiopure compound shows the same analytical data as reported for the racemic mixture.[8]

5.4. Synthesis of (E)-4-((S_P)-4-vinyl[2.2]paracyclophane)-1-methyl pyridin-1-ium iodide((S_p)-MVCP (6a))

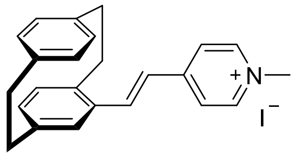

6a, (S_p)-MVCP

1,4-Dimethylpyridinium iodide (438 mg, 0.55 mmol) was followed by (S_p)-4-formyl[2.2]paracyclophane (**4a**, 400 mg, 0.5 mmol), methanol (10 mL) and 3 drops of piperidine. The reaction was stirred and heated at 65^{0}C for 2 h. The reaction mixture was allowed to reach room temperature at which point most compounds precipitated. Acetic acid was added to those reactions involving aldehydes with hydroxyl groups. In some cases, the addition of ethyl acetate was necessary to induce precipitation. The crude product was purified by column chromatography on silica (dichloromethane/ methane 5:1) to give

168 mg (*E*)-4-((*S*$_P$)-4-vinyl[2.2]paracyclophane)-1-methyl pyridin-1-ium iodide ((*S*$_p$)-MVCP) **6a**, 0.37 mmol, 22%) as brown solid.

R$_f$ = 0.5 (dichloromethane/methane 5:1). –^{1}H NMR (400 MHz, DMSO-d$_6$) δ = 8.87 (d, J = 6.5 Hz, 2H, H^{PY}), 8.38 (d, J = 6.6 Hz, 2H, H^{PY}), 7.96 (d, J = 16.1 Hz, 1H, CH=CH), 7.30 (d, J = 16.1 Hz, 1H, CH=CH), 6.96 (d, J = 1.7 Hz, 1H, H^{Ar}), 6.63 (dd, J = 7.8, 1.7 Hz, 1H, H^{Ar}), 6.59 – 6.52 (m, 3H, H^{Ar}), 6.45 (q, J = 1.1 Hz, 2H, H^{Ar}), 4.28 (s, 3H, CH_3), 3.84 – 3.72 (m, 1H, H^{PC}), 3.13 – 3.07 (m, 3H, H^{PC}), 3.04 – 2.97 (m, 3H, H^{PC}), 2.93 – 2.88 (m, 1H, H^{PC}). –^{13}C NMR (101 MHz, DMSO-d$_6$) δ 153.28(C$_{quat.}$, C^{Ar}), 145.37(+, 2C, CH, C^{Py}), 141.51(C$_{quat.}$, C^{Ar}), 140.67(C$_{quat.}$, C^{Ar}), 139.55(C$_{quat.}$, C^{Ar}), 139.38(C$_{quat.}$, C^{Ar}), 138.85(+, CH, C=C), 135.83(C$_{quat.}$, C^{Ar}), 135.66(+, CH, C^{Ar}), 134.98(+, CH, C^{Ar}), 133.53(+, CH, C^{Ar}), 133.43(+, CH, C^{Ar}), 132.14(+, CH, C^{Ar}), 131.37(+, CH, C^{Ar}), 130.67(+, CH, C^{Ar}), 124.12(+, 2C, CH, C^{Py}), 123.89(+, CH, C=C), 47.32(+, CH_3), 35.27(-, CH_2), 35.18(-, CH_2,), 34.98(-, CH_2), 33.61(-, CH_2). – IR (ATR) $\tilde{\nu}$ (cm^{-1}) = 3009 (m), 2922 (m), 2871 (w), 2850 (m), 1639 (s), 1612 (vs), 1587 (s), 1564 (m), 1514 (s), 1500 (m), 1480 (m), 1468 (m), 1449 (m), 1319 (m), 1208 (w), 1183 (vs), 1154 (s), 1143 (s), 1118 (s), 1101 (s), 1082 (s), 1050 (m), 963 (s), 936 (m), 901 (w), 885 (m), 868 (s), 819 (vs), 798 (s), 728 (m), 717 (s), 642 (s), 615 (m), 582 (m), 520 (s), 506 (vs), 489 (s), 470 (m), 465 (m), 453 (m), 446 (m), 428 (m), 419 (m), 401 (m), 392 (w), 384 (m), 375 (m) cm^{-1}– MS (EI, 70 eV), m/z (%) = 326.3 (100) [M-I]$^+$. – HRMS ($C_{24}H_{24}N^+$) calc.326.1903; found 326.1909.

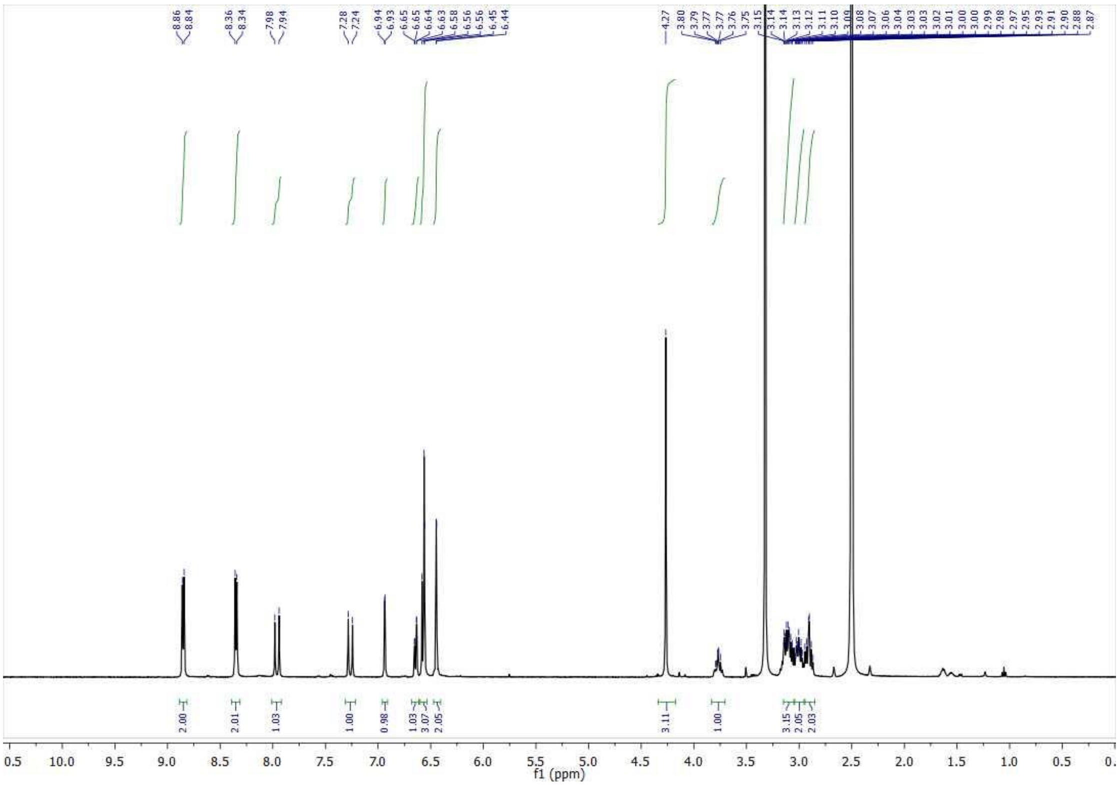

Figure S 15: ^{1}H NMR (400 MHz, DMSO-d$_6$) spectrum of (*E*)-4-((*S*$_P$)-4-vinyl[2.2]paracyclophane)-1-methyl pyridin-1-ium iodide (**6a**, (*S*$_p$)-MVCP)

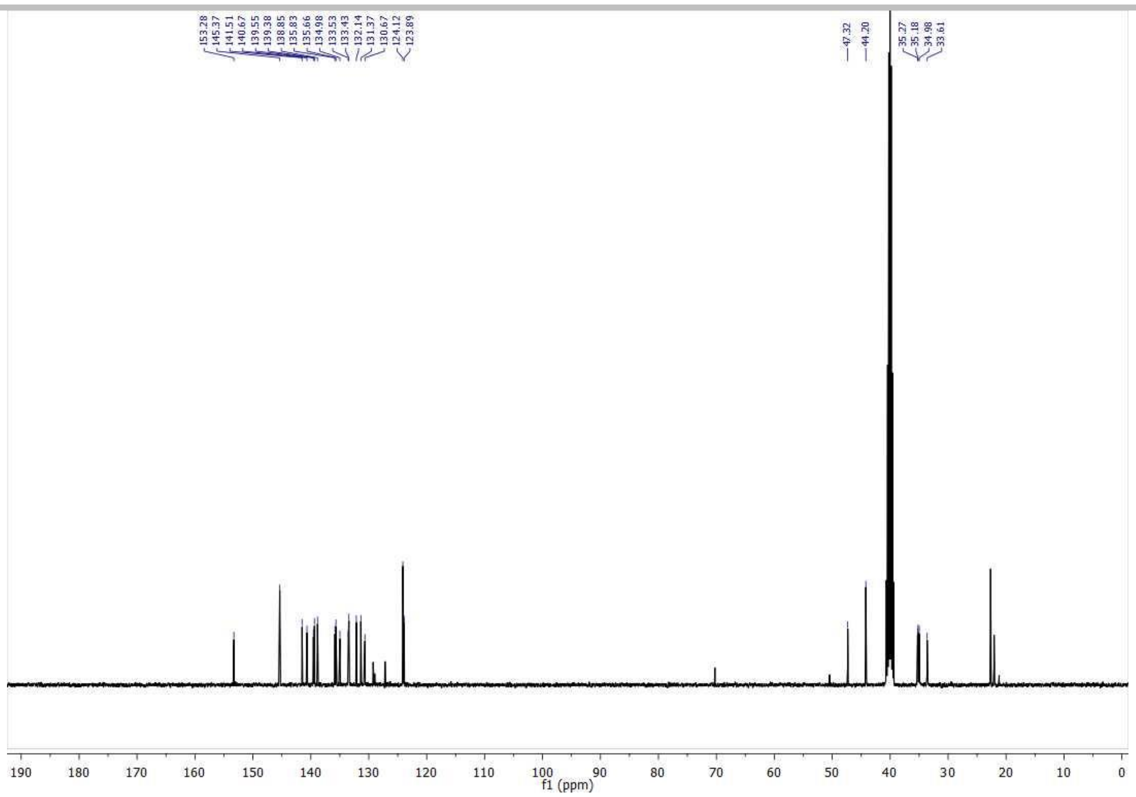

Figure S 16: ^{13}C NMR (101 MHz, DMSO-d_6) spectrum of (E)-4-((*S*$_P$)-4-vinyl[2.2]paracyclophane)-1-methyl pyridin-1-ium iodide(**6a**, (*S*$_p$)-MVCP)

6. Sample Preparation

The stock solutions were prepared in DI-Water and kept in the fridge at +8°C for storage. The insulin stock solution was prepared in 10 mM phosphate buffer acidified with HCL to pH 2.7 and kept in the freezer at -20°C for storage. The phenyl-β-D-galactopyranoside and β-galactosidase stock solutions were prepared in 10 mM phosphate buffer using phosphoric acid and basified with NaOH to pH 5 and kept in the freezer at -20°C for storage. The concentration of the stock solutions of the dyes and the analytes were determined by UV- Vis absorption titration measurements unless stated otherwise. The molar extinction coefficient of the samples used for the determination of the concentration of their stock solutions by UV-Vis absorption titration are given in Table S 1.

For compounds featuring unreported molar extinction coefficient, the stock solutions were prepared by weighing in the required amount of the pure sample to attain the desired concentration. The concentration of the stock solution of the host **CB8** was determined by fluorescence titration against MPCP dye by exciting the sample at 368 nm and collecting the emission intensity at 531 nm. The concentration of the memantine stock solution was determined by a fluorescence-based indicator displacement assay using **CB8**⊃BC_2 as the chemosensor by exciting the sample at 445 nm and collecting the emission intensity at 520 nm.[7] The **MT** receptor and the epoxides, (1*R*,2*R*)-1-phenylpropylene oxide and (1*S*,2*S*)-1-phenylpropylene oxide, stock solutions were freshly prepared each time before the start of the measurements to avoid any aggregation of the receptor molecule and the hydrolysis of the epoxides respectively, unless stated otherwise. For the ECD and FDCD measurements, the host and guest concentrations required to form the host-guest complex were chosen such that a sufficient degree of complexation of either the host or guest (preferably ≥ 50%) is achieved.

Table S 1: Absorption maxima (λ_{max}) and molar extinction coefficients ($\varepsilon_{\lambda max}$) of the dyes and analytes used for the determination of the concentration of their stock solutions by UV-Vis absorption titration measurements.

**Sample**	**λ_{max} (nm)**	**$\varepsilon_{\lambda max}$ ($M^{-1}cm^{-1}$)**
**(*R*$_p$)-MPCP**	335	7,110[7]
**(*S*$_p$)-MPCP**	335	7,110[7]
**(*S*$_p$)-MVCP**	402	11,218
**MDPP**	413	26,000[9]
**CB8•MDPP**	443	55000[10]
**MT**	340	10830
**D-Phe**	257.6	195[11]
**L-Phe**	257.6	195[11]
**L-Trp-NH_2**	278	5,580[11]
**L-Trp-OMe**	278	5,580[11]
**L-Phe-L-Ala**	257.6	195[11]
**L-Phe-Gly**	257.6	195[11]
**L-Phe-L-Val**	257.6	195[11]
**L-Ala-L-Phe**	257.6	195[11]
**insulin**	276	6,020[12]
**rhodamine 6G**	529.8	116,000[11]

7. FDCD characteristics

7.1. General measurement protocol for FDCD measurements

All the measurements reported were conducted on a CD spectrometer equipped with an FDCD accessory that allows simultaneous measurement of both ECD and FDCD spectra. The spectrometer is equipped with two PMT detectors, one for ECD and one emission detector for FDCD. The latter is placed at a 90° angle to the sample holder and collects the total fluorescence. An additional facility also allows to change freely the detection wavelength using proper high aperture monochromators. The HT voltage applied to the PMT of both detectors can be kept either in auto mode (fixed DC/varying HT) or manual mode (fixed HT/ varying DC). The FDCD unit also comprises of a filter holder for long-pass filters. Appropriate long-pass filters were used for the FDCD measurements to avoid the scattered light from excitation wavelength, which could contaminate the observed emission, and to maximize the emitted light signal.

In conventional CD spectrometers, the HT voltage applied to PMT is always kept in auto mode. This implies all the measurements are taken at a fixed DC voltage (by automatically varying the HT voltage) and a direct comparison of the different measured spectra is possible. In FDCD, the HT voltage applied to PMT is always kept in manual mode and the measurements are taken at a fixed HT voltage (and varying DC voltage). The differential circularly polarized fluorescence excitation signal ($F_L - F_R = \Delta F$) thus obtained is proportional to the fluorescence intensity difference for the two circularly polarized excitations (and thus to the concentration) and the HT voltage applied. As the DC voltage is varied here, it can be desired to correct the ΔF data for the total fluorescence ($F_L + F_R$), in order to obtain a concentration-independent chiroptical quantity, the FDCD signal via FDCD = $(F_L - F_R) / (F_L + F_R)$. This can be achieved by collecting both the ΔF signal and DC voltage (=total fluorescence) in two separate channels at a fixed HT voltage during the measurement. The corrected FDCD spectrum is then obtained by dividing the ΔF with the DC voltage.

$$\Delta F = F_{\mathrm{L}} - F_{\mathrm{R}} \qquad \text{Eq. S1}$$

$$\mathrm{FDCD} = \frac{\Delta F}{DC} = \frac{F_{\mathrm{L}} - F_{\mathrm{R}}}{F_{\mathrm{L}} + F_{\mathrm{R}}} \qquad \text{Eq. S2}$$

Because the difference in intensity of fluorescence excited by right-hand circularly polarized light and left-hand circularly polarized light is normalized against the intensity of total fluorescence when calculating FDCD values, this procedure arrives at HT voltage-independent and concentration-independent value. In contrast, the Δ*F* data shows the variation in signal intensity depending on the sample concentration and the applied HT voltage. Thus, FDCD and Δ*F* data are often complementary and their combined analysis is most informative.

7.1.1. User guide for FDCD measurements

The FDCD measurement of any sample can be conducted by following the steps below:

1. The samples for FDCD measurements are prepared such that the absorbance value does not exceed 1 (to avoid signal saturation), and if possible, in a low viscosity solvent to avoid for photoselection artefacts (see section 9.1).
2. FDCD spectra are inherently chiral excitation spectra. Hence, prior to the measurement both the excitation and emission spectra of the sample is collected on a separate fluorescence spectrometer in order to choose the appropriate long-pass filter (LP-Filter) for the measurement. If the excitation and emission spectra do not overlap, the LP-Filter is chosen at the longer wavelength were the excitation spectra ends. However, in cases where scatter of the excitation wavelengths overlapped with the emission range, it was of primary concern to adjust the LP-filter to a longer wavelength to eliminate the scattered excitation light.
3. After selection of the LP-Filter, the HT voltage on PMT of FDCD detector (in manual mode) can be adjusted for the measurement at a value giving proper intensity of the fluorescence signal by simultaneously avoiding signal saturation (maximum DC ≤ 3 V). The HT voltage on PMT of ECD detector is kept in auto mode. If possible, all the measurements corresponding to one host-guest system (host-guest spectra, host alone, guest alone, baseline etc.) should be conducted at this fixed HT voltage. If it is not possible to select one common HT voltage, *e.g.* because of strong differences in emission intensity, the FDCD spectra obtained can be used for comparison instead of Δ*F* spectra, or the HT voltage-corrected Δ*F* spectra explained in section 7.2 can be applied.
4. Both the Δ*F* signal and the DC voltage (or total fluorescence) are then collected in two separate channels along with the ECD spectra for both the sample and the baseline solvent used.
5. The FDCD spectra is then obtained by first subtracting the baseline solvent from the sample signals in order to get baseline corrected Δ*F* and DC spectra, and finally dividing the baseline

corrected Δ*F* signal by the DC voltage to get the FDCD result. The FDCD data was only obtained in cases where a measurable Δ*F* signal was present.

- Δ*F* signal are most useful when comparing systems measured at the same HT voltage on the same spectrometer. They are also advantageous in situations where a change in the fluorescence (or concentration) of the system needs to be monitored, for *e.g.* in case of sensing studies.
- FDCD spectra are most useful when comparing systems which were measured at different HT voltages and on different spectrometers.

Note: In JASCO instruments, for FDCD measurements conducted in both auto mode (fixed DC/varying HT) and manual mode (fixed HT/ varying DC), the output signal is termed as "FDCD (in mdeg)" even though it should be termed as "Δ*F* (in Volts)" when using the manual mode and "FDCD (in mdeg)" when using the auto mode. This has to be noted when taking the measurements and corrected for according to the mode selected.

7.2. Comparison of Δ*F* signal collected at different HT voltages and correction function

When measuring an analyte concentration dependent data or in case of sensing studies where a change in concentration or fluorescence is monitored, the Δ*F* data needs to be used, as the FDCD value is concentration independent and does not give any signal change depending on the sample concentration. However, when considering the Δ*F* signals, a direct comparison between two measurements is possible only when the two are measured at the same HT voltage and the other measurement parameters like bandwidth, accumulations, data pitch and D.I.T are kept constant. Herein, a function that correlates the Δ*F* signal and HT voltage was developed to use in situations where measurements need to be taken at different HT voltage.

Initially, the Δ*F* signals were optimized with respect to the HT Voltage applied to the photomultiplier tube (PMT) of the FDCD detector (Figure S 17) and bandwidth (Figure S 18) using ammonium d-10-camphorsulfonic acid (d-10-ACS) as a reference molecule.

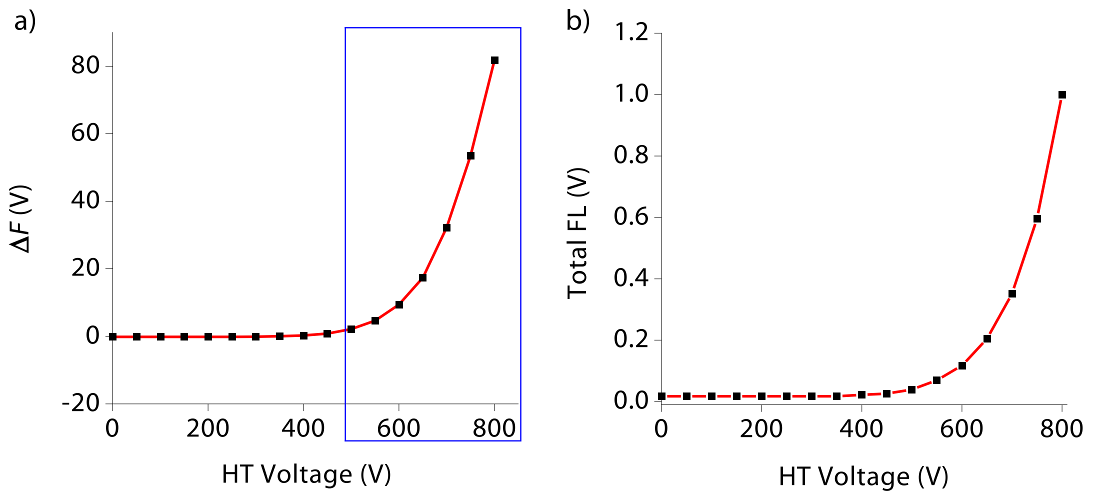

Figure S 17: Variation of (a) Δ*F* and (b) total fluorescence (or DC voltage) with HT Voltage (0-800 V) applied to the PMT of the FDCD detector in case of ammonium d-10-camphorsulfonic acid (d-10-ACS) as a reference molecule. The signal is monitored at 294 nm at a fixed bandwidth of 4 nm.

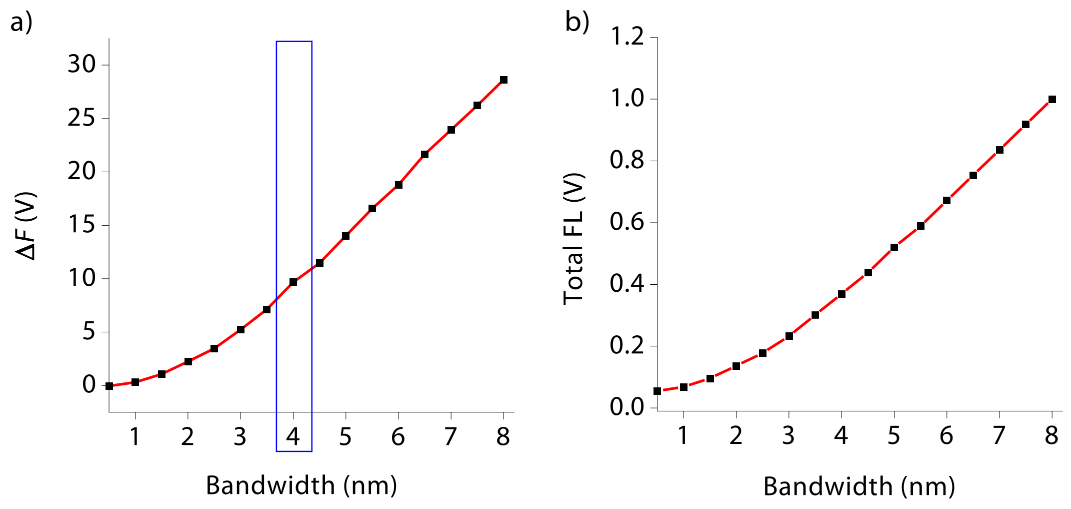

Figure S 18: Variation of (a) Δ*F* and (b) total fluorescence (or DC voltage) with bandwidth (0.5 – 8 nm) in case of ammonium d-10-camphorsulfonic acid (d-10-ACS) as a reference molecule. The signal is monitored at 294 nm at a fixed HT Voltage of 600 V applied to the PMT of the FDCD detector.

The results depicted in Figure S 17 and Figure S 18 show that it is preferable to fix the HT Voltage (if possible) and bandwidth in each measurement series and to compare only absolute Δ*F* signals for the same measurement parameters.

Both the Δ*F* signal and fluorescence intensity increases with HT voltage and have a maximum at HT = 800 V (Figure S 17). However, when utilizing higher sample concentrations, it is not possible to measure the Δ*F* signal at HT = 800 V (or higher HT voltages) due to fluorescence saturation. Hence for each sample measurement, the HT voltage needs to be set so that the fluorescence DC voltage does not exceed 4 V. In order to compare the Δ*F* signal obtained at different HT voltages, we now introduce a correction function which correlates the HT voltage and gives us the factor that needs to be multiplied to the measured Δ*F* signal to obtain the value of the HT voltage-corrected Δ*F* signal ($\Delta F_{corr.}$) when measured at an HT voltage of 800 V. The correction function is an exponential decay function with time constant parameter (Figure S 19) and is given below,

$$y = A_1 \times e^{-x/t1} + y_0$$

where,

y = factor multiplied to the measured Δ*F* signal to obtain the value of the HT voltage-corrected Δ*F* signal ($\Delta F_{corr.}$) at HT = 800 V

x = HT voltage used for the measurement

A_1 = amplitude = 96326.22321

$t1$ = time constant = 63.49548

y_0 = offset = 1

Now,

$$\Delta F_{\text{corr.}} = \text{factor} \times \text{measured } \Delta F \text{ signal}$$

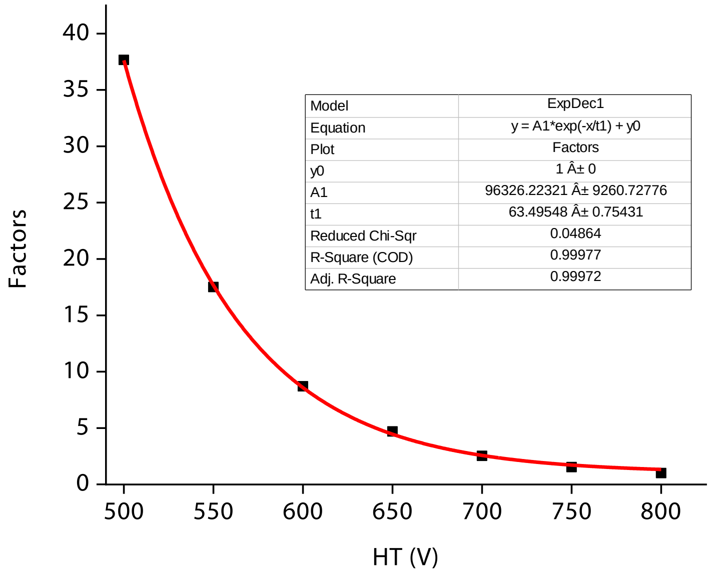

Figure S 19: Plot of the correction function with respect to HT voltage.

This correction function was applied to the analyte concentration dependent study in case of **CB8•MDPP** receptor in presence of varying concentration of L-Phe-L-Ala analyte (refer to Table S 4). The variation of HT voltage-corrected ΔF signal ($\Delta F_{corr.}$) and ECD signal with respect to L-Phe-L-Ala analyte concentration is depicted in Figure S 20 (a, b). Both $\Delta F_{corr.}$ signal and ECD signal behave linear and increases with increasing analyte concentration.

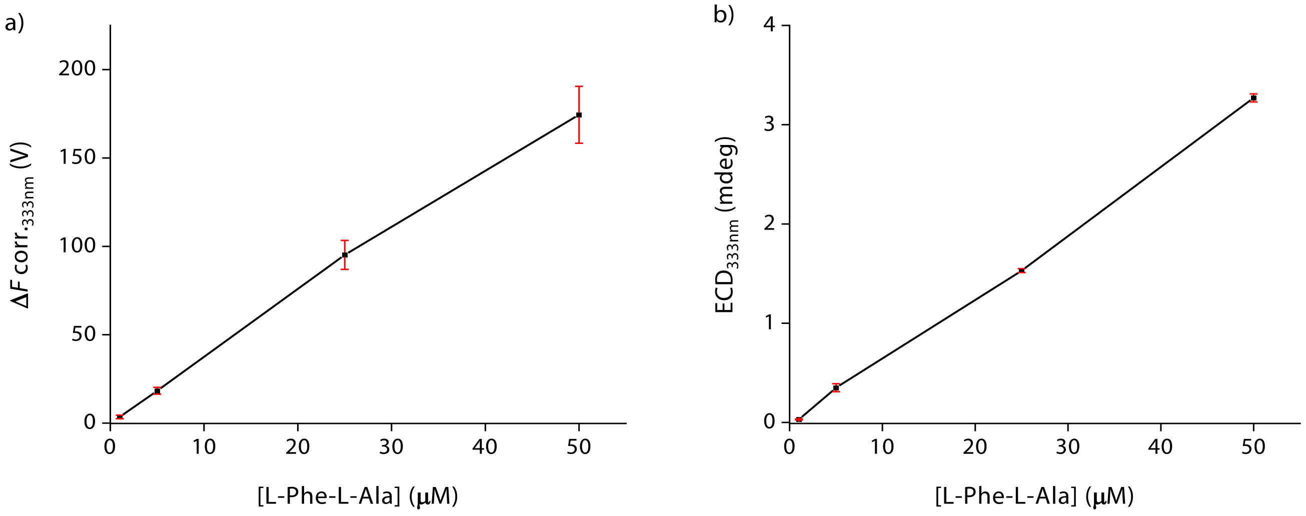

Figure S 20: Variation of (a) $\Delta F_{corr.}$ signal and (b) ECD signal of **CB8•MDPP** receptor in presence of varying concentration of L-Phe-L-Ala analyte. The vertical error bars for both the $\Delta F_{corr.}$ and ECD signal values are depicted in the graph.

7.3. Conversion of ECD and FDCD ellipticity values to molar circular dichroism values

The experimentally recorded ECD ellipticity values (θ in mDeg) can be converted to molar circular dichroism values ($\Delta\varepsilon$ in M^{-1} cm^{-1}) according to Eq. S3

$$\Delta\varepsilon = \frac{\theta}{(32980 \times \mathrm{l} \times \mathrm{c})} \qquad \text{Eq. S3}$$

where c is the concentration of the sample in mol L^{-1} and l is the path length of the cell in cm.

The experimentally recorded FDCD values can be corrected for absorbance and converted to $\Delta\varepsilon$ by following Eq. S4.[13-15]

$$\Delta\varepsilon = \varepsilon_\mathrm{L} - \varepsilon_\mathrm{R} = \frac{2 \times \mathrm{S} \times (1 - 10^{-A})}{(\mathrm{c} \times \mathrm{l} \times 10^{-A} \times \ln 10)}; \quad S = k\left(\frac{F_\mathrm{L} - F_\mathrm{R}}{F_\mathrm{L} + F_R}\right) \qquad \text{Eq. S4}$$

where k is the instrument proportionality constant, F_L and F_R are the fluorescence with left and right circularly polarized excitation, A is the absorbance of the sample, c is the concentration of the sample in mol L^{-1} and l is the path length of the cell in cm.

As the k value is a conversion constant specific for each machine, it was initially determined at the standard FDCD spectrometer at INT laboratory using 0.0024 M ammonium d-10-camphorsulfonic acid (d-10-ACS) as the reference. For a non-viscous solution of a single chiral and fluorophoric molecule like d-10-ACS, the molar circular dichroism calculated from both ECD and FDCD ellipticity values should arrive at the same spectra. Taking this into account, the k value was determined to be 5.934 x 10^{-5}. Hence the Eq. S4 can be written as follows:

$$\Delta\varepsilon = \varepsilon_{\mathrm{L}} - \varepsilon_{\mathrm{R}} = \frac{(5.154 \times 10^{-5}) \times (1 - 10^{-A}) \times (F_{\mathrm{L}} - F_{\mathrm{R}})}{(\mathrm{c} \times \mathrm{l} \times 10^{-A}) \times (F_{\mathrm{L}} + F_{\mathrm{R}})} \qquad \text{Eq. S5}$$

The $\Delta\varepsilon$ values obtained from ECD and FDCD ellipticity values were termed as $\Delta\varepsilon_{\mathrm{ECD}}$ and $\Delta\varepsilon_{\mathrm{FDCD}}$ respectively. Figure S 21 (d) depicts the $\Delta\varepsilon_{\mathrm{ECD}}$ and $\Delta\varepsilon_{\mathrm{FDCD}}$ values obtained from the ECD and FDCD ellipticity values in case of 0.0024 M d-10-ACS. Similarly refer to Figure S 23 for the calculation of the $\Delta\varepsilon_{\mathrm{ECD}}$ and $\Delta\varepsilon_{\mathrm{FDCD}}$ values in case of **CB8**•MPCP complex.

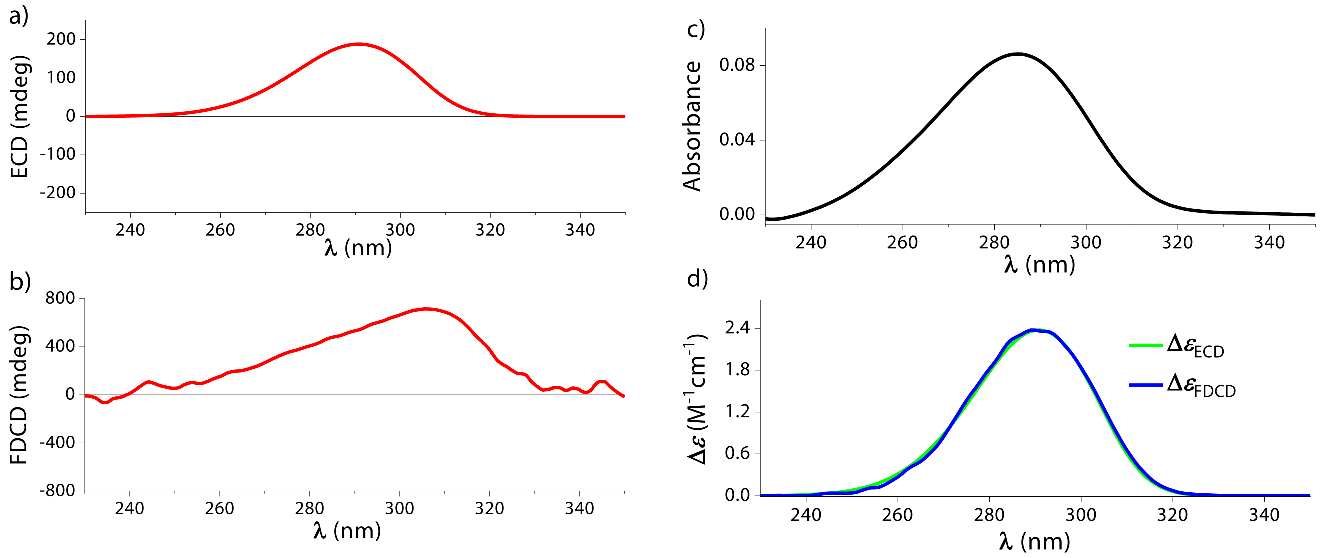

Figure S 21: (a) ECD, (b) FDCD and (c) absorbance spectra of 0.0024M d-10-ACS in DI-Water. Parameters used: HT = 970 V, BW = 4 nm, Acc = 20, LP-Filter = 380 nm, T = 25°C. (d) $\Delta\varepsilon_{\mathrm{ECD}}$ (green) and $\Delta\varepsilon_{\mathrm{FDCD}}$ (blue) spectra of 0.0024M d-10-ACS calculated from ECD and FDCD ellipticity values according to Eq. S3 and Eq. S5 respectively.

8. FDCD Measurements

8.1. Comparison of sensitivity for FDCD and ECD measurements and their combined use for detection of chiral analytes

8.1.1. Spectra of chiral MPCP and MVCP dye with CB8 receptor

FDCD is a fluorescence based chiral spectroscopy and may therefore be in principle more sensitive than absorbance-based ECD.[13, 14, 16, 17] In order to access the sensitivity of FDCD measurements over ECD for supramolecular systems, the direct binding of the chiral chromophoric MPCP dye enantiomers ((R_p)-MPCP and (S_p)-MPCP) with the achiral cucurbit[8]uril (**CB8**) host[7] was studied. As expected for a chiral emissive dye, both (R_p)-MPCP and (S_p)-MPCP shows ECD and Δ*F* signals even in the absence of **CB8**. On addition of **CB8** a slight bathochromic shift in the both the ECD and Δ*F* signals was observed which was also accompanied by a strong enhancement in the dye fluorescence on **CB8** binding (Figure S 22). This enhancement in the fluorescence of the dye molecule on addition of **CB8** is also reflected in the strong enhancement in the Δ*F* signal (Figure S 22 (b)). The Δ*F* spectra was then divided by the total fluorescence (= DC voltage) to obtain the concentration-independent FDCD spectra (Figure S 22 (d)). Analysis of the Δ*F* component of the FDCD value is more suited in this system as well as for sensitivity-assessment studies described further below, as the latter normalizes the signal against the total fluorescence. Both the ECD and FDCD spectra of the enantiomers roughly behave as mirror images exhibiting opposite signs, as is expected by theory for the enantiomeric complexes.[18] The molar circular dichroism value (Δ*ε*) is calculated from the ECD and FDCD ellipticity values following Eq. S3-S5 and is depicted in Figure S 23.

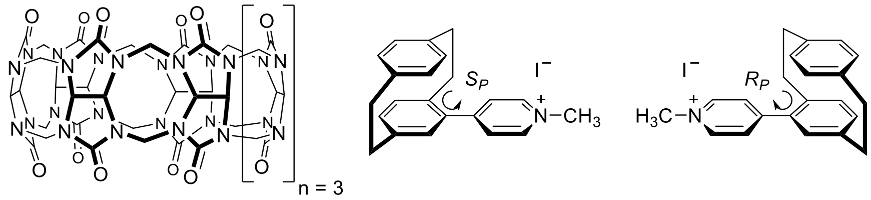

CB8 (S_p)-MPCP (R_p)-MPCP

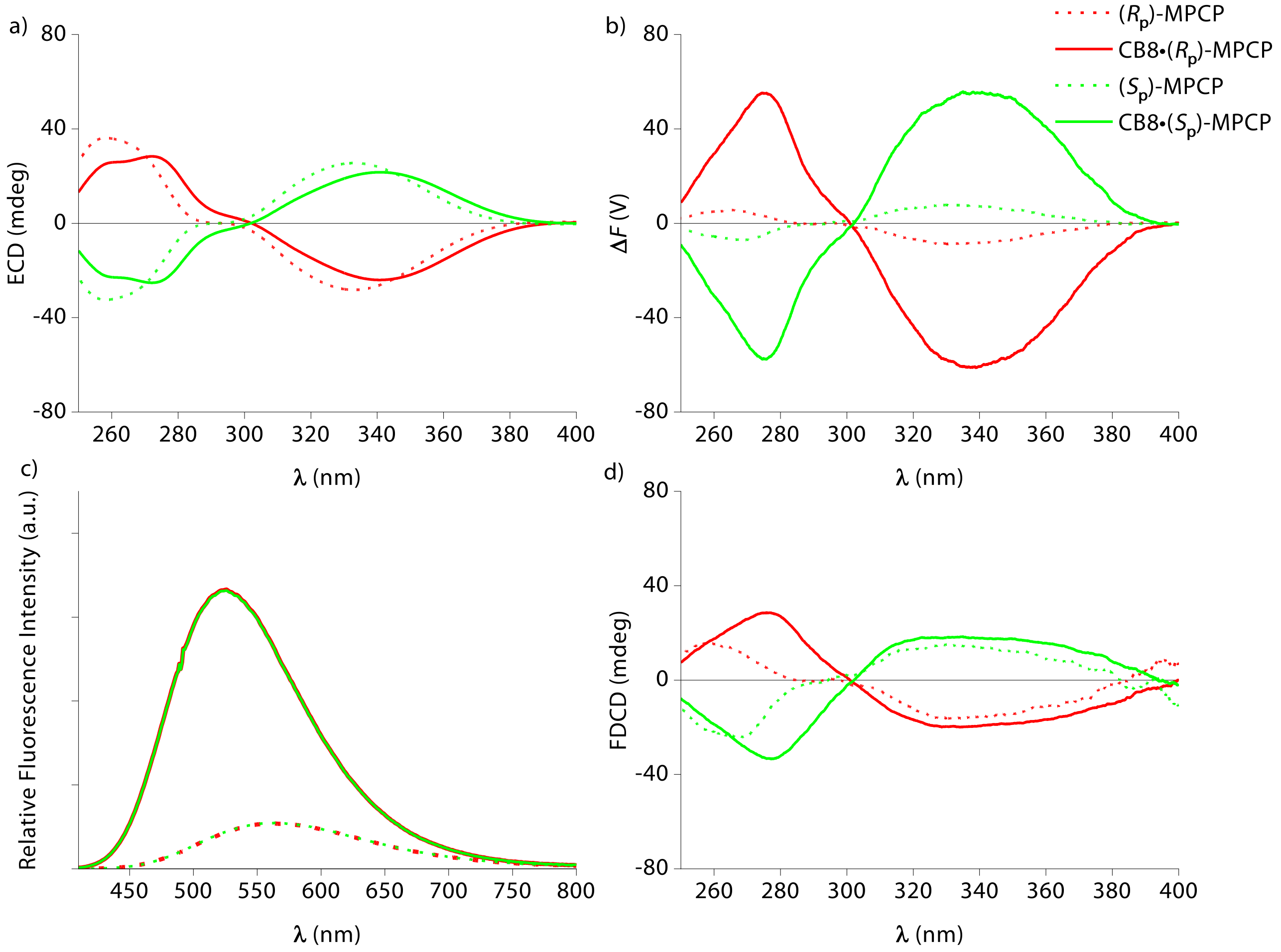

Figure S 22: (a) ECD and (b) Δ*F* spectra of the MPCP dye enantiomers ((R_p))-MPCP (45 μM) and ((S_p))-MPCP (45 μM) in the absence and presence of **CB8** (45 μM) in DI-Water. Parameters used: HT = 650 V, BW = 4 nm, Acc = 20, LP-Filter = 420 nm, T = 25°C. (c) Enhancement in the fluorescence intensity of (R_p)-MPCP (45 μM) and (S_p)-MPCP (45 μM) upon addition of **CB8** (45 μM) in DI-Water, λ_{exc} = 350 nm. (d) FDCD spectra of (R_p)-MPCP (45 μM) and (S_p)-MPCP (45 μM) in the absence and presence of **CB8** (45 μM) in DI-Water.

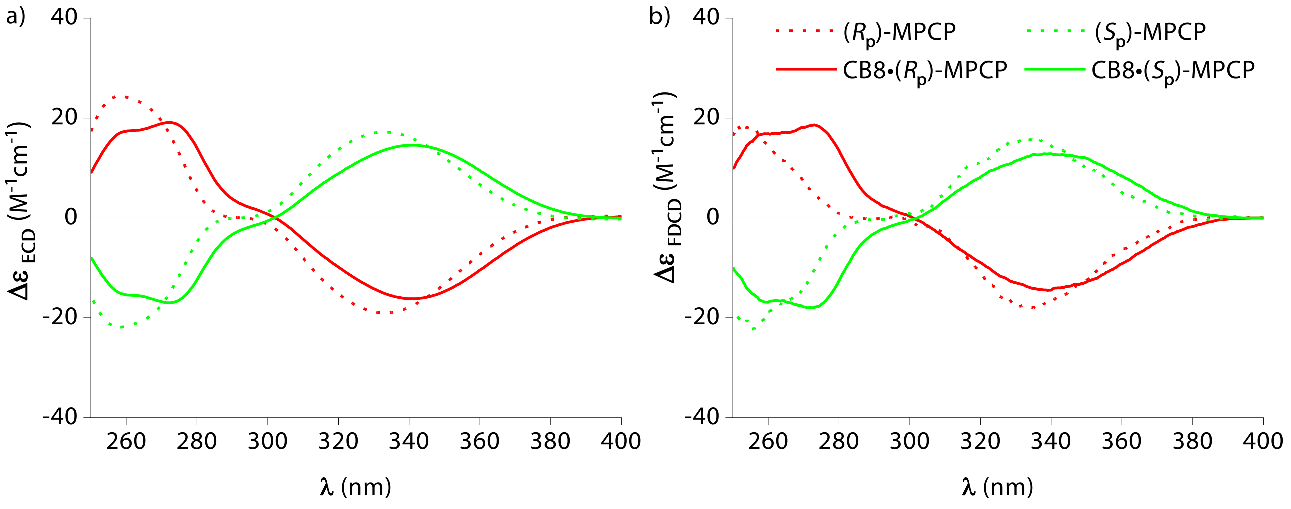

Figure S 23: $\Delta\varepsilon_{ECD}$ and $\Delta\varepsilon_{FDCD}$ spectra of the MPCP dye enantiomers (R_p)-MPCP (45 μM) and (S_p)-MPCP (45 μM) in the absence and presence of **CB8** (45 μM) in DI-Water calculated from ECD and FDCD ellipticity values using Eq. S3-S5.

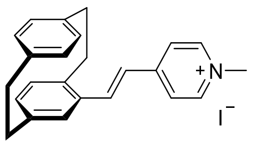

(S_p)-MVCP

The new emissive and chiral paracyclophane-dye, (S_p)-MVCP was also investigated to study its binding properties with **CB8**. (S_P)-MVCP shows absorption bands in the 350 nm to 450 nm region, which is by 46 nm more red-shifted absorption when compared to the MPCP dye. As expected for a chiral emissive dye, (S_p)-MVCP shows both ECD and Δ*F* signals even in the absence of **CB8**. On addition of **CB8** a slight bathochromic shift in the both the ECD and Δ*F* signals was observed which was also accompanied by a strong enhancement in the fluorescence intensity, and in the Δ*F* signal on binding to **CB8** (Figure S 24).

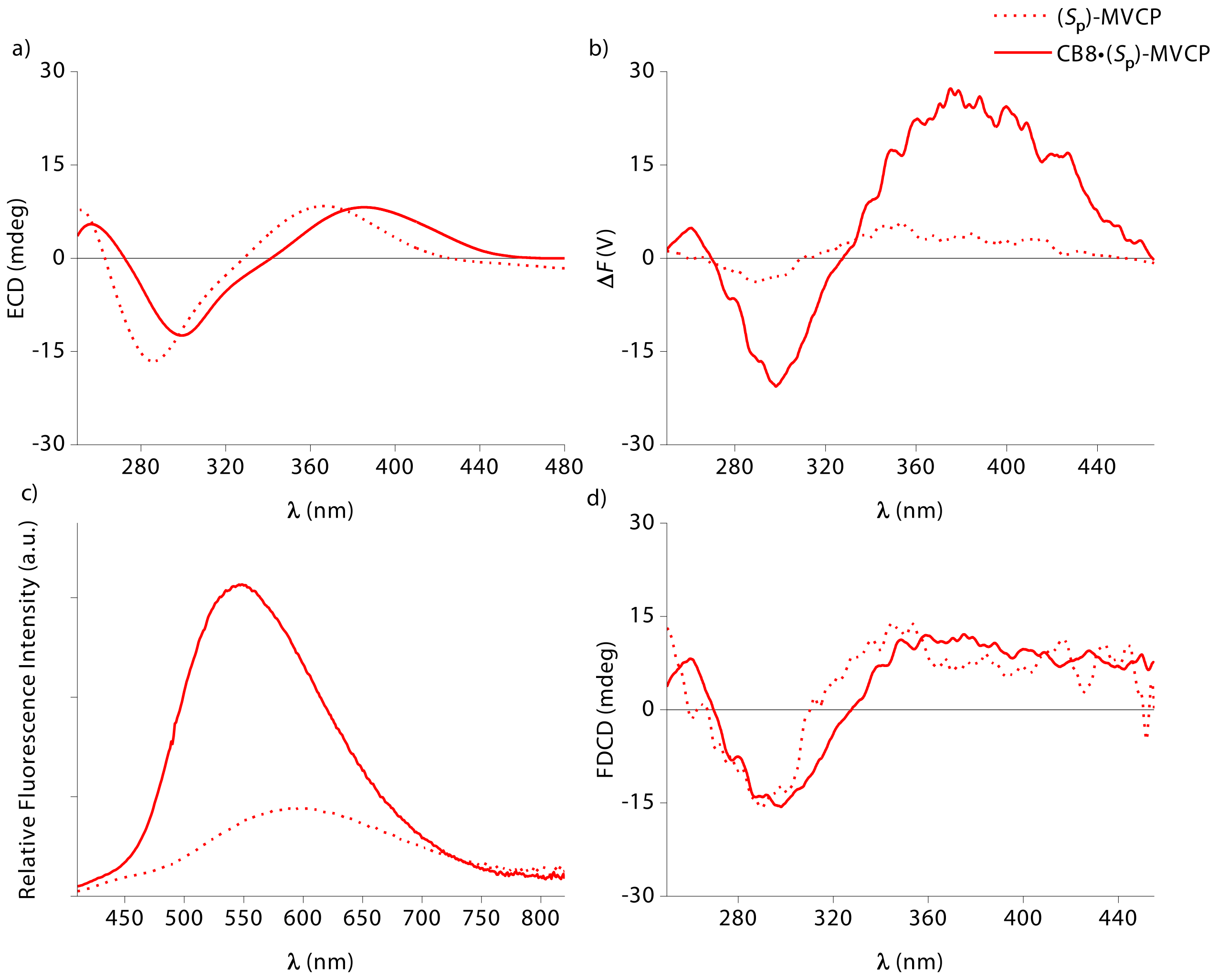

Figure S 24: (a) ECD and (b) Δ*F* spectra of (S_p)-MVCP (45 µM) in the absence and presence of **CB8** (45 µM) in DI-Water. Parameters used: HT = 800 V, BW = 4 nm, Acc = 20, LP-Filter = 480 nm, T = 25°C. (c) Enhancement in the fluorescence intensity of (S_p)-MVCP (45 µM) upon addition of **CB8** (45 µM) in DI-Water, λ_{exc} = 400 nm. (d) FDCD spectra of (S_p)-MVCP (45 µM) in the absence and presence of **CB8** (45 µM).

To check the sensitivity of FDCD over ECD measurements, the ECD and Δ*F* spectra of the enantiomers (R_p)-MPCP and (S_p)-MPCP in the presence of **CB8** were measured by diluting the samples down to 100 nM (Figure S 25 (a, b)). It can be seen that it is not possible to detect clear ECD signals for the dyes and their host-dye complexes at such a low concentration (although complex formation is ensured with logK_a = 12.6), while the Δ*F* signal is still observable, indicating that Δ*F* can be more sensitive than ECD.

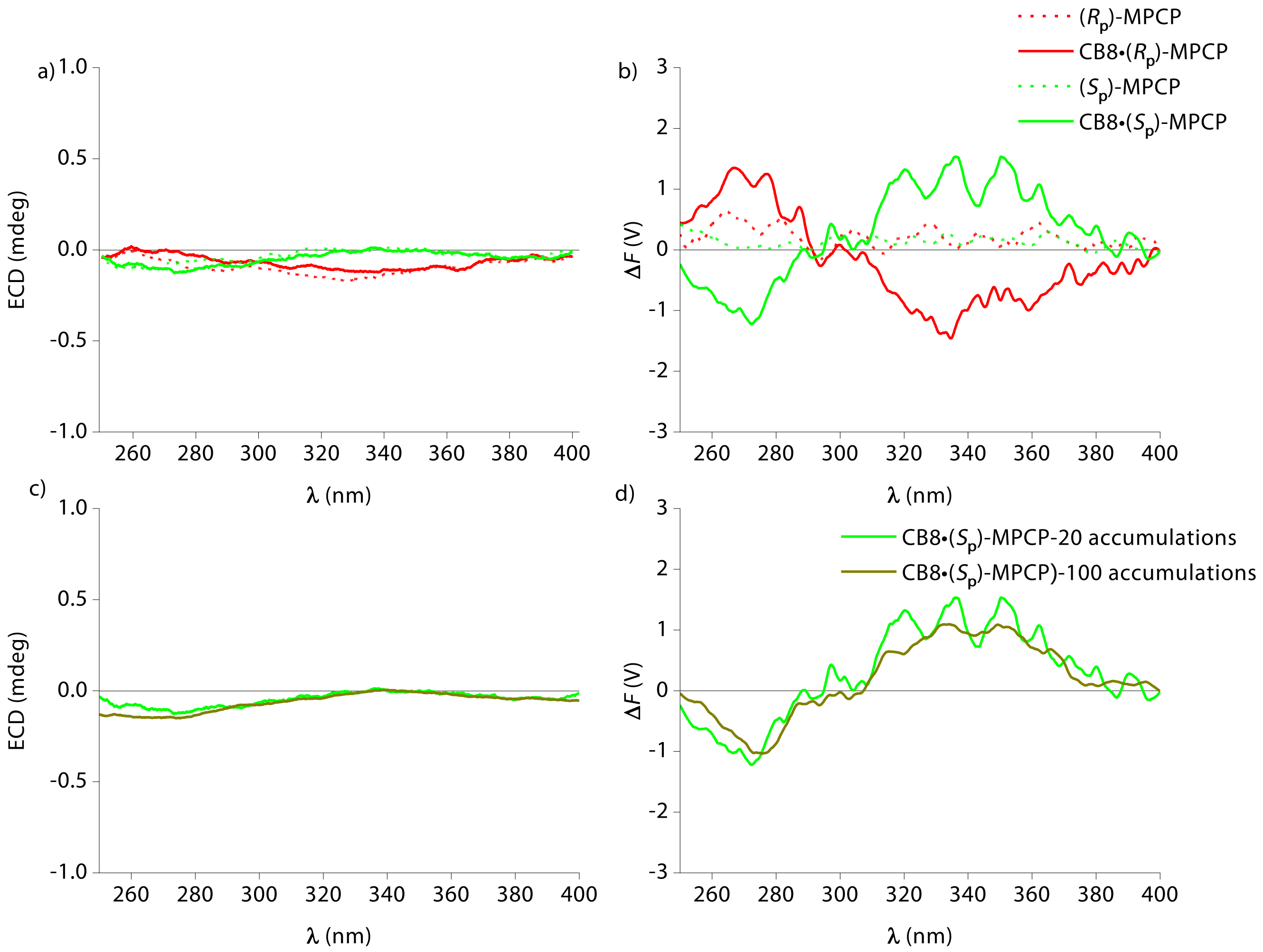

Figure S 25: (a) ECD and (b) Δ*F* spectra of MPCP dye enantiomers (R_p)-MPCP (100 nM) and (S_p)-MPCP (100 nM) in the absence and presence of **CB8** (100 nM) in DI-Water. Parameters used: HT = 800 V, BW = 4 nm, Acc = 20, LP-Filter = 420 nm, T = 25°C. (c) ECD and (d) Δ*F* spectra of (S_p)-MPCP (100 nM) in the presence of **CB8** (100 nM) in DI-Water at 20 accumulations (green line) and at 100 accumulations (dark yellow line). Parameters used: HT = 800 V, BW = 4 nm, LP-Filter = 420 nm, T = 25°C.

FDCD and Δ*F* spectra show a "noisy character", especially when going to low micromolar concentration of the analyte. This problem was overcome by either increasing the number of accumulations of the measurement or by using single wavelength time course Δ*F* measurements instead of measuring the whole spectrum.

The ECD and Δ*F* signals for the **CB8•**(S_p)-MPCP complex measured at both 20 and 100 accumulations (Figure S 25 (c, d)) shows that the spectrum is reproducible and the signal to noise ratio improves with increasing accumulations, at the cost of longer measurement times. Faster experiments can be performed through single wavelength measurements. The data is displayed in Table S 2 for the **CB8•**(R_p)-MPCP and Table S 3 for the **CB8•**(S_p)-MPCP complexes going to a low concentration of 100 nM and 50 nM. The Δ*F* values at the selected wavelength were consistent with the ones obtained from the whole spectral recording. Again, Δ*F* signal can be reliably obtained even at very low concentration (50 nM) while ECD signals are not reproducibly distinct from the background in this concentration range (Table S 2 and Table S 3). The FDCD values obtained in all cases shows – within error – the concentration independence of this value, as is expected. The difference in the Δ*F* and FDCD values (not exact mirror images) for the enantiomeric **CB8•**(R_p)-MPCP and **CB8•**(S_p)-MPCP complexes in Table S 2 and Table S 3 can be due to the higher *%ee* for (R_p)-MPCP dye compared to (S_p)-MPCP (see Figure S 5).

Table S 2: Single Point-Time Course ECD and Δ*F* measurements of **CB8•**(R_p)-MPCP complex when going to a low concentration of 100 nM and 50 nM. The parameters are kept constant for each individual set of measurements.

Sample[a]	*ECD (mdeg)*	*ΔF*[b] *(V)*	*FDCD*[c] *(mdeg)*
**CB8•(R_p)-MPCP 100nM**	-0.11	-1.34	-36.3
**CB8•(R_p)-MPCP 50nM**	-0.01	-0.82	-40.0

a) Measured at λ_{obs} = 335 nm, BW = 4 nm, Data Pitch = 30 s, D.I.T = 30 s, $t_{measure}$ = 10 min, T = 25°C. b) HT = 800 V, LP-Filter = 420 nm. c) Obtained by dividing the Δ*F* value with the DC voltage (total fluorescence).

Table S 3: Single Point-Time Course ECD and Δ*F* measurements of **CB8•**(S_p)-MPCP complex when going to a low concentration of 100 nM and 50 nM. The parameters are kept constant for each individual set of measurements.

Sample[a]	*ECD (mdeg)*	*ΔF*[b] *(V)*	*FDCD*[c] *(mdeg)*
**CB8•(S_p)-MPCP 100nM**	0.09	1.13	29.9
**CB8•(S_p)-MPCP 50nM**	-0.05	0.55	29.0

a) Measured at λ_{obs} = 335 nm, BW = 4 nm, Data Pitch = 30 s, D.I.T = 30 s, $t_{measure}$ = 10 min, T = 25°C. b) HT = 800 V, LP-Filter = 420 nm. c) Obtained by dividing the Δ*F* value with the DC voltage (total fluorescence).

8.1.2. Analyte spectra with CB8•MDPP receptor

The chemosensing ensemble composed of the large macrocycle **CB8** and the fluorescence dye MDPP form a 1:1:1 hetero-ternary complex (associative binding) in the presence of chiral analytes such as amino acids, peptides, and proteins. It was previously shown that these **CB8**-ternary complexes show analyte-characteristic induced ECD signals (spectroscopic fingerprints).[10, 19] Herein, we applied FDCD spectroscopy in comparison to ECD spectroscopy to these systems in order to assess the sensitivity differences, and because we believed that the combination of FDCD and ECD gives additional, useful chiroptical information about the analyte present. First, the supramolecular complex of **CB8•MDPP** in presence of the enantiomers D-Phe and L-Phe was investigated (Figure S 26). Both enantiomers show induced ECD and induced Δ*F* and FDCD signals, which roughly behave as mirror images exhibiting opposite signs. The addition of D-Phe or L-Phe to **CB8•MDPP** was accompanied by a small shift in the emission peaks along with a slight enhancement in the fluorescence intensity of the **CB8•MDPP** receptor (Figure S 26 (c)). Because the resulting ternary complex of **CB8•MDPP** with D-Phe or L-Phe are emissive, the Δ*F* and FDCD signals can be observed. In contrast, the tryptophan derivatives L-Trp-NH_2 and L-Trp-OMe shows an induced ECD signal in presence of the **CB8•MDPP** receptor, however exhibits no induced Δ*F* and FDCD signals (Figure S 26). This is explained by the strong quenching in the fluorescence intensity of the **CB8**•MPPP receptor upon addition of the analyte in case of tryptophan derivatives (Figure S 26 (c)).

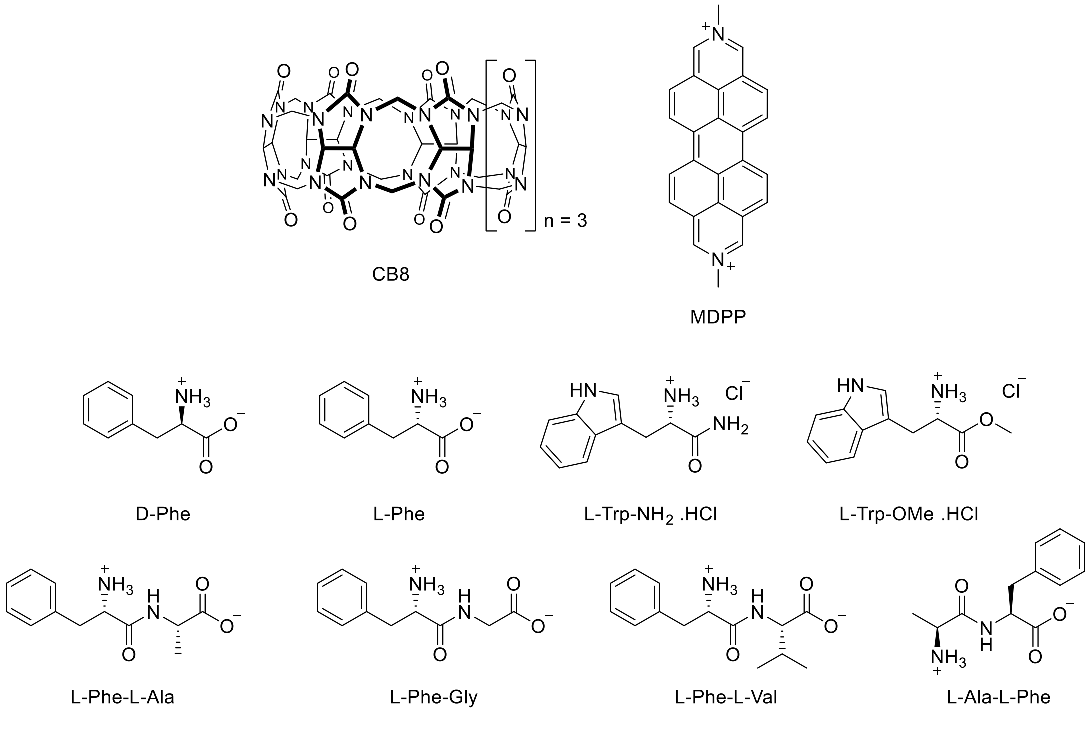

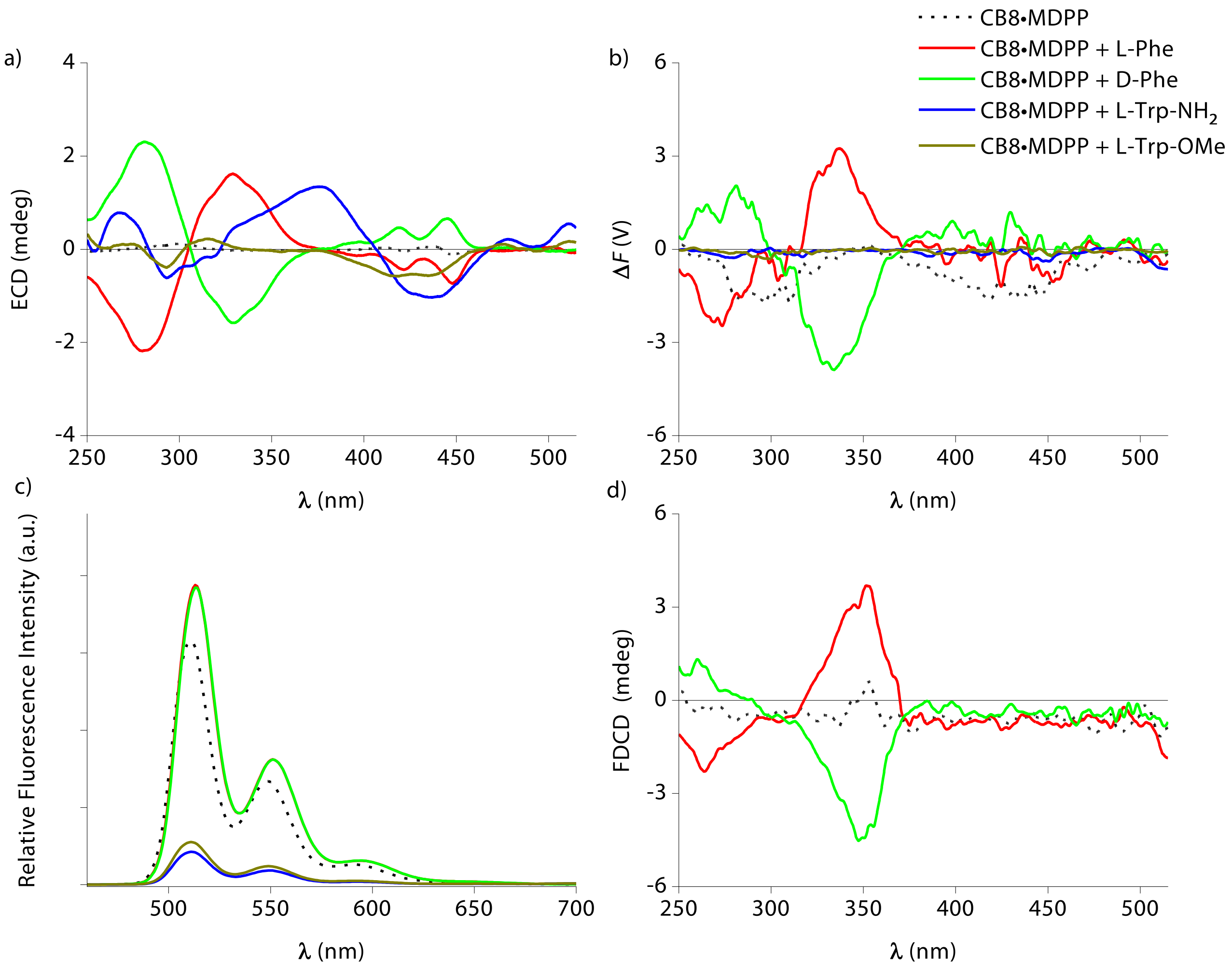

Figure S 26: (a) ECD and (b) Δ*F* spectra of **CB8•MDPP** (20 μM) in the presence of the amino acids D-Phe (50 μM), L-Phe (50 μM), L-Trp-NH_2 (50 μM) and L-Trp-OMe (50 μM) in DI-Water. Parameters used: HT = 520 V, BW = 4 nm, Acc = 20, LP-Filter = 515 nm, T = 25°C, (c) Enhancement in the fluorescence of **CB8•MDPP** receptor (20 μM) upon addition of L-Phe and D-Phe (50 μM) and quenching in the fluorescence of **CB8•MDPP** receptor (20 μM) upon addition of L-Trp-NH_2 and L-Trp-OMe (50 μM) in DI-Water, λ_{exc} = 450 nm. (d) FDCD spectra of **CB8•MDPP** (20 μM) in the presence of the amino acids D-Phe (50 μM) and L-Phe (50 μM).

In these examples, both the FDCD values as well as the Δ*F* signals were informative and useful. However, when using the Δ*F* spectra, signal artefacts arising from the **CB8•MDPP** receptor alone must be considered and corrected for (see Section 9.1.1 for further information). FDCD values should only be presented for systems with a measurable Δ*F* spectrum (and thus, for instance, not in case of the **CB8•MDPP** complexes with Trp-species).

Upon addition of Phe-containing dipeptides such as L-Phe-L-Ala, L-Phe-Gly and L-Phe-L-Val to the **CB8•MDPP** receptor, both induced ECD and FDCD signals can be observed (Figure S 27). Note the clear differences between the ECD and FDCD spectra of the N-terminal Phe containing dipeptides from the C-terminal Phe variants, for *e.g.* in case of L-Ala-L-Phe.

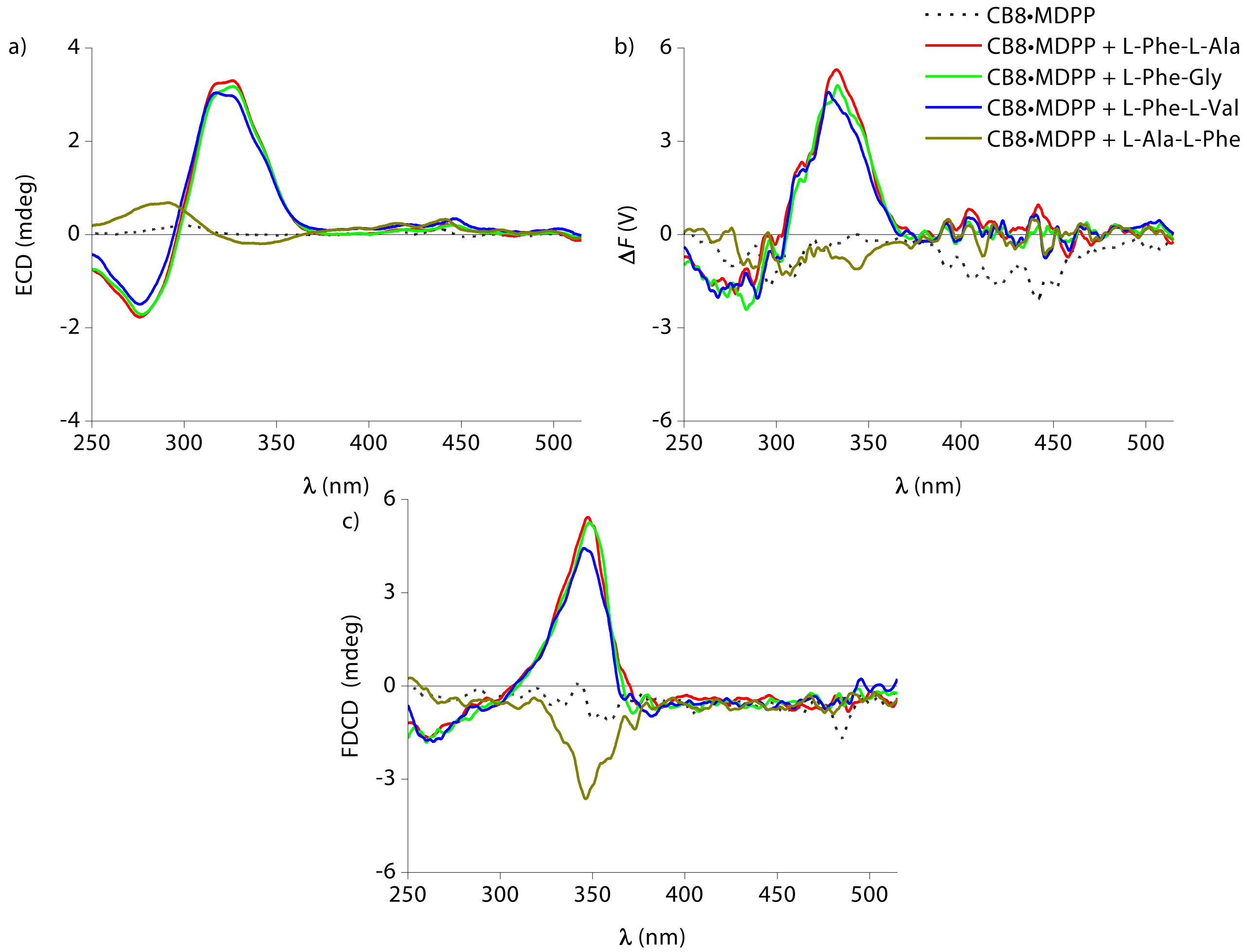

Figure S 27: (a) ECD, (b) Δ*F* and (c) FDCD spectra of **CB8•MDPP** (20 µM) in the presence of several dipeptides (50 µM) in DI-Water. Parameters used: HT = 510 V, BW = 4 nm, Acc = 20, LP-Filter = 515 nm, T = 25°C

In order to assess if FDCD is more sensitive than ECD measurements in case of this supramolecular system, the induced ECD and Δ*F* signals in **CB8•MDPP** receptor at varying analyte concentration in case of L-Phe-L-Ala was measured by using single point-time course Δ*F* and ECD measurements. The results showed that the ECD signal completely vanishes at a concentration of 1 µM of the analyte but the Δ*F* signal is still present even at a concentration of 0.50 µM (Table S 4) (For the concentration dependent studies, the ratio of **CB8•MDPP** receptor to analyte was always kept at 1:2.5). Because the binding constant of the complex (K_a = 9.80 (±0.98) x·10^5 M^{-1})[10] is limiting the concentration range where the complex remains stable, it was not feasible to dilute the solution further. The values obtained were

consistent and the standard deviation for the measurements were calculated in case of L-Phe-L-Ala from three independent measurements by varying the measurement parameters in each set (Table S 5). It can be convenient in Δ*F* measurements to increase the HT voltage on the detector in order to increase the signal intensity when measuring at low concentrations; in fact, this option is not available for ECD measurements. Note that when measurements are conducted at different HT voltages, a direct comparison between these Δ*F* values is not possible! Instead, a correction function which relates the HT voltage and the measured Δ*F* signal can be used to obtain the value of the HT voltage-corrected Δ*F* signal (ΔF_{corr}), see also section 7.2. The FDCD values again show the concentration independence of this value.

Table S 4: Single Point-Time Course ECD and Δ*F* measurements of **CB8•MDPP** receptor in presence of varying concentration of L-Phe-L-Ala analyte. The parameters were kept constant for each individual set of measurements.

Sample[a]	***ΔF (V)***	***HT***[b] ***(V)***	***ΔF_{corr}***[c] ***(V)***	***FDCD***[d] ***(mdeg)***	***ECD (mdeg)***
**CB8•MDPP 20 µM + L-Phe-L-Ala 50 µM**	6.29	520	174.45	3.45	3.27
**CB8•MDPP 10 µM + L-Phe-L-Ala 25 µM**	4.64	540	95.17	3.75	1.53
**CB8•MDPP 2 µM + L-Phe-L-Ala 5 µM**	2.80	620	18.30	3.52	0.35
**CB8•MDPP 0.4 µM + L-Phe-L-Ala 1 µM**	2.18	760	3.51	3.91	0.03
**CB8•MDPP 0.2 µM + L-Phe-L-Ala 0.5 µM**	1.02	800	1.02	3.00	0.01

a) Measured at BW = 4 nm, λ_{obs} = 333 nm, Data Pitch = 30 s, D.I.T = 30 s, $t_{measure}$ = 10 min, LP-Filter = 515 nm, T = 25°C; b) HT Voltage applied to the PMT of the FDCD detector. Hence this is only influencing the Δ*F* value, not the ECD value (different detectors); c) Corrected Δ*F* value at HT = 800 V. d) Obtained by dividing the Δ*F* value with the DC voltage (total fluorescence).

Table S 5: Standard deviation for the Single Point-Time Course ECD and Δ*F* measurements of **CB8•MDPP** receptor in the presence of varying concentration of L-Phe-L-Ala analyte. The standard deviation was obtained from three measurements by varying the measurement parameters in each individual set.

Sample	***ΔF (V)***	***S.D***[a] ***(V)***	***ECD (mdeg)***	***S.D***[a] ***(mdeg)***
**CB8•MDPP 20 µM + L-Phe-L-Ala 50 µM**	6.29	0.29	3.27	0.04
**CB8•MDPP 10 µM + L-Phe-L-Ala 25 µM**	4.64	0.20	1.53	0.02
**CB8•MDPP 2 µM + L-Phe-L-Ala 5 µM**	2.80	0.15	0.35	0.04
**CB8•MDPP 0.4 µM + L-Phe-L-Ala 1 µM**	2.18	0.52	0.03	0.005

a) Standard deviation obtained from three measurements by varying the measurement parameters in each individual set.

To verify that the Δ*F* signal obtained at a 1 µM concentration of L-Phe-L-Ala analyte in presence of **CB8•MDPP** is a true signal and not an artefact arising from the instrument, **CB8•MDPP•**L-Phe-L-Ala receptor-analyte complex was destroyed through the addition of memantine, which sequesters **CB8** (Ka ≈ 10^{11} M^{-1}).[20] Hence, the induced Δ*F* signal in the **CB8•MDPP•**L-Phe-L-Ala receptor-analyte complex was monitored at 333 nm before and after the addition of achiral and achromophoric memantine (Table S 6). The disappearance of the induced Δ*F* signal for the **CB8•MDPP•**L-Phe-L-Ala complex upon addition of memantine gave a first clear indication that the Δ*F* signal observed for the **CB8•MDPP•**L-Phe-L-Ala complex at submicromolar concentration is not an artefact.

Table S 6: Single Point-Time Course Δ*F* measurements of the **CB8•MDPP** receptor (0.40 µM) in the presence of L-Phe-L-Ala (1 µM) and in the presence of both L-Phe-L-Ala (1 µM) and memantine (1 µM) in DI-Water. The measurements were repeated to check the reproducibility of the results obtained.

Sample	**Δ*F*^{a} *(V)***	
	Test 1	***Test 2***
**CB8•MDPP + L-Phe-L-Ala**	2.00	1.71
**CB8•MDPP + L-Phe-L-Ala + memantine**	-0.33	-0.09

a) Measured at HT = 760 V, BW = 4 nm, λ_{obs} = 333nm, Data Pitch = 30 s, D.I.T = 30 s, $t_{measure}$ = 10 min, LP-Filter = 515 nm, T = 25°C

The addition of memantine to the **CB8•MDPP•**L-Phe-L-Ala receptor-analyte complex also alters the fluorescence intensity as memantine displaces both the dye and analyte from the **CB8** cavity. Hence, to show that the disappearance of the Δ*F* signal of the complex at 333 nm is not due to this decrease in the fluorescence intensity, L-Phe-L-Ala was partially displaced from the **CB8•MDPP•**L-Phe-L-Ala complex by the addition of an excess of the weaker-binding D-Phe, resulting in the formation of the **CB8•MDPP•**D-Phe complex. The **CB8•MDPP** receptor shows a negative Δ*F* signal at 338 nm in the presence of D-Phe (Figure S 26(b)). The addition of D-Phe to the **CB8•MDPP•**L-Phe-L-Ala complex should therefore create a racemic effect with no reduction in the fluorescence intensity. As expected the single point Δ*F* measurements of the **CB8•MDPP•**L-Phe-L-Ala complex shows a lowering of the induced Δ*F* signal at 333 nm on addition of D-Phe (Table S 7). These results gave additional evidence that the Δ*F* signal observed for the **CB8•MDPP•**L-Phe-L-Ala complex at submicromolar concentration is not an artefact.

Table S 7: Single Point-Time Course Δ*F* measurements of the **CB8•MDPP** receptor (0.40 µM) in the presence of L-Phe-L-Ala (1 µM) and in the presence of both L-Phe-L-Ala (1 µM) and D-Phe (2 µM) in DI-Water. The measurements were repeated to check the reproducibility of the results obtained.

Sample	**Δ*F*[a] *(V)***	
	Test 1	***Test 2***
**CB8•MDPP + L-Phe-L-Ala**	2.00	1.71
**CB8•MDPP + L-Phe-L-Ala + D-Phe**	1.00	0.65

a) Measured at HT = 760 V, BW = 4 nm, λ_{obs} = 333nm, Data Pitch = 30 s, D.I.T = 30 s, $t_{measure}$ = 10 min, LP-Filter = 515 nm, T = 25°C

The sensitivity check of FDCD over ECD was also conducted in case of the dipeptides, L-Phe-Gly and L-Phe-L-Val using **CB8•MDPP** receptor by going to lower analyte concentrations (Table S 8 and Table S 9). The results obtained demonstrate again at least an order of magnitude higher sensitivity of Δ*F* component over ECD for this particular supramolecular system.

Table S 8: Single Point-Time Course ECD and Δ*F* measurements of **CB8•MDPP** receptor in presence of varying concentration of L-Phe-Gly analyte. The parameters were kept constant for each individual set of measurements

***Sample*[a]**	**Δ*F* *(V)***	***HT*[b] *(V)***	**ΔF_{corr}[c] *(V)***	***ECD* *(mdeg)***
**CB8•MDPP 20 µM + L-Phe-Gly 50 µM**	6.79	520	188.31	3.17
**CB8•MDPP 10 µM + L-Phe-Gly 25 µM**	4.95	540	101.53	1.40
**CB8•MDPP 2 µM + L-Phe-Gly 5 µM**	3.35	620	21.89	0.38
**CB8•MDPP 0.4 µM + L-Phe-Gly 1 µM**	1.78	760	2.87	-0.05

a) Measured at BW = 4 nm, λ_{obs} = 333 nm, Data Pitch = 30 s, D.I.T = 30 s, $t_{measure}$ = 10 min, LP-Filter = 515 nm, T = 25°C; b) HT Voltage applied to the PMT of the FDCD detector. Hence this is only influencing the Δ*F* value, not the ECD value (different detectors); c) Corrected Δ*F* value at HT = 800 V

Table S 9: Single Point-Time Course ECD and Δ*F* measurements of **CB8•MDPP** receptor in presence of varying concentration of L-Phe-L-Val analyte. The parameters were kept constant for each individual set of measurements.

***Sample*[a]**	**Δ*F* *(V)***	***HT*[b] *(V)***	**ΔF_{corr}[c] *(V)***	***ECD* *(mdeg)***
**CB8•MDPP 20 µM + L-Phe-L-Val 50 µM**	5.22	520	144.77	2.62
**CB8•MDPP 10 µM + L-Phe-L-Val 25 µM**	4.01	540	82.25	1.20
**CB8•MDPP 2 µM + L-Phe-L-Val 5 µM**	2.22	620	14.51	0.21
**CB8•MDPP 0.4 µM + L-Phe-L-Val 1 µM**	1.25	760	2.01	0.02

a) Measured at BW = 4 nm, λ_{obs} = 333 nm, Data Pitch = 30 s, D.I.T = 30 s, $t_{measure}$ = 10 min, LP-Filter = 515 nm, T = 25°C; b) HT Voltage applied to the PMT of the FDCD detector. Hence this is only influencing the Δ*F* value, not the ECD value (different detectors); c) Corrected Δ*F* value at HT = 800 V

The degree of complexation of the analyte can be increased by using an excess of the achromophoric/emissive host, for instance in case of **CB8•MDPP** host-guest complexes. For *e.g.*, see the enhancement in the ECD and Δ*F* signal on titration of **CB8•MDPP** receptor to L-Phe-Gly analyte (5 µM) in DI-water. A sufficient degree of complexation has been achieved on addition of 11 µM of the receptor after which the ECD and Δ*F* signal showed no significant increase. This desirable behaviour is a consequence of the chiroptical response that only originates from the host-guest complex. On the other hand, the fluorescence intensity showed a steady increase on titrating **CB8•MDPP** receptor to L-Phe-Gly which is the result of both the increase of the degree of complexation and the increase in fluorescence emission originating from the unbound receptor.

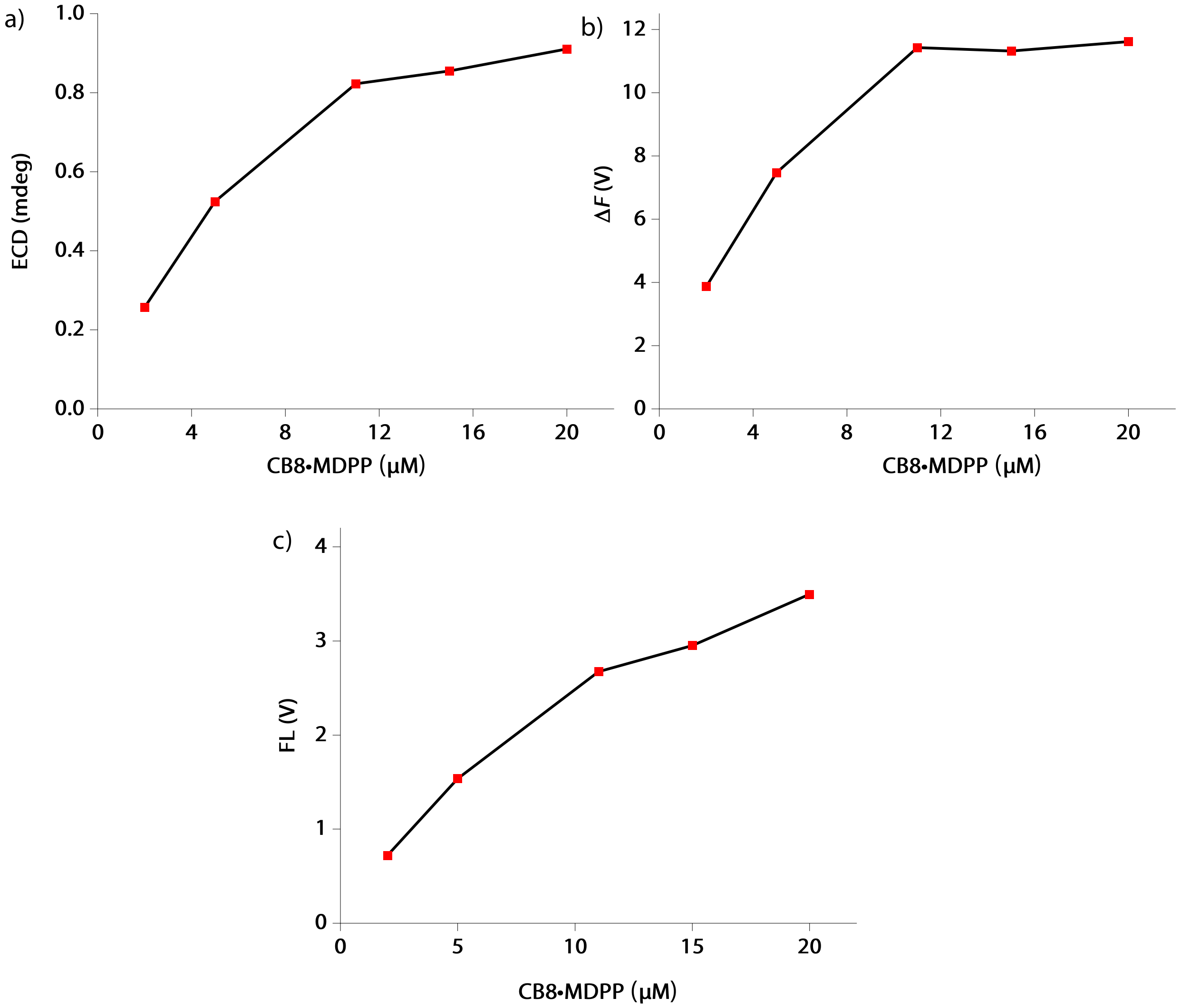

Figure S 28: Variation in the (a) ECD, (b) Δ*F* and (c) total fluorescence (or DC voltage) signal on titration of **CB8•MDPP** receptor to a solution containing L-Phe-Gly (5µM) in DI-water when monitored at 333 nm by single point-time course measurements. Parameters used: HT = 640 V, BW = 4 nm, λ_{obs} = 333 nm, Data Pitch = 30 s, D.I.T = 30 s, $t_{measure}$ = 10 min,LP-Filter = 515 nm, T = 25°C.

8.1.3. FDCD and ECD measurements for rapid, label-free end-point and continuous reaction monitoring using CB8•MDPP receptor

8.1.3.1. Racemisation of amino acids and dipeptides

FDCD along with ECD can be a useful technique for the sensitive, label-free monitoring of reactions of chiral analytes in real time. To demonstrate this concept for the monitoring of catalytic reactions, the induced ECD and Δ*F* signals arising in case of supramolecular systems in the presence of chiral analytes was used. Several neutral free amino acids, *e.g.* L-Phe, were reported to undergo racemisation and decomposition in polar organic solvents such as DMF and ethylene glycol under alkaline conditions, while this phenomena do not occur, or are largely decreased, in water under the same alkaline conditions.[21] However, in order to monitor the racemisation event, previous analytical protocols need to follow a lengthy derivatization procedure for HPLC analysis, making a direct detection difficult. In our previous study we have adopted ECD spectroscopy for monitoring the racemisation of both L-Phe and L-Phe-Gly in water, DMF and ethylene glycol using the induced ECD signals generated in acyclic CB[*n*] and **CB8•MDPP** reporter pairs in the presence of these chiral analytes.[9] There it was demonstrated that the time-consuming post-sample treatment can be omitted. Herein, Δ*F* was utilized for the end point monitoring of racemisation of amino acids using **CB8•MDPP** reporter pair, comparing the results to our previous ECD protocol. Concretely, L-Phe and L-Phe-Gly were heated in the presence of 1.2eq. of K_2CO_3 at 130°C for 2 hours in DMF, ethylene glycol and water and the resulting racemisation was evaluated by measuring the induced Δ*F* and ECD signals arising on adding aliquots of L-Phe and L-Phe-Gly from the reaction mixture to **CB8•MDPP** receptor in DI-water before and at fixed time points of the reaction (Figure S 29 and Figure S 30).

The racemisation of L-Phe and L-Phe-Gly was also monitored at room temperature (25°C) in the presence of 1.2 eq. of K_2CO_3 for 24 hours in DMF with the help of **CB8•MDPP** receptor (Figure S 29 and Figure S 30– DMF (control)). The amino acids/ dipeptide was found to be quite stable under these conditions; the racemisation of the amino acid/ dipeptide occurs only strongly upon heating in DMF. In all cases the Δ*F* signals were monitored instead of the FDCD values, as the former shows the real change in the signal with time corresponding to the changes in concentration and fluorescence.

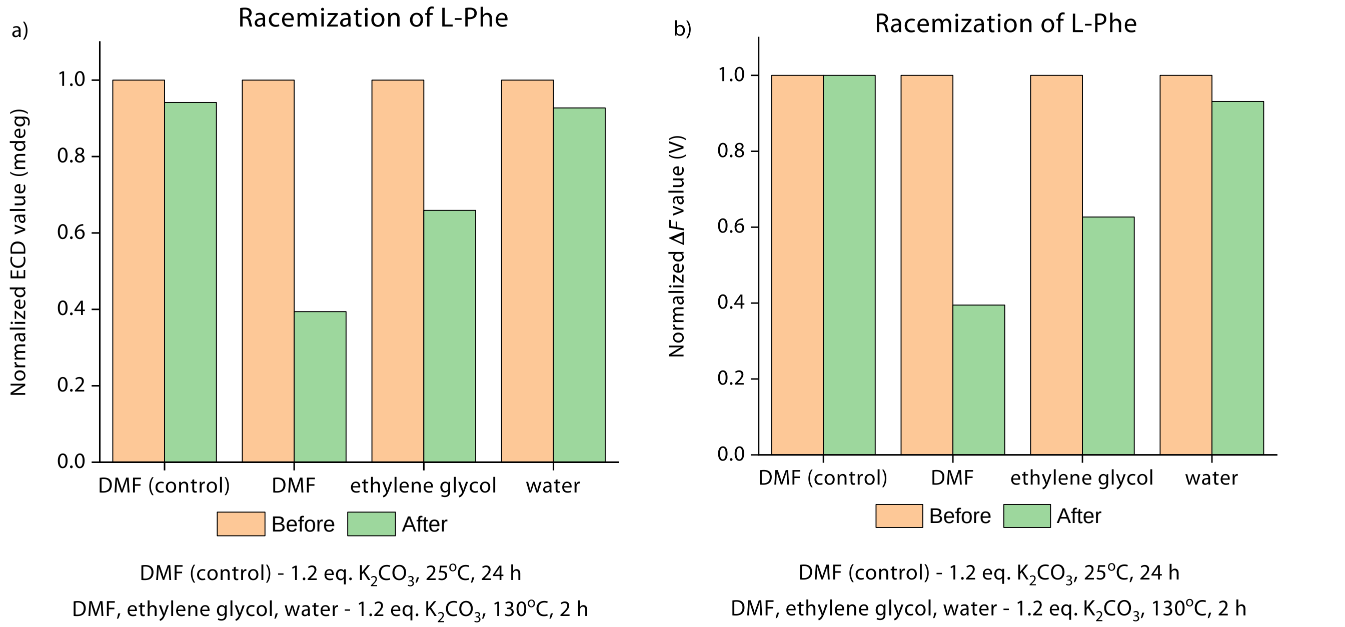

Figure S 29: Monitoring the racemisation of L-Phe in the presence of **CB8•MDPP** before and after the completion of reaction in DMF, ethylene glycol and water using single point-time course (a) ECD and (b) Δ*F* measurements. Measured at λ_{obs} = 338 nm, BW = 4 nm, Data Pitch = 30 s, D.I.T = 30 s, $t_{measure}$ = 10 min. The signals are monitored in DI-water at a concentration of **CB8•MDPP** of 20 µM in the presence of excess of L-Phe (≈100 µM) from the reaction mixture. The DMF (control) shows the control reaction in DMF when the reaction mixture was kept at room temperature instead of heating to 130°C.

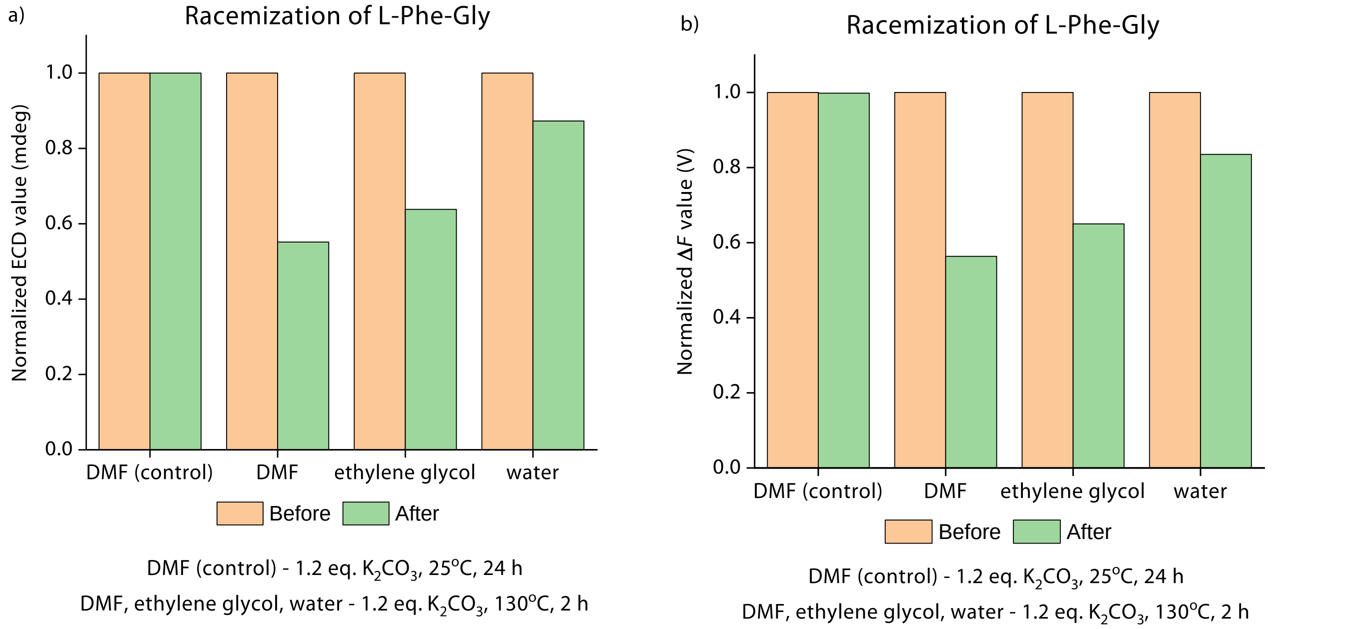

Figure S 30: Monitoring the racemisation of L-Phe-Gly in the presence of **CB8•MDPP** before and after the completion of reaction in DMF, ethylene glycol and water using single point-time course (a) ECD and (b) Δ*F* measurements. Measured at λ_{obs} = 333 nm, BW = 4 nm, Data Pitch = 30 s, D.I.T = 30 s, $t_{measure}$ = 10 min. The signals are monitored in DI-water at a concentration of **CB8•MDPP** of 20 µM in the presence of excess of L-Phe-Gly (≈100 µM) from the reaction mixture. The DMF (control) shows the control reaction in DMF when the reaction mixture was kept at room temperature instead of heating to 130°C.

The results depicted in Figure S 29 and Figure S 30 show that both Δ*F* and ECD can be used for the real time monitoring of the racemisation of amino acids and dipeptides which is quicker and convenient than the established HPLC routines. Δ*F* can have the additional advantages of its higher sensitivity, *e.g.* if detection at low micromolar or nanomolar concentrations is required.

8.1.3.2. Hydrolysis of chiral epoxides

The chemosensing ensemble **CB8•MDPP** also shows useful induced ECD and Δ*F* signals in presence of the chiral epoxide, (1*R*,2*R*)-1-phenylpropylene oxide (Figure S 31). Chiral epoxides can be converted into many functional groups and are important intermediates in chemistry[22, 23] and biology.[24] Hence the hydrolysis of (1*R*,2*R*)-1-phenylpropylene oxide was investigated with the help of **CB8•MDPP** receptor in different solvent systems, *i.e.* 50 mM acetate buffer at pH 4.75, DI-Water at pH 7 and 50 mM carbonate buffer at pH 10 (Figure S 32) by using the induced Δ*F* and ECD signals generated. The results plotted in Figure S 32 demonstrate that the system is stable under basic conditions (monitored over a duration of 14 hours). However, the system undergoes hydrolysis in both neutral water and particularly under acidic condition. This observation is in agreement with expectation for secondary-carbon containing epoxides that likely follow a H^+ catalysed S_N1 type reaction mechanism (Scheme S 1).[25, 26] This shows that Δ*F* along with ECD can in principle be used for the real-time monitoring of chemical reactions involving chiral analytes.

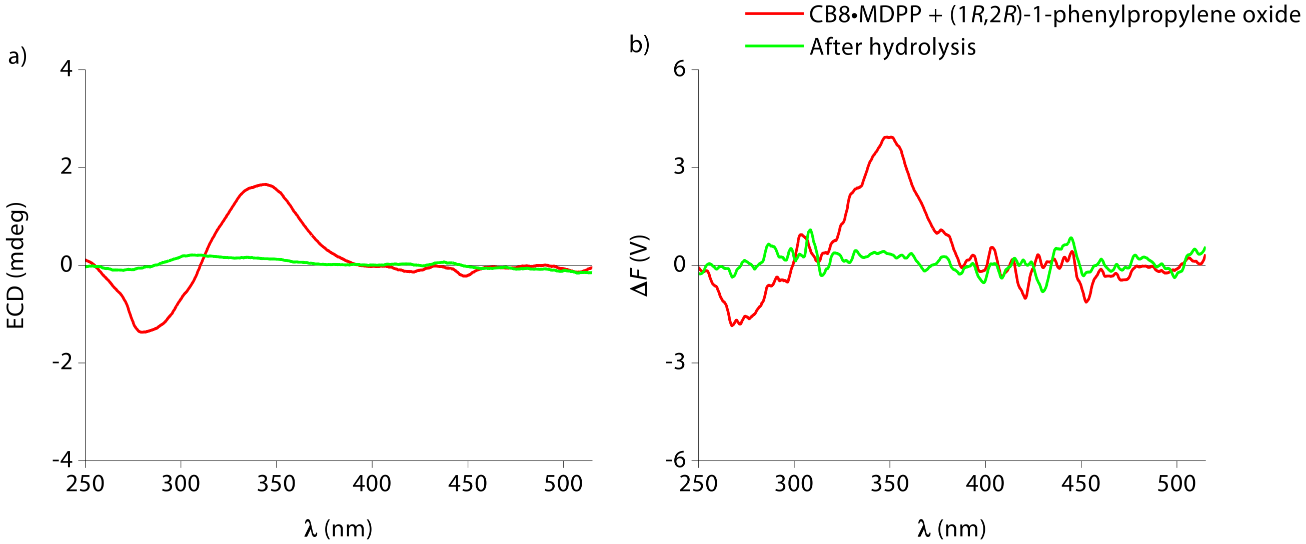

Figure S 31: (a) ECD and (b) Δ*F* spectra of **CB8•MDPP** (20 µM) in the presence of (1*R*,2*R*)-1-phenylpropylene-oxide (100 µM) (red line) in DI-Water and the spectra after hydrolysis (green line). Parameters used: HT = 520 V, BW = 4 nm, Acc = 20, LP-Filter = 515 nm, T = 25°C

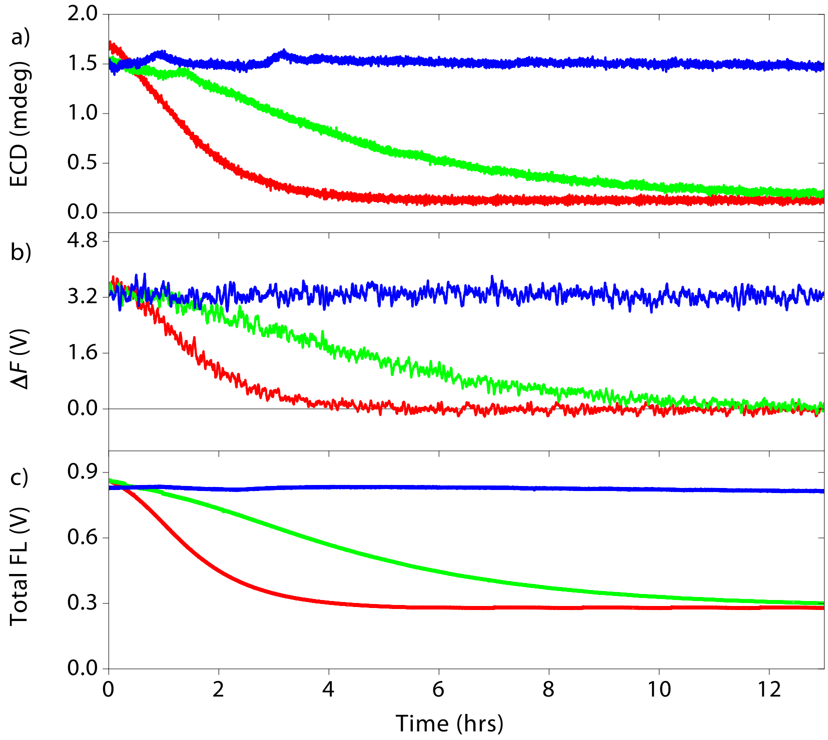

Figure S 32: Time Course (a) ECD, (b) Δ*F* and (c) total fluorescence (or DC voltage) measurements of **CB8•MDPP** (20 µM) in the presence of (1*R*,2*R*)-1-phenylpropylene oxide (100 µM) in different solvent systems: 50 mM acetate buffer at pH 4.75 (red), DI-Water at pH 7 (green) and 50 mM carbonate buffer at pH 10 (blue). Parameters used: HT = 520 V, BW = 4 nm, Acc = 20, LP-Filter = 515 nm, λ_{obs} = 350 nm, Data Pitch = 5 s, D.I.T = 8 s, $t_{measure}$ = 14 h, T = 25°C

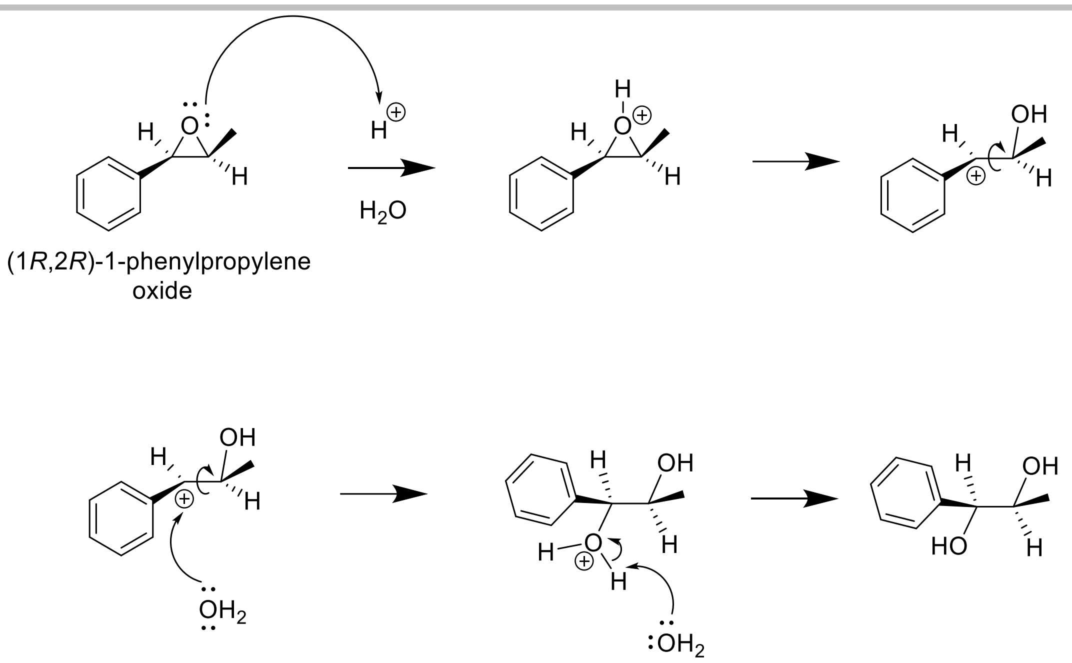

Scheme S 1: Schematic representation of the hydrolysis of secondary carbon containing epoxide ((1*R*,2*R*)-1-phenylpropylene oxide) following an S_N1 type reaction mechanism. Note that racemisation can occur in the carbocation intermediate through rotation along the C-C bond of the opened epoxide.

8.1.3.3. Monitoring of enzymatic reactions

The chemosensing ensemble **CB8•MDPP** also shows useful induced ECD and Δ*F* signals in presence of phenyl-β-D-galactopyranoside (Figure S 33). Phenyl-β-D-galactopyranoside is used as a substrate for detecting β-galactosidase enzymatic activity.[27, 28] The hydrolysis of phenyl-β-D-galactopyranoside by β-galactosidase was monitored with the help of Δ*F* and ECD signals generated using **CB8•MDPP** receptor. The reaction was monitored at 25°C in 10 mM phosphate buffer at pH 5 at 50 µM concentration of phenyl-β-D-galactopyranoside in presence of 10 µM of **CB8•MDPP** receptor. The enzyme (β-galactosidase) was present at a concentration of 43 µg/ml. The time course Δ*F* and ECD measurements shows a gradual decrease in the intensity of both induced Δ*F* and ECD signals upon addition of β-galactosidase hence allowing a real time monitoring of the enzymatic conversion (Figure S 34). Indeed, low µM concentrations of the substrate were sufficient for kinetic monitoring via ECD or Δ*F* signal detection.

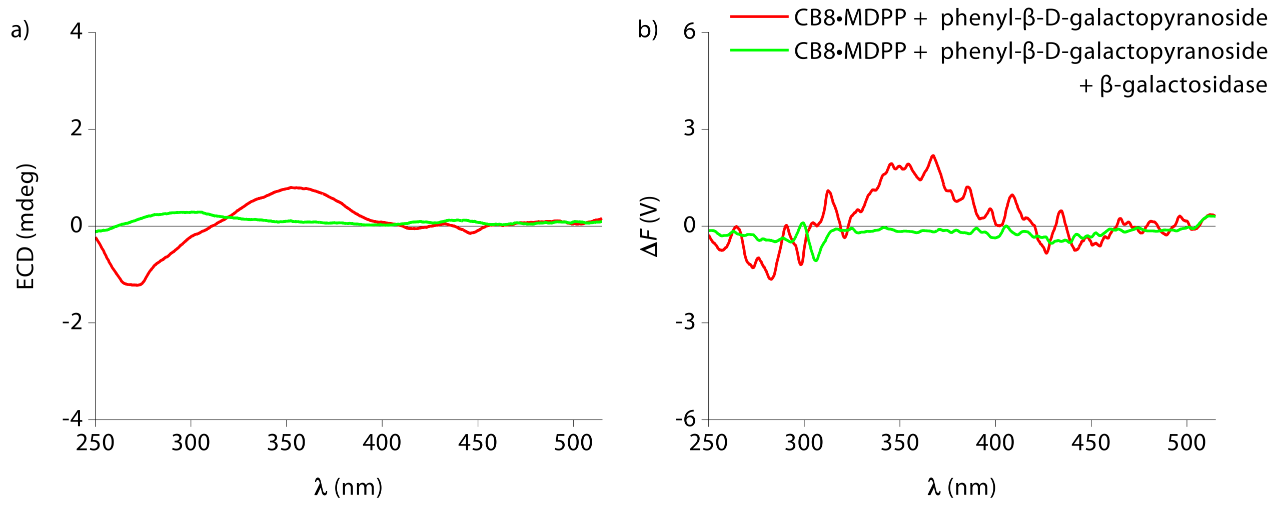

Figure S 33: (a) ECD and (b) Δ*F* spectra of **CB8•MDPP** (10 µM) in the presence of phenyl-β-D-galactopyranoside (50 µM) (red line) in 10 mM phosphate buffer at pH 5 and the spectra after hydrolysis of phenyl-β-D-galactopyranoside by β-galactosidase (43 µg/ml) (green line). Parameters used: HT = 550 V, BW = 4 nm, Acc = 20, LP-Filter = 515 nm, T = 25°C

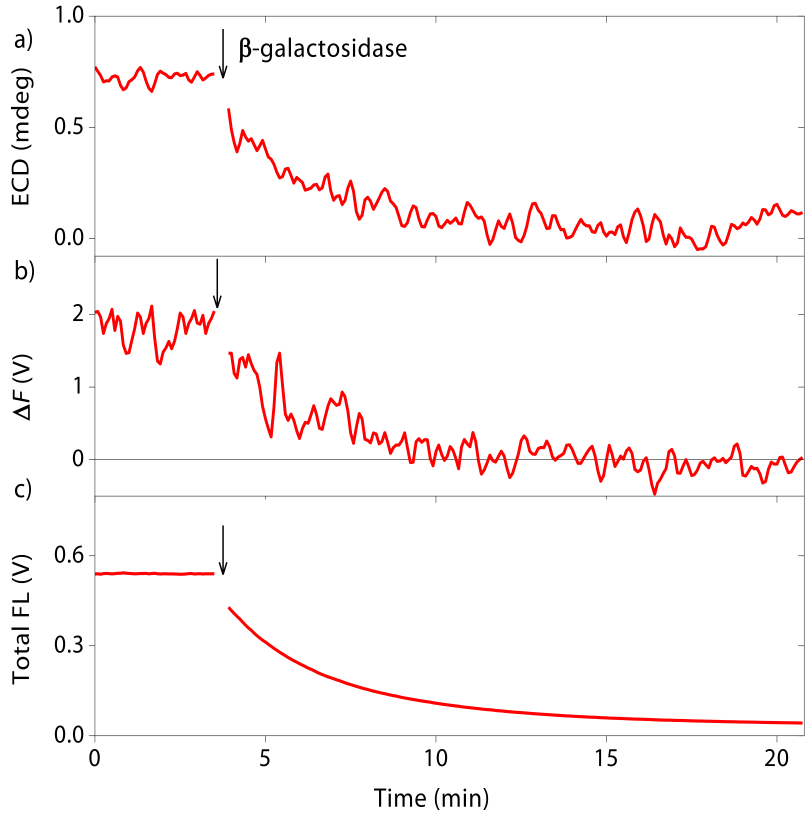

Figure S 34: Time Course (a) ECD, (b) Δ*F* and (c) total fluorescence (or DC voltage) measurements of **CB8•MDPP** (10 µM) in the presence of phenyl-β-D-galactopyranoside (50 µM) in 10 mM phosphate buffer at pH 5 upon addition of β-galactosidase (43 µg/ml). Parameters used: HT = 550V, BW = 4nm, Acc = 20, LP-Filter = 515 nm, λ_{obs} = 350 nm, Data Pitch = 5s, D.I.T = 8s, $t_{measure}$ = 21 min, T = 25°C

8.2. Uncovering of hidden aggregation phenomena by FDCD

8.2.1. Analyte spectra with MT receptor

Chiroptical spectroscopy can in some cases show erroneous effects if the system undergoes self-aggregation (especially at higher concentrations) giving rise to chiral signals even for achiral molecules.[29] This situation was encountered when studying the direct binding of the chiral epoxides ((1*R*,2*R*)-1-phenylpropylene oxide and (1*S*,2*S*)-1-phenylpropylene oxide) with the achiral molecular tube (**MT**) receptor. The **MT** receptor has been reported to strongly bind these chiral epoxides in water with K_a = 8.97 x 10^4 M^{-1} and exhibit strong induced ECD signals at a receptor and analyte concentration range of 100 µM and 500 µM respectively.[30] Likewise, the **MT** receptor shows induced Δ*F* and FDCD signal in the negative direction on addition of (1*R*,2*R*)-1-phenylpropylene oxide and in the positive direction on addition of (1*S*,2*S*)-1-phenylpropylene oxide (Figure S 35). This is also accompanied by a strong enhancement in the fluorescence intensity of the receptor molecule in both the cases (Figure S 35 (c)). The strong difference in the signal magnitudes of the induced ECD and FDCD signals was however not expected. Both Δ*F* as well as FDCD values can be used here (as measurements were conducted at the same HT voltage for both the analytes). The achiral **MT** receptor alone shows some signals near the excitation peak region of the receptor in both the Δ*F* as well as FDCD spectra, which can be an indication of photoselection artefacts (for further details refer Section 9.1).

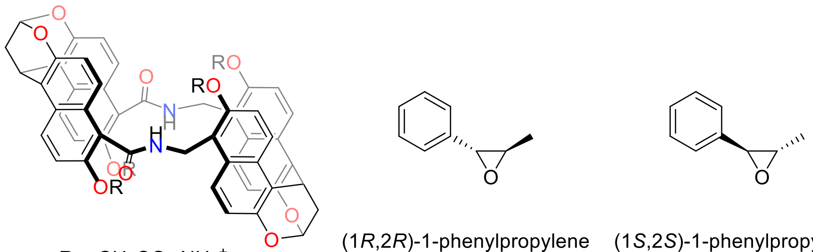

R = $CH_2CO_2^{-}NH_4^{+}$

MT

(1*R*,2*R*)-1-phenylpropylene oxide

(1*S*,2*S*)-1-phenylpropylene oxide

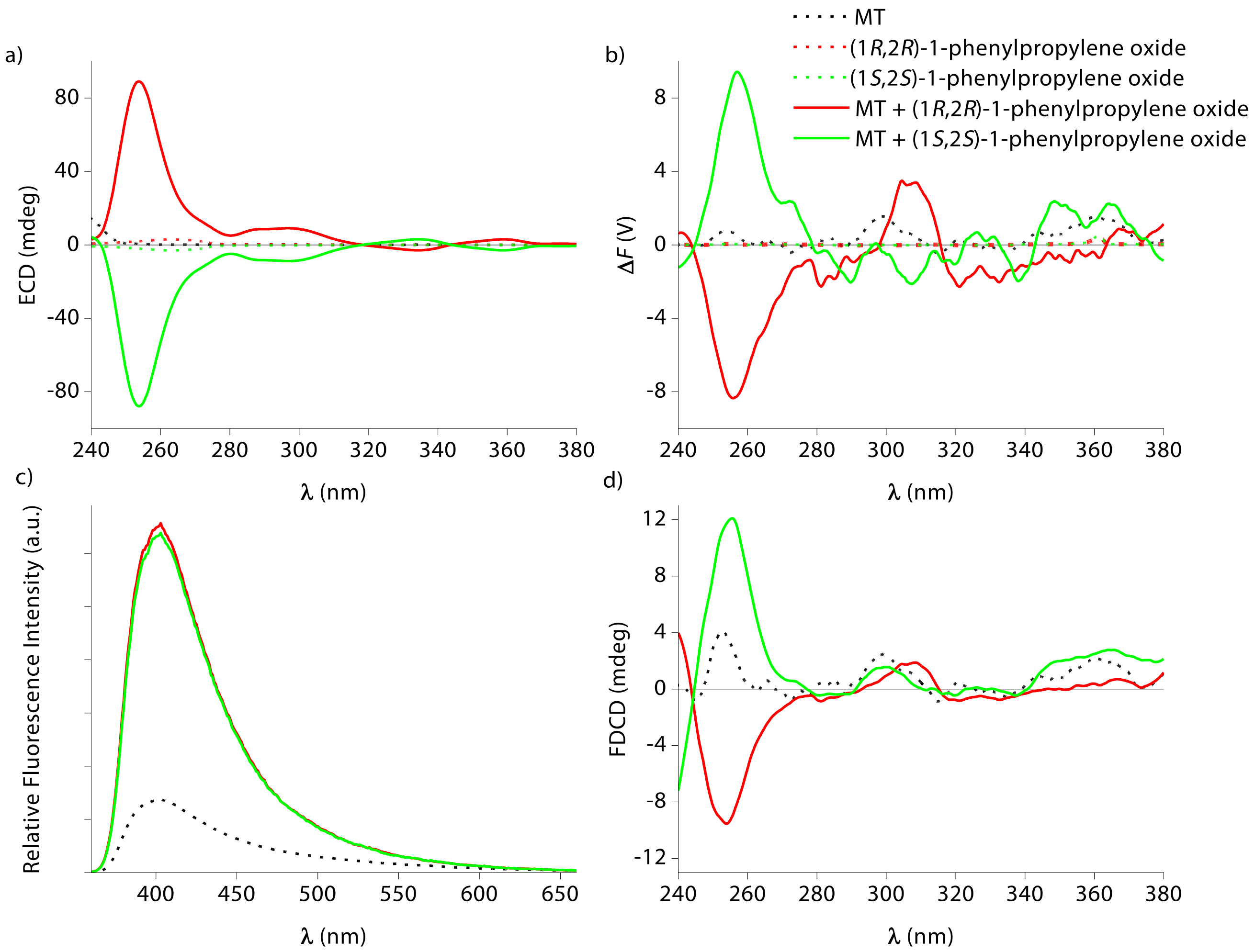

Figure S 35: (a) ECD and (b) ΔF spectra of freshly prepared **MT** receptor (100 µM) in the presence of (1*R*,2*R*)-1-phenylpropylene-oxide (500 µM) and (1*S*,2*S*)-1-phenylpropylene-oxide (500 µM) in DI-Water. Parameters used: HT = 630 V, BW = 4 nm, Acc = 20, LP-Filter = 380 nm, T = 25°C. (c) Enhancement in the fluorescence intensity of freshly prepared **MT** receptor (100 µM) upon addition of (1*R*,2*R*)-1-phenylpropylene-oxide (500 µM) and (1*S*,2*S*) -1-phenylpropylene-oxide (500 µM) in DI-Water, λ_{exc} = 340 nm. (d) FDCD spectra of freshly prepared **MT** receptor (100 µM) in the presence of (1*R*,2*R*)-1-phenylpropylene-oxide (500 µM) and (1*S*,2*S*) -1-phenylpropylene-oxide (500 µM).

When probing lower analyte concentrations, a completely unexpected inversion in the direction of ΔF and FDCD signal was observed (positive FDCD signal on addition of (1*R*,2*R*)-1-phenylpropylene oxide (Figure S 36) and negative FDCD signal on addition of (1*S*,2*S*)-1-phenylpropylene oxide (Figure S 37)), while the sign of the ECD signal remained unchanged. The epoxides were always present in excess when compared to the receptor in all the cases. The degree of complexation of the **MT**•receptor at **MT** : epoxide concentration of 100 : 500 µM, 2.5 : 10 µM and 1.25 : 5 µM varies from 97.3% to 44.4% and 29.4% respectively upon dilution. However, even though the amount of **MT** receptor complexed decreases upon dilution, an inversion in the direction of ΔF and FDCD signal was still not expected.

Moreover, the FDCD signal should be concentration independent, which holds true for the spectra measured at lower concentration (**MT** : epoxide concentration of 2.5 : 10 µM and 1.25 : 5 µM), while the inversion in the FDCD signal direction at higher concentration indicates the influence of other effects. The parameters used for the concentration dependent study for both (1*R*,2*R*)-1-phenylpropylene oxide and (1*S*,2*S*)-1-phenylpropylene oxide is given in Table S 10.

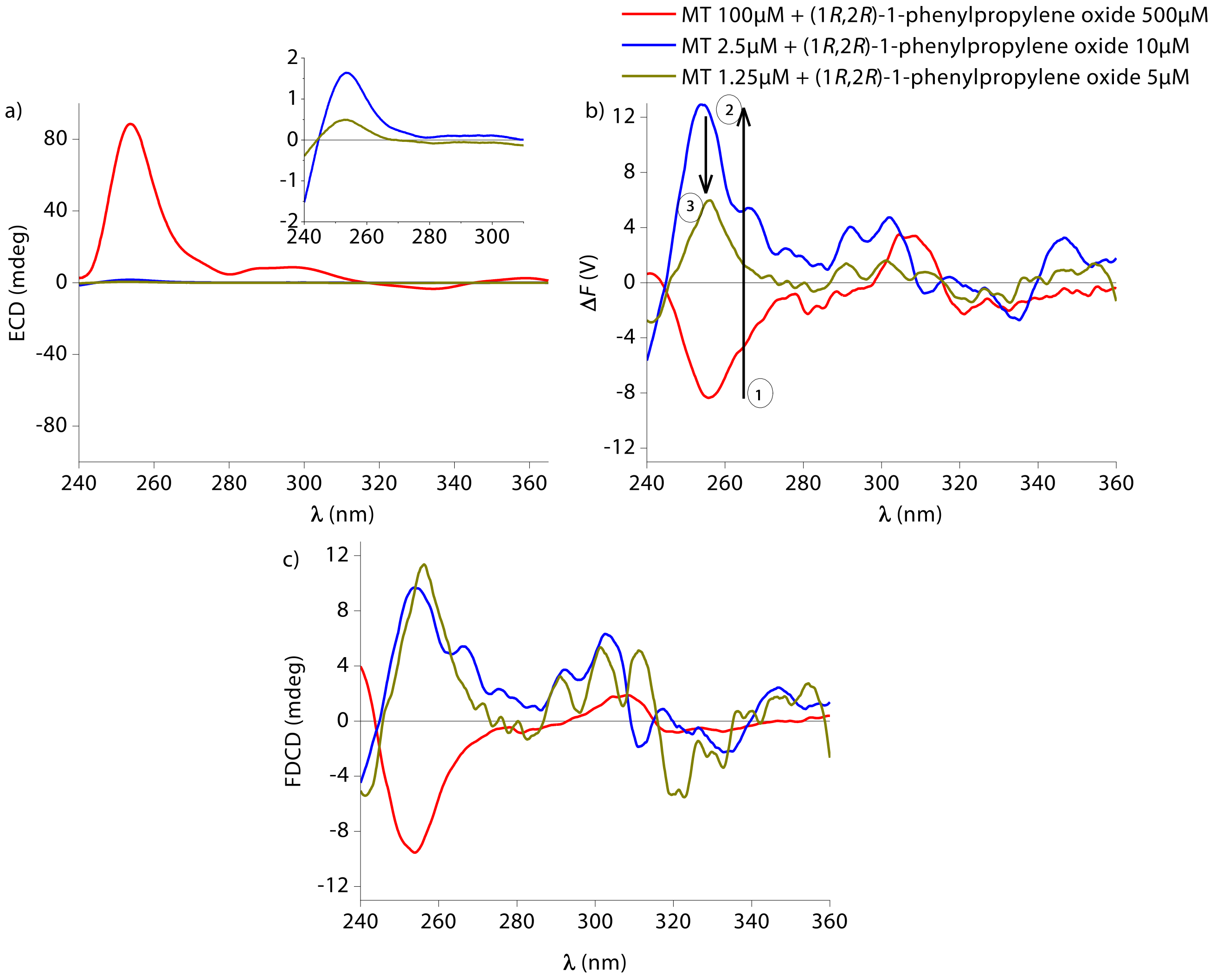

Figure S 36: Concentration dependent (a) ECD, (b) Δ*F* and (c) FDCD spectra of freshly prepared **MT** receptor in presence of (1*R*,2*R*)-1-phenylpropylene-oxide in DI-Water. The inset in the ECD spectra shows the zoomed in signals in the 240 nm to 310 nm region. The parameters were kept constant for each individual set of measurements and are given in the Table S 10. The arrows indicate the direction of dilution. An inversion in the direction of Δ*F* and FDCD signal was observed on going to lower concentrations.

Table S 10: Parameters used to obtain the concentration dependent ECD, Δ*F* and FDCD spectra of **MT** receptor in presence of both (1*R*,2*R*)-1-phenylpropylene-oxide and (1*S*,2*S*)-1-phenylpropylene-oxide shown in Figure S 36 and Figure S 37.

Sample[a]	***HT***[b] ***(V)***
**MT 100 µM + epoxide 500 µM**	630
**MT 2.5 µM + epoxide 10 µM**	800
**MT 1.25 µM + epoxide 5 µM**	800

a) Measured at BW = 4 nm, Acc = 20, LP-Filter = 380 nm; b) HT Voltage applied to the PMT of the FDCD detector. Hence this is only influencing the Δ*F* value and not the ECD value (different detectors)

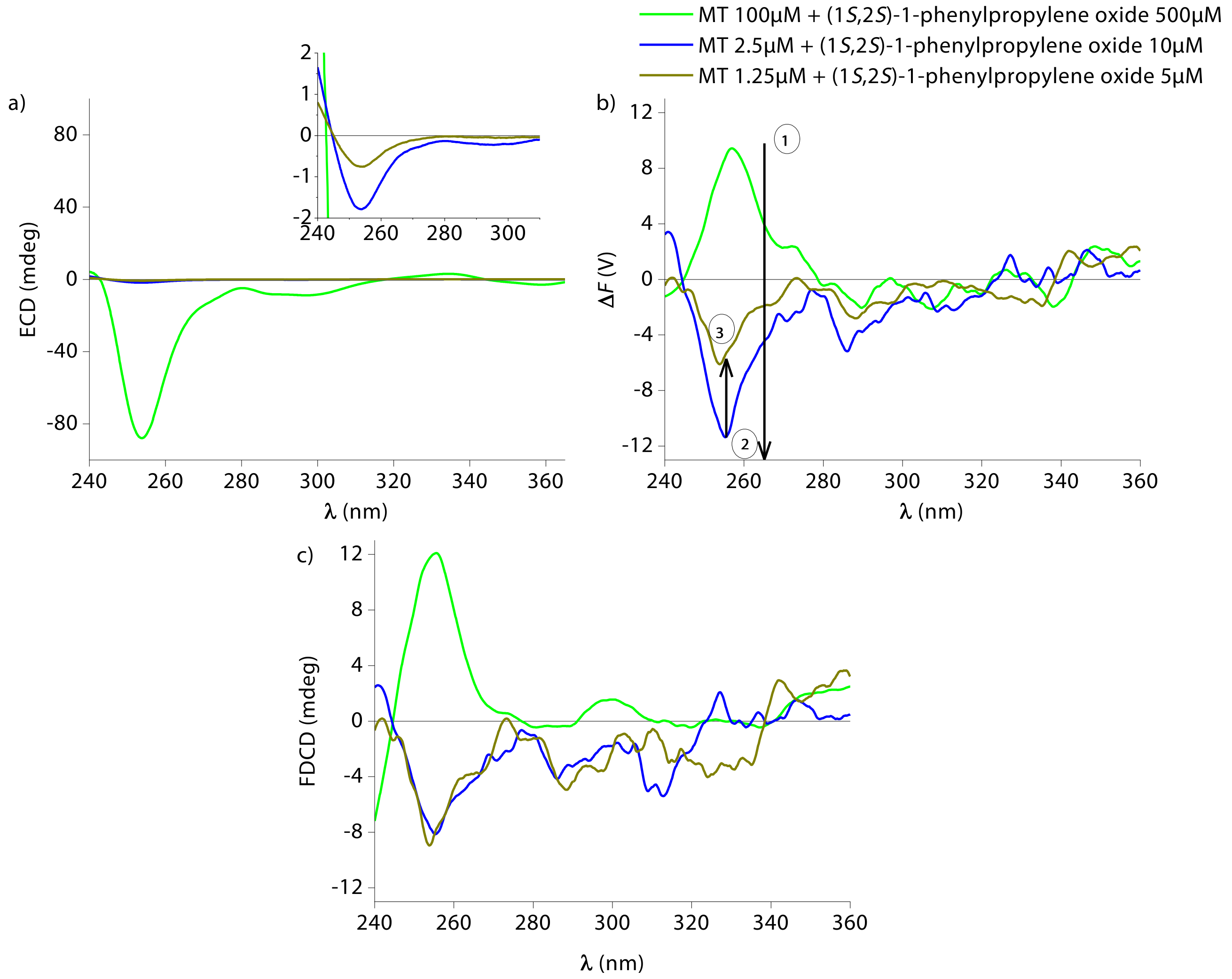

Figure S 37: Concentration dependent (a) ECD, (b) Δ*F* and (c) FDCD spectra of freshly prepared **MT** receptor in presence of (1*S*,2*S*)-1-phenylpropylene-oxide in DI-Water. The inset in the ECD spectra shows the zoomed in signals in the 240 nm to 310 nm region. The parameters were kept constant for each individual set of measurements and are given in Table S 10. The arrows indicate the direction of dilution. An inversion in the direction of Δ*F* and FDCD signal was observed on going to lower concentrations.

The molar circular dichroism values were calculated from both the ECD and FDCD ellipticity values for spectra collected at the lower concentrations (Figure S 38).

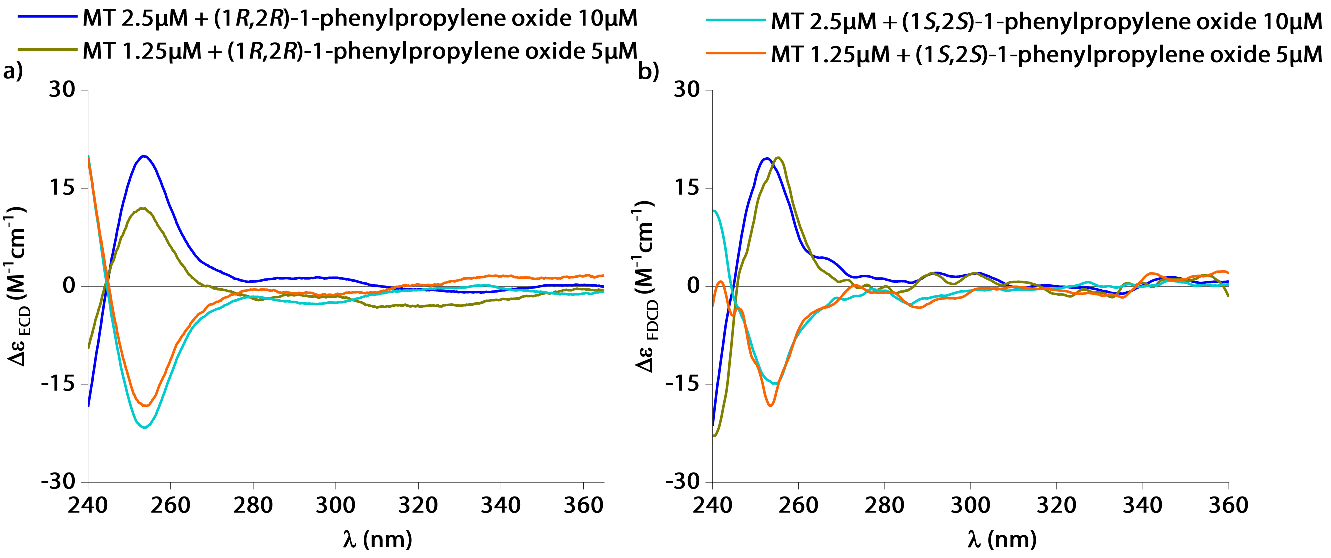

Figure S 38: $\Delta\varepsilon_{ECD}$ and $\Delta\varepsilon_{FDCD}$ spectra of freshly prepared **MT** receptor in presence of (1*R*,2*R*)-1-phenylpropylene-oxide and (1*S*,2*S*)-1-phenylpropylene-oxide in DI-Water at varying concentrations (**MT** : epoxide - (2.5 : 10 µM and 1.25 : 5 µM) calculated from ECD and FDCD ellipticity values using Eq. S3-S5.

In order to check the influence of photoselection artefacts or anisotropy of the system in the measured ECD and FDCD spectra at higher concentrations, the linear polarization components, i.e. LD and LB in case of ECD, and FDLD in case of FDCD were investigated for the achiral **MT** receptor and the **MT**•(1*R*,2*R*)-1-phenylpropylene oxide complex at the receptor concentration of 100 µM (Figure S 39 and Figure S 40). Both the LD and FDLD spectra were measured simultaneously to the ECD and FDCD spectra. The LB spectra were measured on a JASCO J-1500 CD spectrometer at a JASCO facility in Pfungstadt by placing a Glan-Taylor polarizer behind the sample at 45° into the beam path. In the absence of anisotropy and signal artefacts, the LD and FDLD spectrum should not give any signal. Concerning LB measurements, factory simulations from the instrument manufacturer indicate that artifacts induced in ECD spectra from LB components have a ratio intensity 1/100 in respect to LB measured values (thus, a signal strength of 200 mdeg measured on the LB spectra corresponds to an artefact of 1 mdeg in the ECD spectra). Furthermore, the fluorescence excitation anisotropy of the system was measured using a fluorescence spectrometer equipped with automatic polarizers to obtain supplementary information about the sample's properties and to check the influence of photoselection artefacts in FDCD measurements (Figure S 40 (c)).

In Figure S 39, the measured LD and LB values reveals that they are suitably small and will not strongly influence the ECD signal in case of the **MT** and the **MT**•(1*R*,2*R*)-1-phenylpropylene oxide complex. The LD and LB signals observed below 250 nm can be due to the high absorbance of the sample in the lower wavelength region and does not seem to coincide with the ECD signal peak. However, the FDLD spectra measured for this system shows strong signals in the region where we observe the apparent FDCD bands from the achiral **MT** receptor (Figure S 40). Hence, the measured FDCD signals are apparent values and has strong contributions from the photoselection artefacts. The excitation fluorescence anisotropy spectra measured also reveals the influence of strong fluorescence anisotropy in the system (Figure S 40 (c)). In addition, the FDCD spectra of the achiral **MT** receptor alone now shows stronger signals (Figure S 40 (a)) as compared to the spectra measured using a freshly prepared **MT** stock solution in Figure S 35 (d). Hence, this can be an indication of aggregation of the achiral **MT** receptor with time, resulting in strong photoselection artefacts in the measured FDCD spectra.

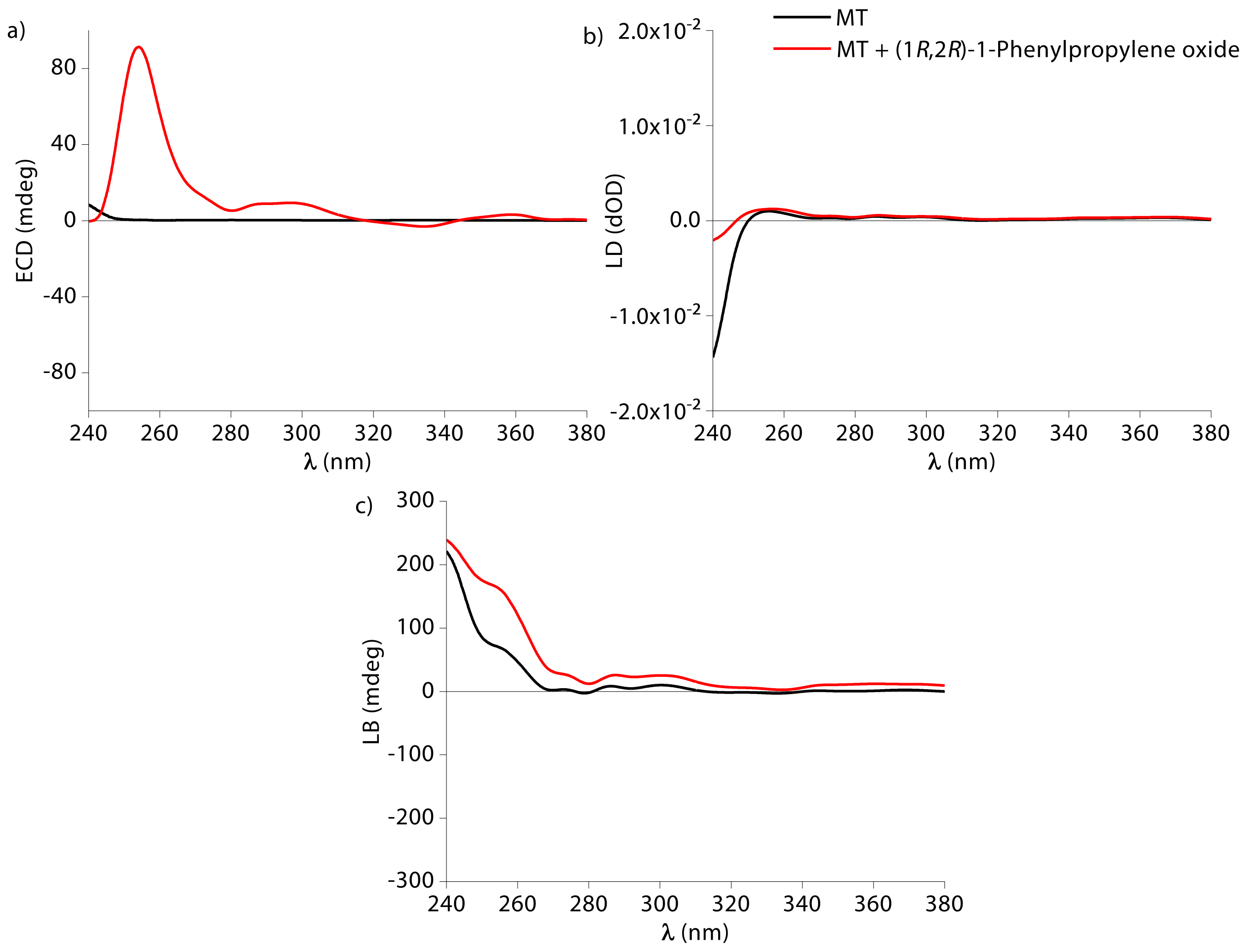

Figure S 39: a) ECD, (b) LD and (c) LB spectra of **MT** receptor (100 µM) in the absence and presence of (1*R*,2*R*)-1-phenylpropylene-oxide (500 µM) in DI-Water. Parameters used : BW = 4 nm, Acc = 20, T = 25°C. (The LB spectra were measured on a JASCO J-1500 CD spectrometer at a JASCO facility in Pfungstadt by placing a Glan-Taylor polarizer behind the sample at 45° into the beam path. Concerning LB measurements, factory simulations from the instrument manufacturer indicate that artifacts induced in ECD spectra from LB components have a ratio intensity 1/100 in respect to LB measured values. Thus, a signal strength of 200 mdeg measured on the LB spectra corresponds to an artefact of 1 mdeg in the ECD spectra.)

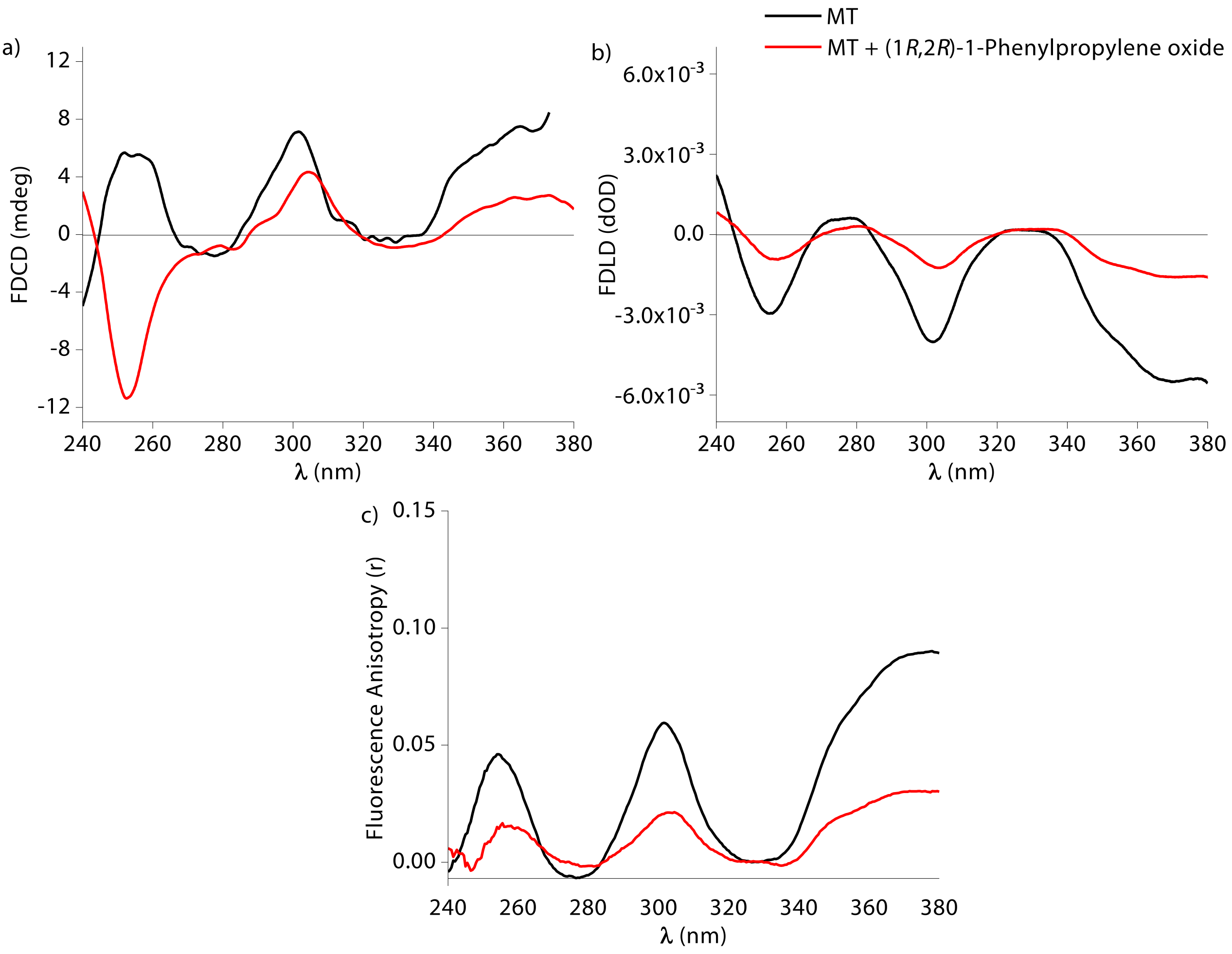

Figure S 40: a) FDCD and (b) FDLD spectra of **MT** receptor (100 µM) in the absence and presence of (1*R*,2*R*)-1-phenylpropylene-oxide (500 µM) in DI-Water. Parameters used: HT = 570 V, BW = 4 nm, Acc = 20, LP-Filter = 380 nm, T = 25°C. (c) Fluorescence excitation anisotropy spectra of **MT** receptor (100 µM) in the absence and presence of (1*R*,2*R*)-1-phenylpropylene-oxide (500 µM) in DI-Water. Parameters used: λ_{ems} = 450 nm, T = 25°C.

For comparison to the above results, the presence of photoselection artefacts was also monitored by measuring the LD and FDLD spectra together with the ECD and FDCD spectra in case of 0.0024 M ammonium d-10-camphorsulfonic acid (d-10-ACS) (Figure S 41) as the reference standard and for the **CB8•MDPP•**L-Phe (Figure S 42) host-guest system. In case of d-10-ACS and the **CB8•MDPP•**L-Phe host-guest system, the signal intensity obtained in LD and FDLD mode are small, indicating no significant influence from fluorescence anisotropy or photoselection artefacts in the measured ECD and FDCD spectra. Hence, this further confirms at our spectrometer that in the absence of photoselection artefacts, the LD and FDLD spectra will not show any signals.

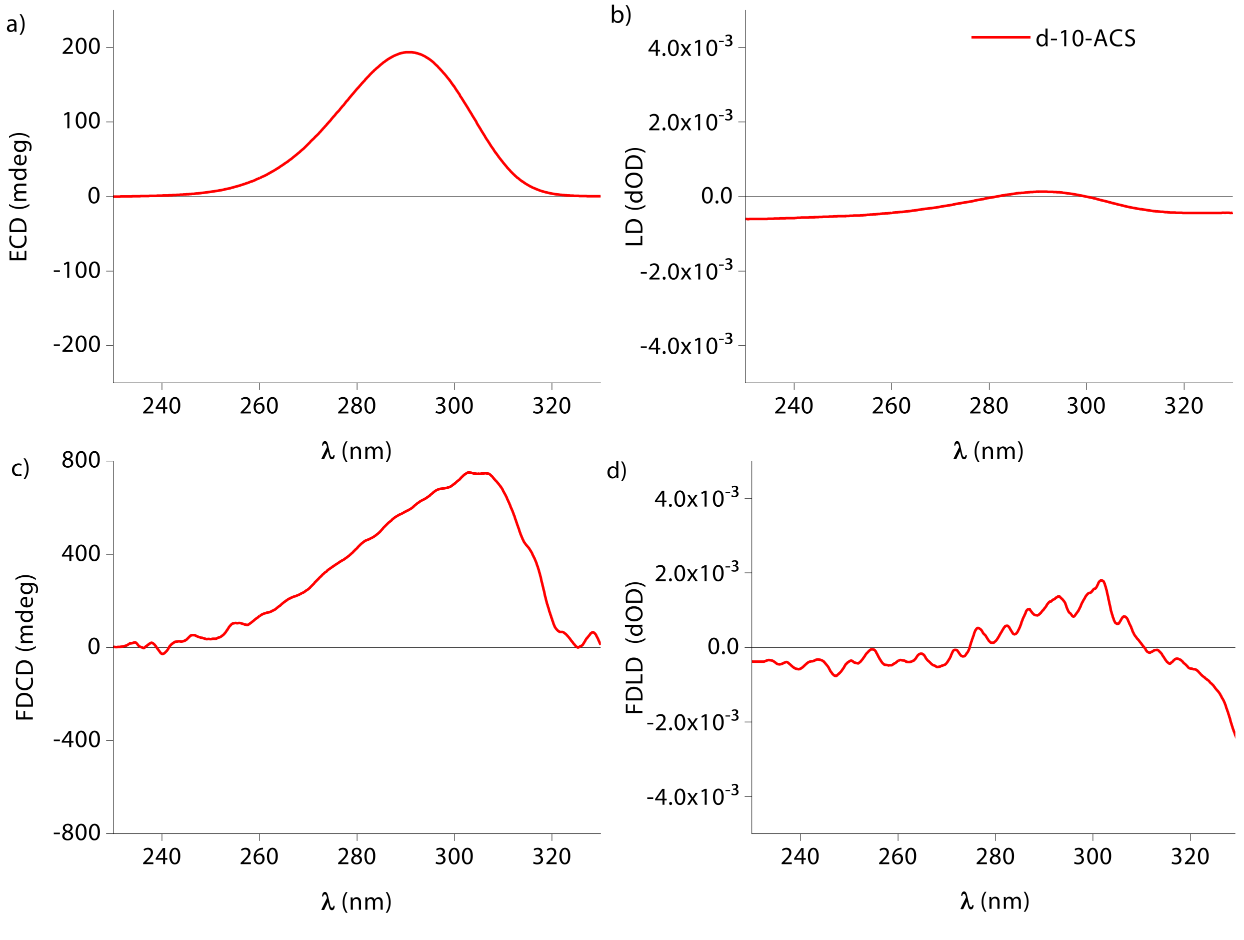

Figure S 41: (a) ECD, (b) LD, (c) FDCD and (d) FDLD spectra of 0.0024M d-10-ACS in DI-Water. Parameters used: HT = 840 V, BW = 4 nm, Acc = 20, LP-Filter = 380 nm, T = 25°C.

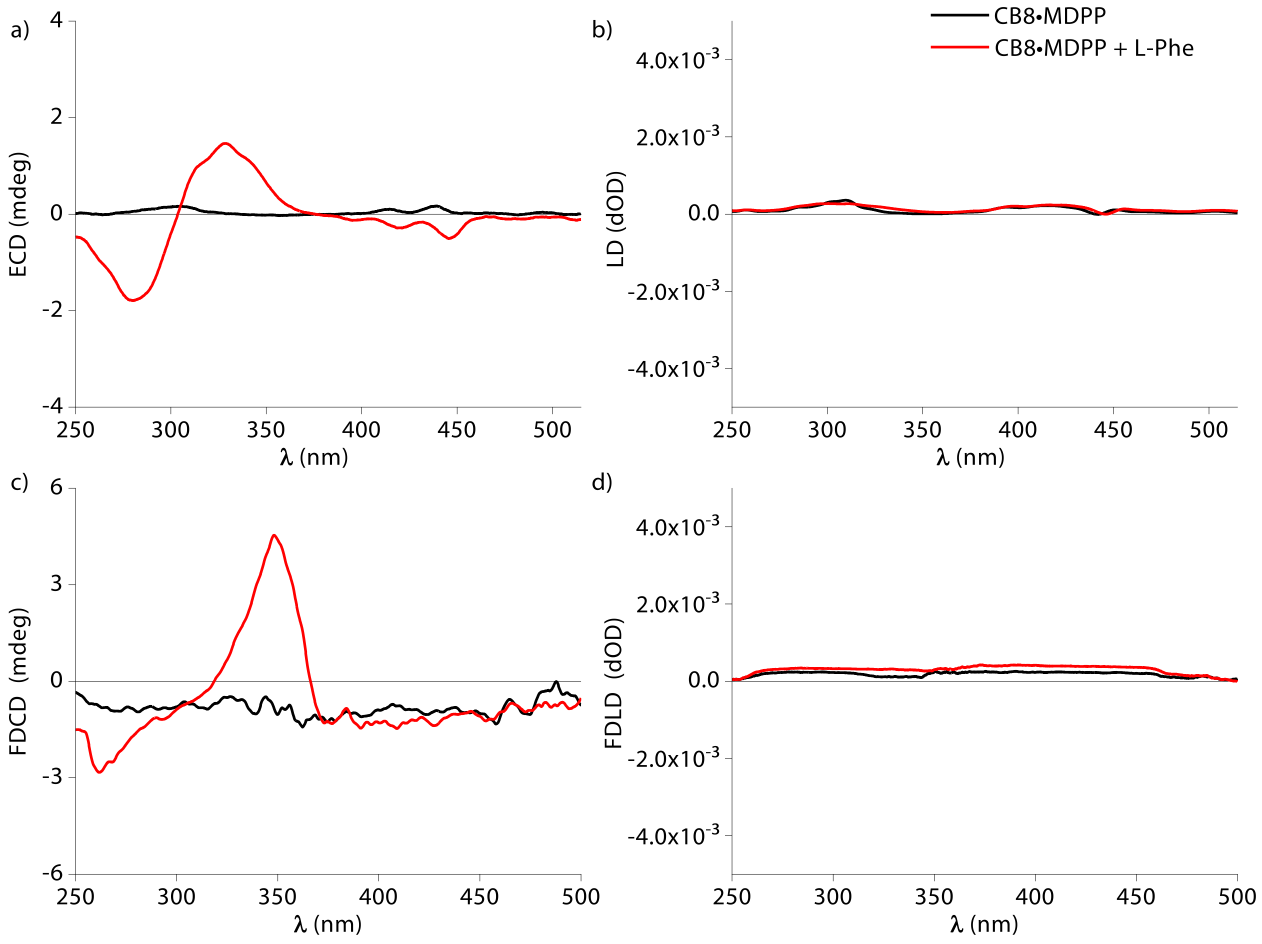

Figure S 42: (a) ECD, (b) LD, (c) FDCD and (d) FDLD spectra of **CB8•MDPP** (20 μM) in the absence and presence of L-Phe (50 μM) in DI-Water. Parameters used: HT = 470 V, BW = 4 nm, Acc = 20, LP-Filter = 515 nm, T = 25°C.

The ECD and FDCD signals were also collected for the **MT**•(1*R*,2*R*)-1-phenylpropylene oxide complex at the receptor concentration of 100 µM using an *artefact-free* FDCD spectrometer set-up (Figure S 43). The results resembled the FDCD data collected at lower concentrations of host and guest at the standard FDCD spectrometer used in our study (Figure S 36). Hence, it has to be concluded that something unusual happens at higher receptor and analyte concentrations, which was previously hidden in the ECD spectra. We now believe that the host•guest complexes form self-aggregating structures, and thus that the chiroptical properties measured by ECD and FDCD have both contributions from the chiral host-guest complex itself, and also from the chiral superstructures and strong photoselection artefacts upon aggregation.

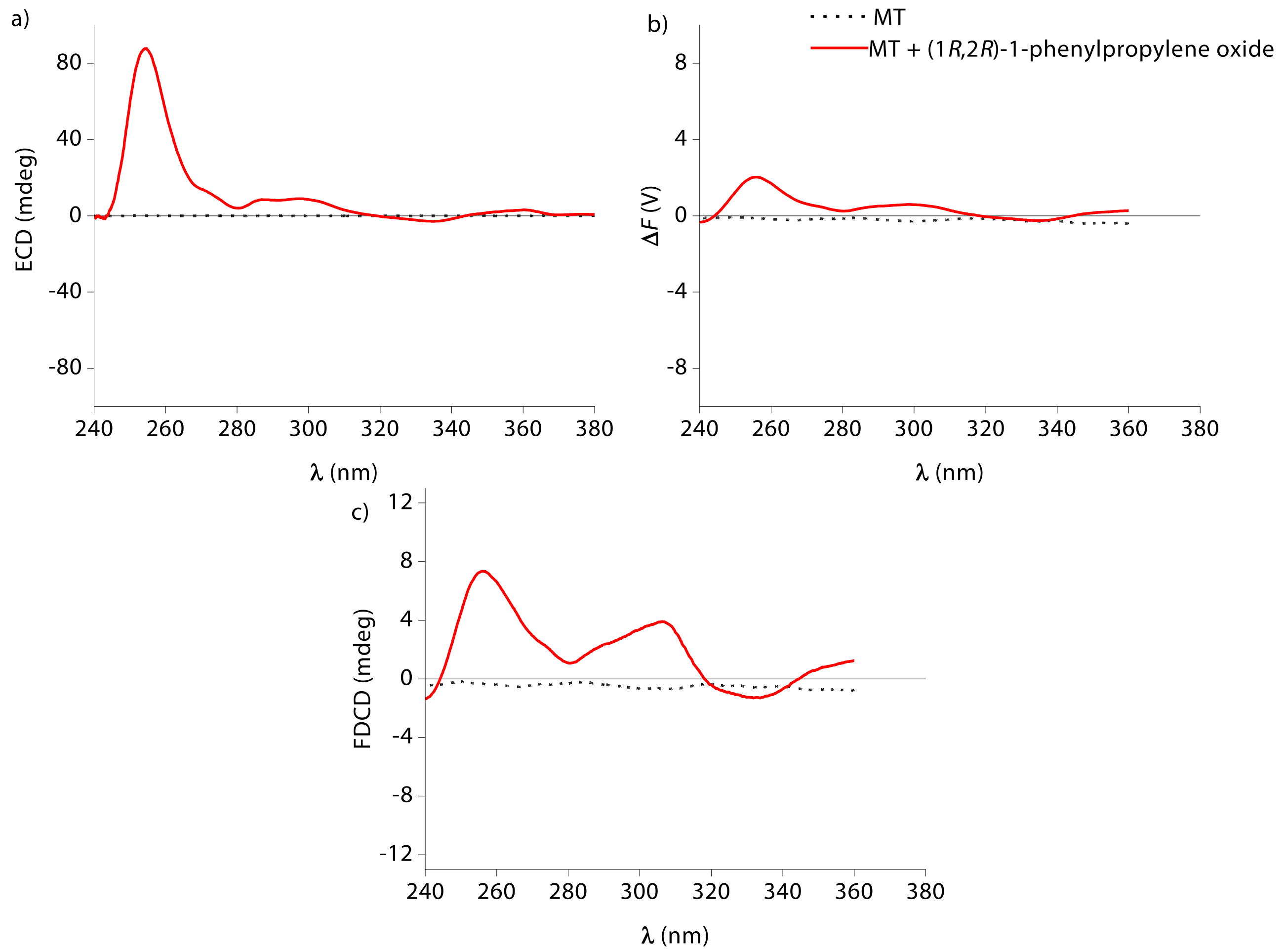

Figure S 43: (a) ECD and (b) Δ*F* spectra of **MT** receptor (100 µM) in the presence of (1*R*,2*R*)-1-phenylpropylene-oxide (500 µM) in DI-Water using artefact-free 551-FDCD spectrometer, measured in Japan. Parameters used: HT = 350 V (for **MT** receptor) and 300 V (for **MT** + (1*R*,2*R*)-1-phenylpropylene-oxide), BW = 4 nm, Acc = 20, LP-Filter = 380 nm, T = 25°C, 2.5 mm balancing mask was used for preventing the influence from fluorescence anisotropy. (c) FDCD spectra of **MT** receptor (100 µM) in the presence of (1*R*,2*R*)-1-phenylpropylene-oxide (500 µM).

To study this unexpected inversion in the direction of FDCD signal, the **MT** receptor was monitored to see if it undergoes aggregation with time. The aggregation studies of the **MT** receptor are discussed in section 8.2.2.

8.2.2. Aggregation Studies of MT receptor

To study the aggregation property of **MT** receptor, the ECD and FDCD spectra of the **MT** receptor was monitored over time. Initially a 1.0 mM stock solution of the **MT** receptor was freshly prepared and the ECD and FDCD spectra of a 100 µM solution prepared from this freshly prepared stock solution was measured at 25°C. The 1.0 mM stock solution was then kept aside in a sealed vial and stored in the fridge for 1 day. Following this, the ECD and FDCD spectra of a 100 µM solution prepared from the one day old 1.0 mM stock solution was also measured at 25°C. This shows an emerging FDCD band at 300 nm and 360 nm with time for the achiral **MT** receptor (Figure S 44), which is an indication that the receptor is undergoing aggregation over time in the 1.0 mM stock solution, which is still present at a concentration of 100 µM. The apparent chiral FDCD signals observed on aggregation of the **MT** receptor molecule can be attributed to the anisotropic excitation, also known as photoselection, and from instrument-related artefacts (for further details refer Section 9.1). Hence, FDCD can be used to obtain useful information in case of such complex supramolecular systems, which may not be obvious when using ECD and fluorescence spectroscopy alone.

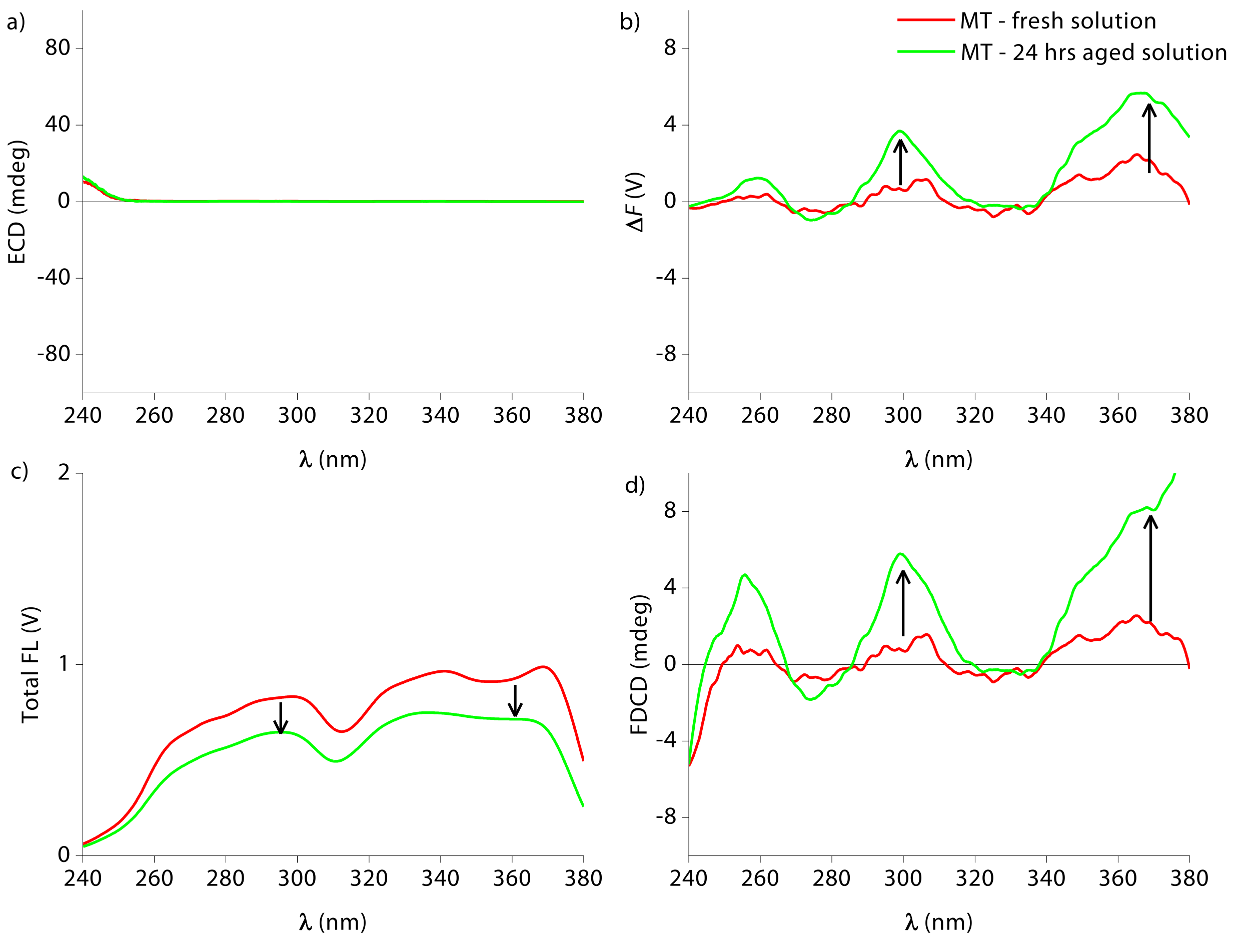

Figure S 44: (a) ECD, (b) Δ*F*, (c) total fluorescence (or DC voltage) and (d) FDCD spectra of a 100 µM **MT** receptor solution prepared from a freshly prepared 1.0 mM stock solution in DI-Water (red line) and after keeping the 1.0 mM stock solution for 24 hours (green line). The emerging FDCD band at 300 nm and 360 nm can be an indication that the host molecule is undergoing aggregation over time in the 1.0 mM stock solution which is still present at a concentration of 100 µM. The total fluorescence intensity collected in the DC channel also shows a reduction in the fluorescence intensity upon aggregation. Parameters used: HT = 630 V, BW = 4 nm, Acc = 20, LP-Filter = 380 nm, T = 25°C.

The Δ*F* and FDCD signal arising at 300 nm in the **MT** receptor was also monitored by increasing the temperature from 5ºC to 60ºC followed by cooling the solution back from 60ºC to 5ºC (Figure S 45). This shows a strong decrease in the Δ*F* and FDCD signal at higher temperature, which in turn is an indication that the **MT** receptor aggregates dissolve when going to higher temperatures. The aggregation/deaggregation process is reversible as can be seen by the enhancement in the Δ*F* and FDCD signal (going back to the initial value at 300 nm) when cooling the solution back from 60ºC to 5ºC. It should also be noted that the photoselection effects/fluorescence anisotropy also decreases with increase in temperature mainly because the viscosity of the media decreases with increase in temperature and *vice versa*.[31] Hence this effect is also seen here in the temperature dependent graph (Figure S 45).

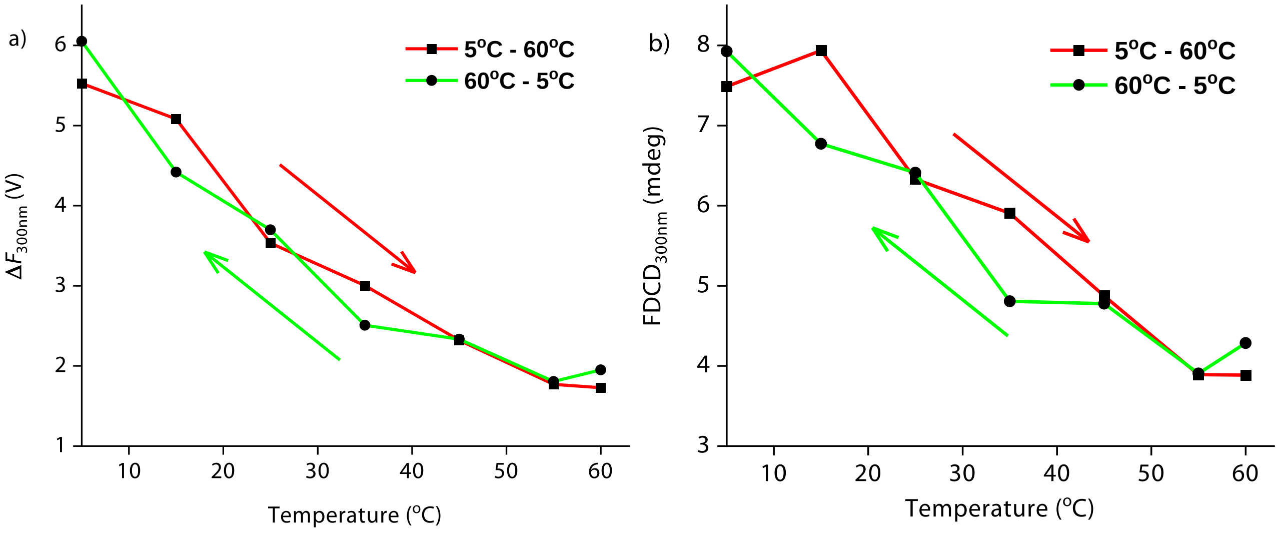

Figure S 45: (a) Δ*F* and (b) FDCD signal at 300 nm of the **MT** receptor (100 µM) in DI-Water on increasing the temperature from 5ºC to 60ºC (red line) and on cooling the solution back from 60ºC to 5ºC (green line)

The possibility of aggregation was also studied by collecting the emission intensity of the receptor molecule at 403 nm upon increasing the concentration from 1.56 μM to 100 μM (Figure S 46 (a)). The excitation wavelength was accordingly selected such that the absorbance of the 100 μM **MT** solution at the exciting wavelength is less than 0.1 in all experiments, excluding any inner filter effects. According to theory, in this case the fluorescence intensity collected at 403 nm should follow a straight line with increasing concentration (considering that there is no inner filter effects) if the fluorophore remains in its fully dissolved, non-aggregated state. However, a deviation in linearity at higher concentrations was observed for **MT** receptor, suggesting that it undergoes self-aggregation at higher concentrations. This phenomenon was also checked in the case of the emissive compound, *R*-propanolol hydrochloride as the reference in 10 mM phosphate buffer at pH 7. *R*-propanolol hydrochloride has a similar stokes shift as the **MT** receptor molecule. Upon collecting the emission intensity of *R*-propanolol hydrochloride at 344 nm upon increasing the concentration from 1.5 μM to 100 μM, the fluorescence intensity follows a straight line with increasing concentration, as is expected in the absence of aggregation (Figure S 46 (b)). Here again care was taken such that the absorbance of the 100 μM *R*-propanolol hydrochloride solution at the exciting wavelength is less than 0.1, excluding any inner filter effects.

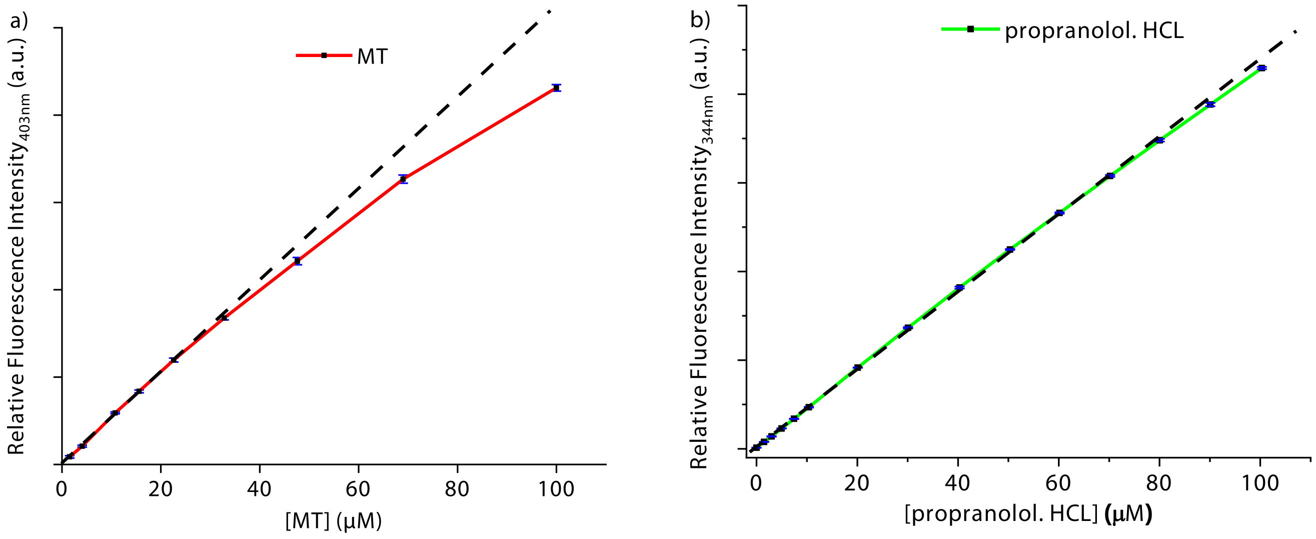

Figure S 46: (a) Emission intensity collected at 403 nm upon increasing the concentration of **MT** receptor from 1.56 μM to 100 μM in DI-Water. λ_{exc} = 383 nm. The slight deviation in linearity can be an indication of their aggregation at higher concentration. (b) Emission intensity collected at 344 nm upon increasing the concentration of *R*-propanolol hydrochloride from 1.5 μM to 100 μM in DI-Water. λ_{exc} = 322 nm. The vertical error bars for the fluorescence intensity values are depicted in blue in the graph.

The aggregation phenomena of **MT** receptor molecule was also studied by making use of DOSY NMR, which is known to be more sensitive to aggregation phenomena than ^{1}H NMR. Hence, the DOSY NMR spectra of the **MT** receptor in D_2O was collected at different concentrations. The DOSY NMR spectrum depicted in Figure S 47 shows that the host diffuses slower at higher concentrations, which again confirms that the **MT** receptor molecule can form aggregates in solution.

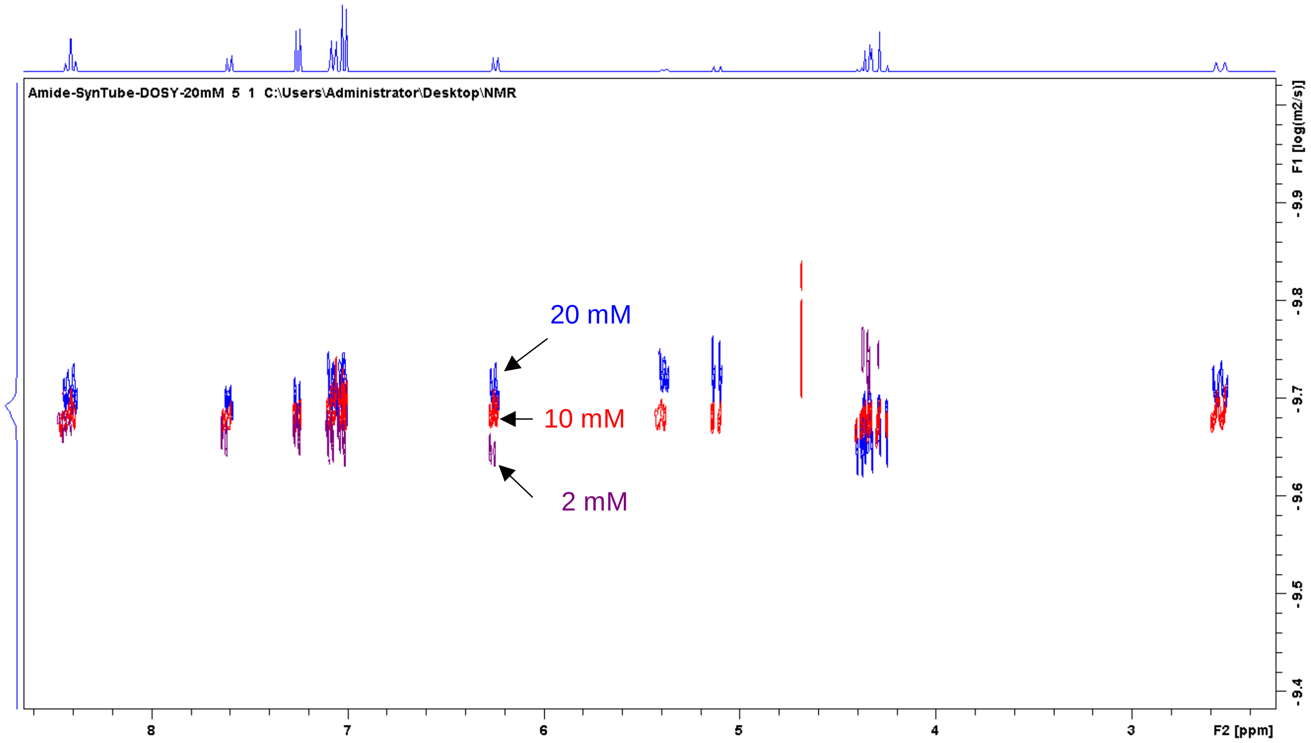

Figure S 47: DOSY NMR spectrum of **MT** receptor at 2 mM, 10 mM and 20 mM in D_2O.

In order to further confirm the possibility of aggregation and to characterize the size of the aggregates, Dynamic Light Scattering (DLS) was also performed.[32] Figure S 48 (a, b) represents the acquired data as both intensity size distribution and volume size distribution for the **MT** receptor solution (100 μM) prepared in DI-Water and filtered using a polyethersulfone membrane with a pore size of 0.43 μm. Both distributions show a predominant peak with a hydrodynamic diameter of 60.92 nm as well as a minor peak with a hydrodynamic diameter of 1752 nm corresponding to some bigger aggregates. The sharp peak at 60.92 nm has a polydispersity index (PDI)[33] of 0.165. Given the size of **MT** receptor is on the order of 1 nm, it is clear that the peak at ~60 nm corresponds to aggregates.

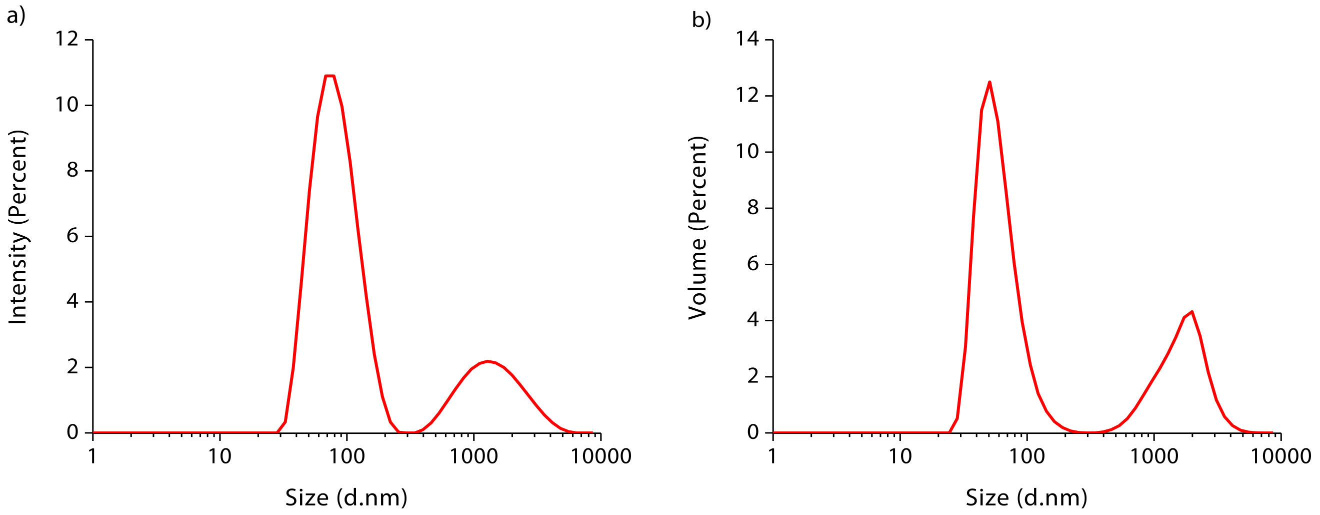

Figure S 48: DLS measurements showing (a) intensity distribution versus particle diameter and (b) volume distribution versus particle diameter of **MT** receptor solution (100 μM) in DI-Water. Parameters used: T = 25°C, material RI = 1.45, dispersant RI = 1.330, viscosity = 0.8872 cP, measurement position = 4.65 mm, attenuator = 11.

Comparing the DLS results obtained at 100 μM of **MT** receptor (Figure S 48) to the DOSY NMR spectra (Figure S 47), it appears that higher concentrations are needed to influence the DOSY signal. This can be true because NMR is operating on a different time scale and in general, yields an averaged diffusion coefficient among the fast exchanging aggregates of different sizes.[34] Hence, it may well be that the aggregates formed at ~100 μM of **MT** receptor are still fleeting in nature and diffuse relatively freely, while optically-probed properties (like ECD, FDCD, fluorescence) as well as DLS provide the instant snap shot, and hence displays several distinct species, according to the aggregate size.[34]

8.3. FDCD measurements for background reduction in complex systems and chromophoric biofluids

8.3.1. Detection of insulin by CB8•MDPP receptor

The induced FDCD and ECD signals arising in the **CB8•MDPP** receptor in presence of chiral biological analytes was used for the detection of insulin, which is known to bind to **CB8•MDPP**.[10] The receptor shows both induced ECD, Δ*F* and FDCD signals in presence of insulin in 10 mM phosphate buffer at pH 2.7 (Figure S 49). The dashed red line represents the ECD and Δ*F* signals arising from insulin alone. The ECD spectrum of the **CB8•MDPP•**insulin complex contains strong contributions arising from insulin backbone while the insulin-backbone does not contribute to the Δ*F* and FDCD spectrum. Both the Δ*F* as well as FDCD values can be used here for analysis. However, when using the Δ*F* signals, one should correct for the signal artefacts arising from the **CB8•MDPP** receptor alone (which overlaps with the measured signal), for further information refer to Section 9.1.1.

The Δ*F* and FDCD spectrum of the **CB8•MDPP•**insulin complex shows some broad peaks in the 375 nm to 475 nm region. To verify that this FDCD signal is a true signal and not an artefact arising from the instrument, **CB8•MDPP•**insulin receptor-analyte complex was destroyed through the addition of memantine, which sequesters **CB8** (Ka ≈ 10^{11} M^{-1}).[20] This lead to a disappearance of the induced Δ*F* and FDCD signal in the 375 nm to 475 nm region (Figure S 49, green line), which confirms that the FDCD signals observed was inherently due to the **CB8•MDPP•**insulin complex, and not caused by photoselection artefacts.

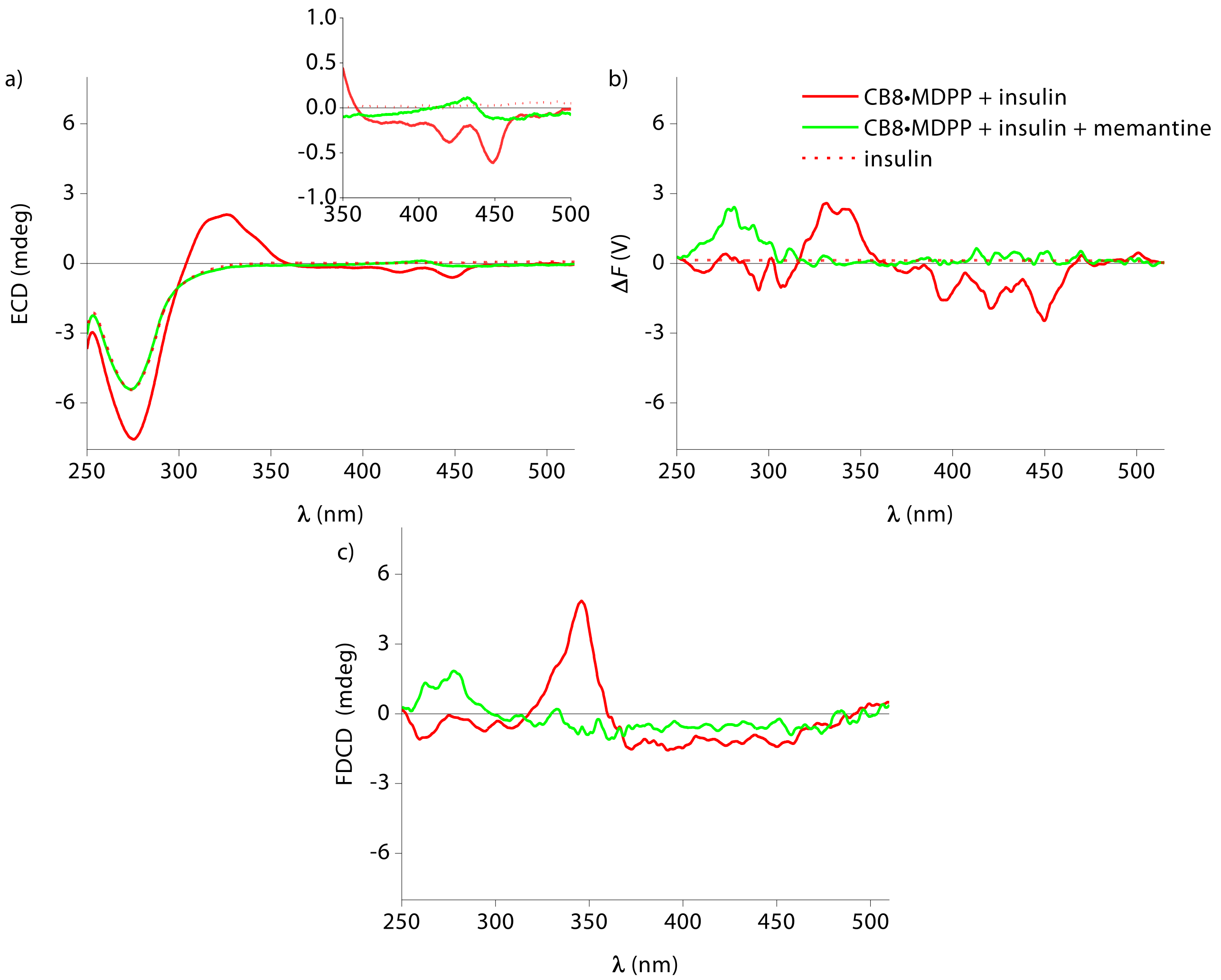

Figure S 49: (a) ECD, (b) Δ*F* and (c) FDCD spectra of **CB8•MDPP** (20 μM) in the presence of insulin (50 μM) (red line) and in the presence of both insulin (50 μM) and memantine (50 μM) (green line) in 10 mM phosphate buffer at pH 2.7. The red dashed line represents the ECD and Δ*F* signals arising from insulin (50 μM) alone. The inset shows the induced ECD signals in the 350 nm to 500 nm region. Parameters used: HT = 510 V, BW = 4 nm, Acc = 20, LP-Filter = 515 nm, T = 25°C

8.3.2. Detection of memantine in blood serum by CB8•MPCP and CB8•MVCP receptor

The development of simple methods for the accurate detection and quantification of analytes or drugs in blood serum play a significant role in many biochemical and clinical applications. Blood serum is a strongly chromophoric and turbid biofluid that contains chiral components. Hence, the direct spectroscopic detection of analytes in blood serum can be quite challenging. As was shown above, the two enantiomers of the chiral MPCP dye ((R_p)-MPCP and (S_p)-MPCP) form a 1:1 host-dye reporter pair with **CB8** that exhibits both induced ECD and Δ*F* signals in DI-Water (Figure S 22). The unbound dye (chiral and emissive) alone shows both ECD and Δ*F* signals even in the absence of **CB8**. However on addition of **CB8** only a slight shift in the ECD signals is observed in both the cases, while a strong enhancement in the Δ*F* signal was observed (Figure S 22). This stronger signal differences in Δ*F* than in ECD between **CB8** bound and free MPCP implies that host-guest binding commonly leads to more pronounced changes in the emission than the absorbance spectra, and thus one can generally expect to find larger signal differences in Δ*F* than in ECD. This feature can be advantageous for sensing applications that rely on signal differences. Hence, we tried to detect this ultra-high affinity **CB8**•MPCP host-guest pair in human blood serum (HS) and to follow the MPCP displacement in presence of Alzheimer's drug memantine, which competitively and strongly binds **CB8**, with the help of FDCD.

The HS sample was filtered using a polyethersulfone membrane with a pore size of 0.22 µm before the measurements. The spectrum of HS alone shows a strong positive ECD and Δ*F* background signal. The addition of 20 µM **CB8**•(R_p)-MPCP to the HS sample resulted in a change in both the ECD and Δ*F* spectra (ECD and Δ*F* signal becomes more negative) (Figure S 50). Similar changes in both the ECD and Δ*F* spectra were also observed on addition of 20 µM **CB8**•(S_p)-MPCP to the HS sample (ECD and Δ*F* signal become more positive) (Figure S 52).

Following this procedure, the displacement of both (R_p)-MPCP and (S_p)-MPCP from the **CB8** cavity upon stepwise addition of 2 µM of the drug memantine was monitored with a 15 minutes of equilibration time between each addition. The variation in both the ECD and Δ*F* signal was monitored at 340 nm on the stepwise addition of memantine with the help of single point-time course ECD and Δ*F* measurements. This shows that ECD signal doesn't show any significant variation while the Δ*F* exhibits strong changes in the signal on the addition of memantine to both **CB8**•(R_p)-MPCP and **CB8**•(S_p)-MPCP (Figure S 51 (a, b) and Figure S 53 (a, b)). The Δ*F* signals were used instead of the FDCD signals in both the cases as we

are monitoring a fluorescence change on dye displacement (the measurements were done at same HT voltage).

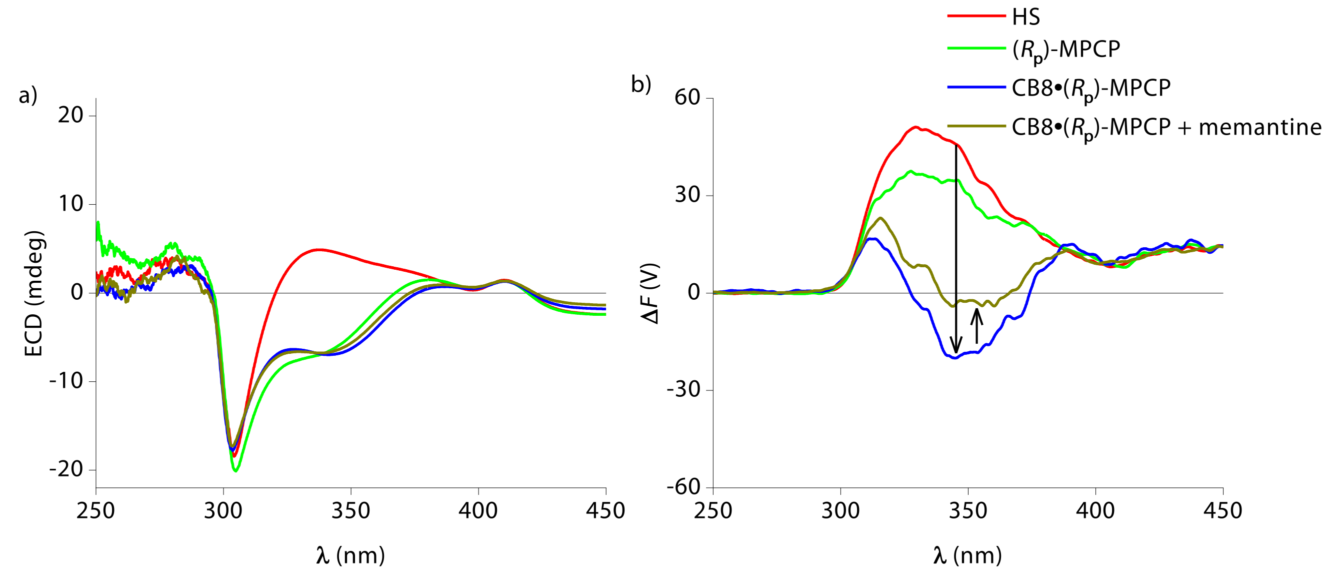

Figure S 50: (a) ECD and (b) Δ*F* spectra of **CB8•**(R_p)-MPCP (20 µM) in HS before and after addition of memantine (20 µM). The red line represents the ECD and Δ*F* background arising from HS alone and the green line represents the ECD and Δ*F* spectra of (R_p)-MPCP (20 µM) (dye alone) in HS. Parameters used: HT = 800 V, BW = 4 nm, Acc = 20, LP-Filter = 515 nm, T = 25°C.

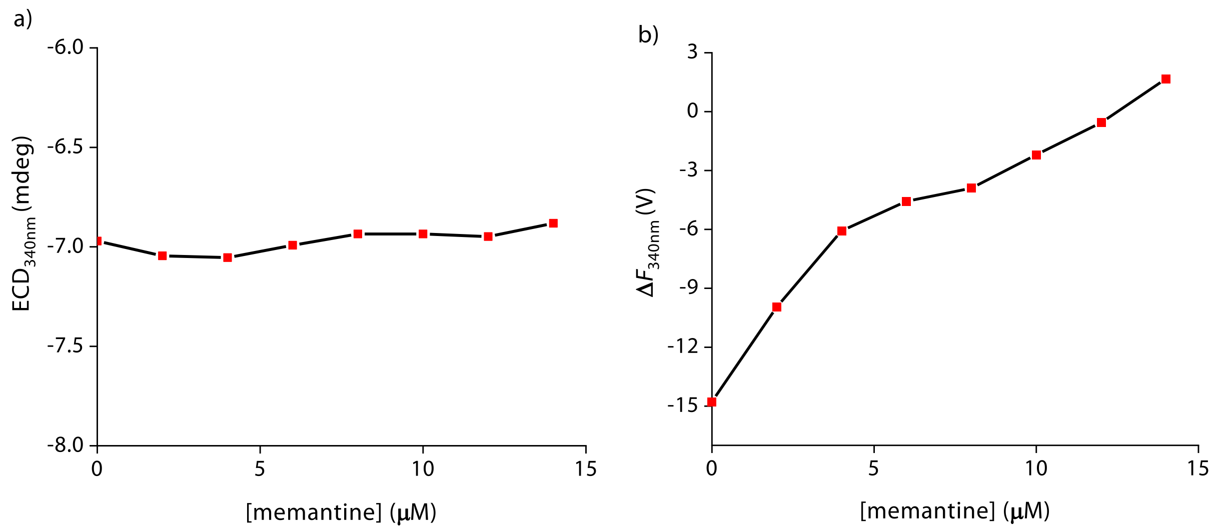

Figure S 51: Variation in the (a) ECD and (b) Δ*F* signal of the **CB8•**(R_p)-MPCP (20 µM) in HS when monitored at 340 nm on the stepwise addition 2 µM of the memantine (15 minutes equilibration time between each addition) by single point-time course measurements. Parameters used: HT = 800 V, BW = 4 nm, λ_{obs} = 340 nm, Data Pitch = 30 s, D.I.T = 30 s, $t_{measure}$ = 10 min, LP-Filter = 515 nm, T = 25°C.

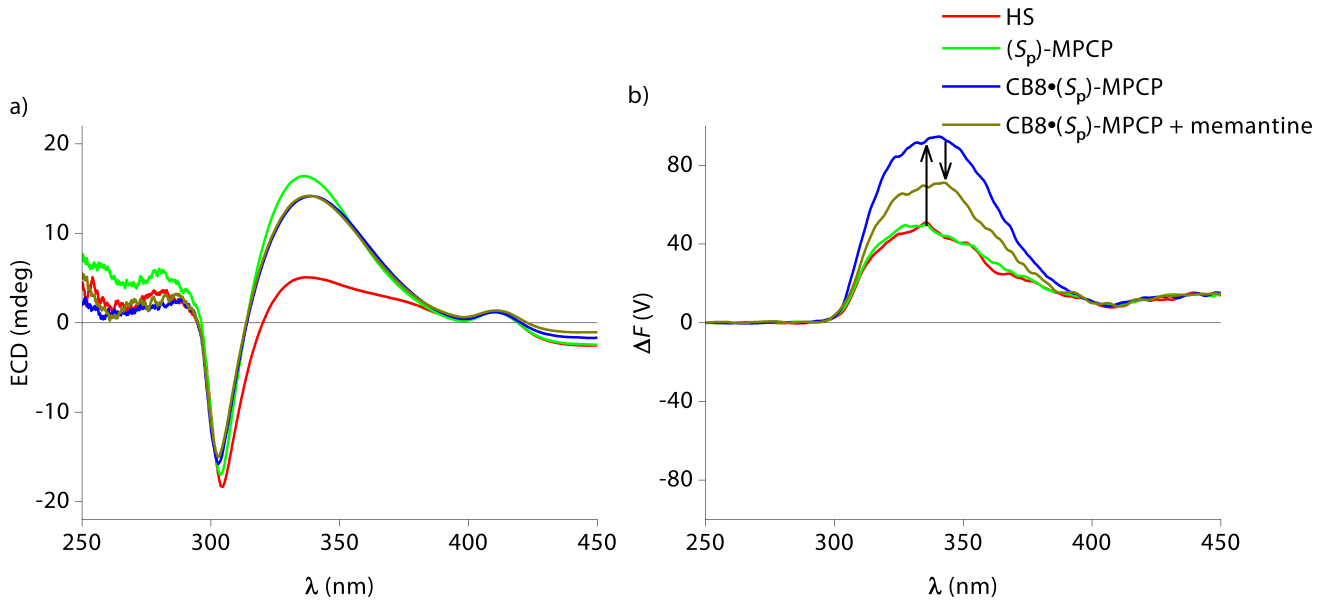

Figure S 52: (a) ECD and (b) Δ*F* spectra of **CB8•**(S_p)-MPCP (20 µM) in HS before and after addition of memantine (20 µM). The red line represents the ECD and Δ*F* background arising from HS alone and the green line represents the ECD and Δ*F* spectra of (S_p)-MPCP (20 µM) (dye alone) in HS. Parameters used: HT = 800 V, BW = 4 nm, Acc = 20, LP-Filter = 515 nm, T = 25°C.

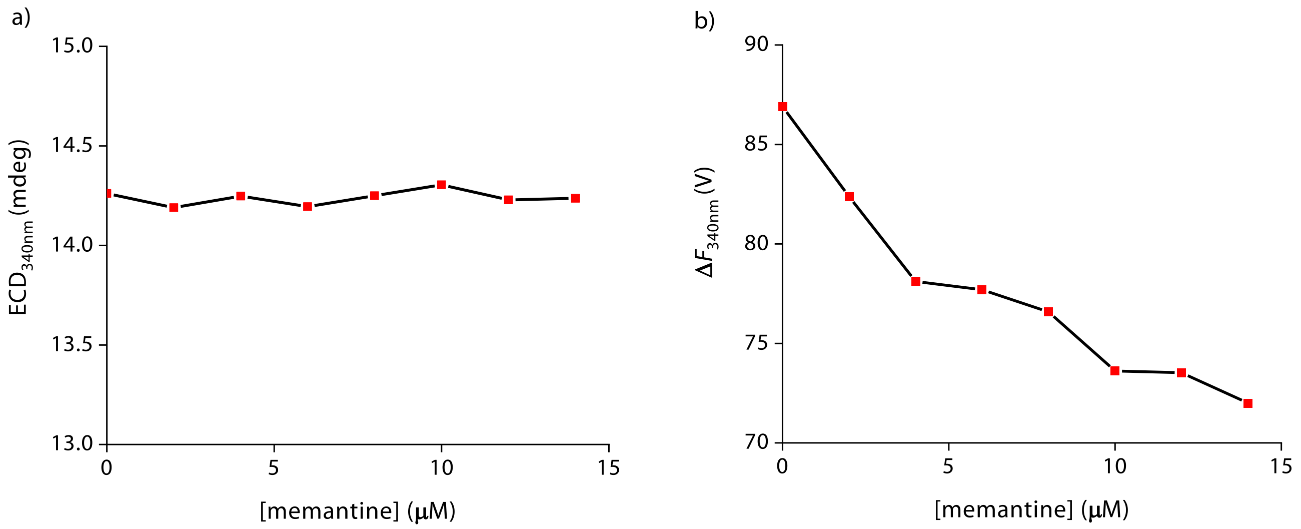

Figure S 53: Variation in the (a) ECD and (b) Δ*F* signal of the **CB8•**(S_p)-MPCP (20 µM) in HS when monitored at 340 nm on the stepwise addition 2 µM of the memantine (15 minutes equilibration time between each addition) by single point-time course measurements. Parameters used: HT = 800 V, BW = 4 nm, λ_{obs} = 340 nm, Data Pitch = 30 s, D.I.T = 30 s, $t_{measure}$ = 10 min, LP-Filter = 515 nm, T = 25°C.

Both **CB8**•(R_p)-MPCP and **CB8**•(S_p)-MPCP have the disadvantage for sensing in HS as the ECD and Δ*F* signals of the complex lies in the same region as the background Δ*F* and ECD signals arising from HS alone.

The red shifted absorption of the **CB8**•(S_p)-MVCP complex compared to the **CB8**•(S_p)-MPCP complex may be advantageous for sensing in blood serum (Figure S 24). Hence the **CB8**•(S_p)-MPCP was evaluated for the detection of Alzheimer's drug memantine in blood serum with the help of Δ*F* and ECD signals. The addition of 20 µM **CB8**•(S_p)-MVCP to the HS sample resulted in a change in both the ECD and Δ*F* spectra of HS (ECD and Δ*F* signal becomes more positive in the 380 nm to 440 nm region) (Figure S 54). These signals appear slightly shifted from the HS background ECD and Δ*F* signals. The stepwise addition of the drug memantine to the complex (15 minutes equilibration time between each addition) resulted in a decrease in both the ECD and Δ*F* signal of the complex when monitored at 407 nm (Figure S 55). Hence the displacement of the dye from **CB8** cavity by memantine could be followed by both ECD and Δ*F* with less interference from the HS background than was the case for MPCP as the indicator dye (Figure S 50 and Figure S 52).

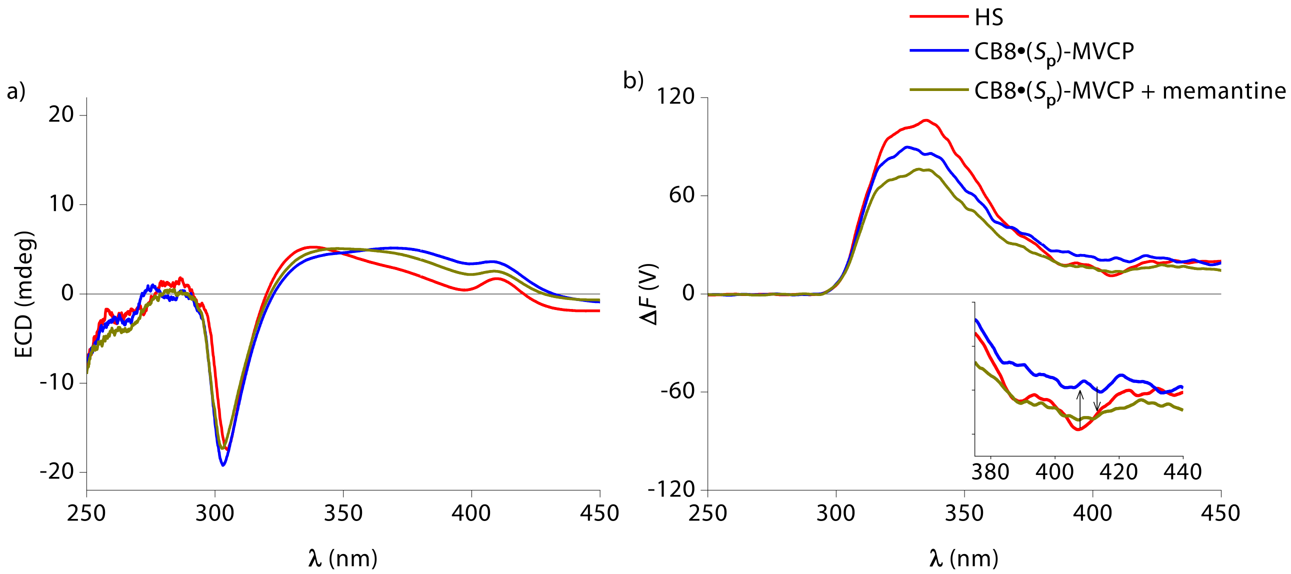

Figure S 54: (a) ECD and (b) Δ*F* spectra of **CB8•**(S_p)-MVCP (20 μM) in HS before and after addition of memantine (20 μM). The red line represents the ECD and Δ*F* background arising from HS alone. Parameters used: HT = 800 V, BW = 4 nm, Acc = 20, LP-Filter = 480 nm, T = 25°C.

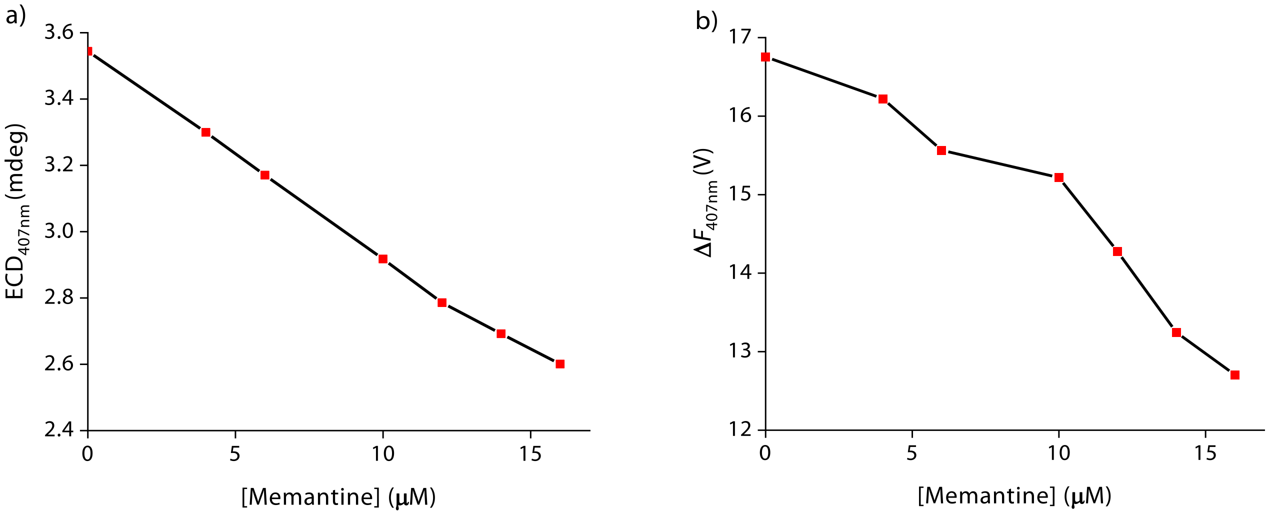

Figure S 55: Variation in the (a) ECD and (b) Δ*F* signal of the **CB8•**(S_p)-MVCP (20 μM) in HS when monitored at 407 nm on the stepwise addition 2 μM of memantine (15 minutes equilibration time between each addition) by single point-time course measurements. Parameters used: HT = 800 V, BW = 4 nm, λ_{obs} = 407 nm, Data Pitch = 30 s, D.I.T = 30 s, $t_{measure}$ = 10 min, LP-Filter = 480 nm, T = 25°C.

9. FDCD limitations and alternatives

9.1. Photoselection artefacts in FDCD

Care needs to be taken when performing FDCD measurements, especially when the system of interest exhibits a strongly polarized fluorescence, resulting in photoselection artefacts occurring from the anisotropic excitation of the emitted light.[35, 36] When the rotatory Brownian motion is restricted within the fluorescence lifetime of the chromophore used (especially for larger molecules and in viscous solvents), the electronic dipole transition moments of the absorption and emission bands may not be parallel or perpendicular to each other. Hence, in the presence of photoselection, the observed fluorescence will be polarized, that is, the fluorescence intensities of the vertical and horizontal components $F_{\parallel}$ and $F_{\perp}$ differ and the ratio $P_F = (F_{\parallel} - F_{\perp})/(F_{\parallel} + F_{\perp})$, known as fluorescence polarization, is non-vanishing. Thereby the observed differential emission upon excitation with a circularly polarized (CP) light source will not reflect the differential absorption of the CP light in a straightforward manner. From a practical viewpoint, however, polarization of emitted light can also arise from unwanted artefacts that occur in FDCD instrument. This can introduce additional artefacts in FDCD, due to the linear polarization present in the CP light produced by imperfect optical components in commercial ECD instruments. If the left-CP/right-CP excitation beams contain some residual linear polarization, a difference in the fluorescence signal will be detected not arising from the sample's optical activity.[15, 16, 37-39]

Photoselection artefacts can be eliminated by using an artefact-free FDCD unit (as shown before in Figure S 43) , which uses a unique design that includes a sandwiched elliptical cylinder mirror with two plane mirrors, so that all fluorescence light emitted in a circumferential direction from the cell is collected.[40, 41] The artefact-free FDCD unit not only eliminates artefacts due to fluorescence polarization, but also increases the signal-to-noise ratio (S/N) by collecting a large fraction of emitted radiation.[40, 41] The artefacts can also be reduced by using a polarizer at 85° in the emission path, but this decreases the S/N as fewer photons are detected.[15]

To check for the presence of photoselection artefacts in FDCD measurements, it is hence advisable to measure the fluorescence-detected linear dichroism (FDLD) spectrum which can be obtained simultaneously to FDCD and ECD (see Figure S 39 - Figure S 42). In the absence of signal artefacts, the FDLD spectrum should not give any signal. Furthermore, measurement of the excitation anisotropy of the system by using either the ECD or a fluorometer equipped with polarizers can give supplementary

information about the sample's properties and to check the influence of photoselection artefacts in FDCD measurements (see Figure S 40 (c)). Because photoselection is independent of the chirality, it can in general, be also identified by using enantiomeric pairs of the host or guest.

In order to check if the photoselection artefacts are completely eliminated when using the standard FDCD spectrometer at INT laboratory used in this study, the ECD and FDCD spectra of the achiral dye, rhodamine 6G and the achiral **CB8•MDPP** receptor was tested in both ethylene glycol (viscous medium) and water (Figure S 56 and Figure S 57). The achiral rhodamine 6G dye shows signal artefacts in both the Δ*F* and FDCD spectra collected when measured in both water and ethylene glycol. The photoselection artefacts are higher in ethylene glycol than water, because the former being more viscous.[42, 43] For the **CB8•MDPP** receptor, signal artefacts were present in both the Δ*F* and FDCD spectra when measured in ethylene glycol. However, the effects are lower when considering the FDCD values than the Δ*F* values. The artefacts are mainly present in the region where the chromophore absorbs. For **CB8•MDPP** in water, only minimal signal artefacts are present when considering the Δ*F* signal and are almost negligible in the FDCD data. The MDPP dye as well shows some signal artefacts in their Δ*F* spectrum when tested in both ethylene glycol and water (Figure S 57 (a, b)). However, the photoselection artefacts are higher for the complex **CB8•MDPP** when compared to the dye alone as the random motion of the dye molecule is restricted in the **CB8** cavity in case of the complex.

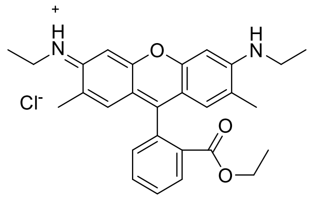

rhodamine 6G

Hence the Δ*F* spectra of the **CB8•MDPP** receptor in the presence of different chiral analytes tested in water needed to be corrected for the signal artefacts (see Section 9.1.1 for the artefact subtraction procedure).

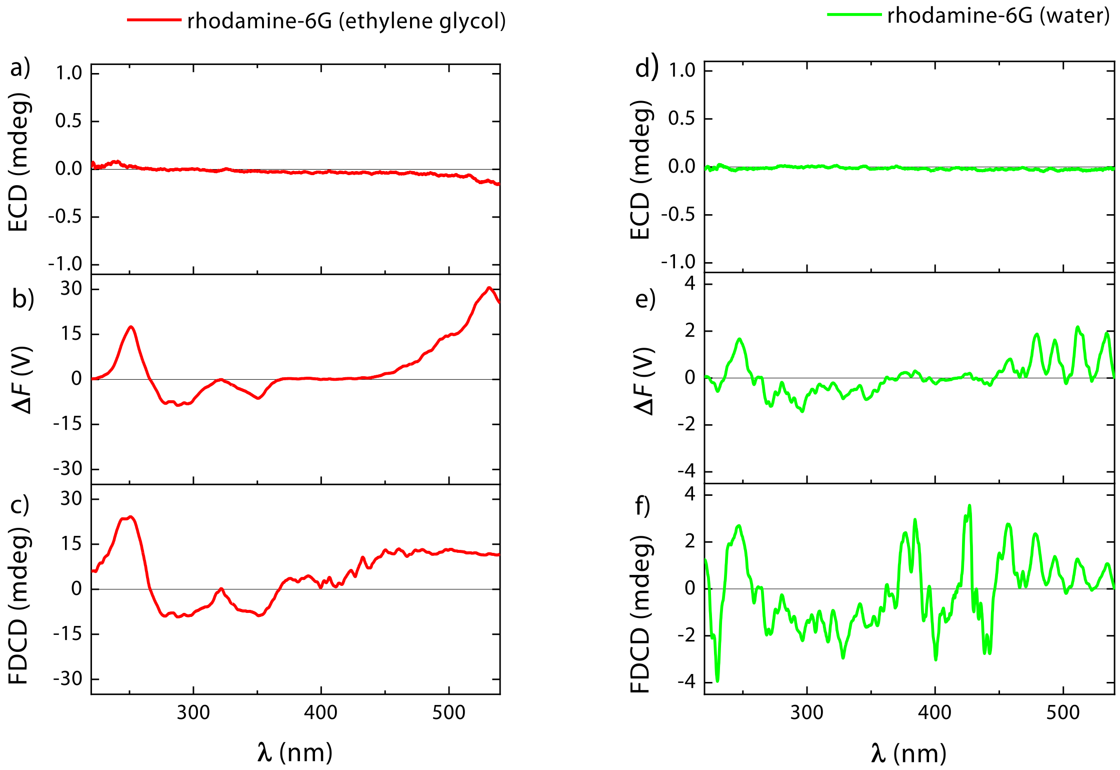

Figure S 56: (a) ECD, (b) Δ*F* and (c) FDCD spectra of rhodamine 6G (0.47 μM) in ethylene glycol. (d) ECD, (e) Δ*F* and (d) FDCD spectra of rhodamine 6G (0.47 μM) in DI-Water. Parameters used: HT = 700 V, BW = 4 nm, Acc = 20, LP-Filter = 540 nm.

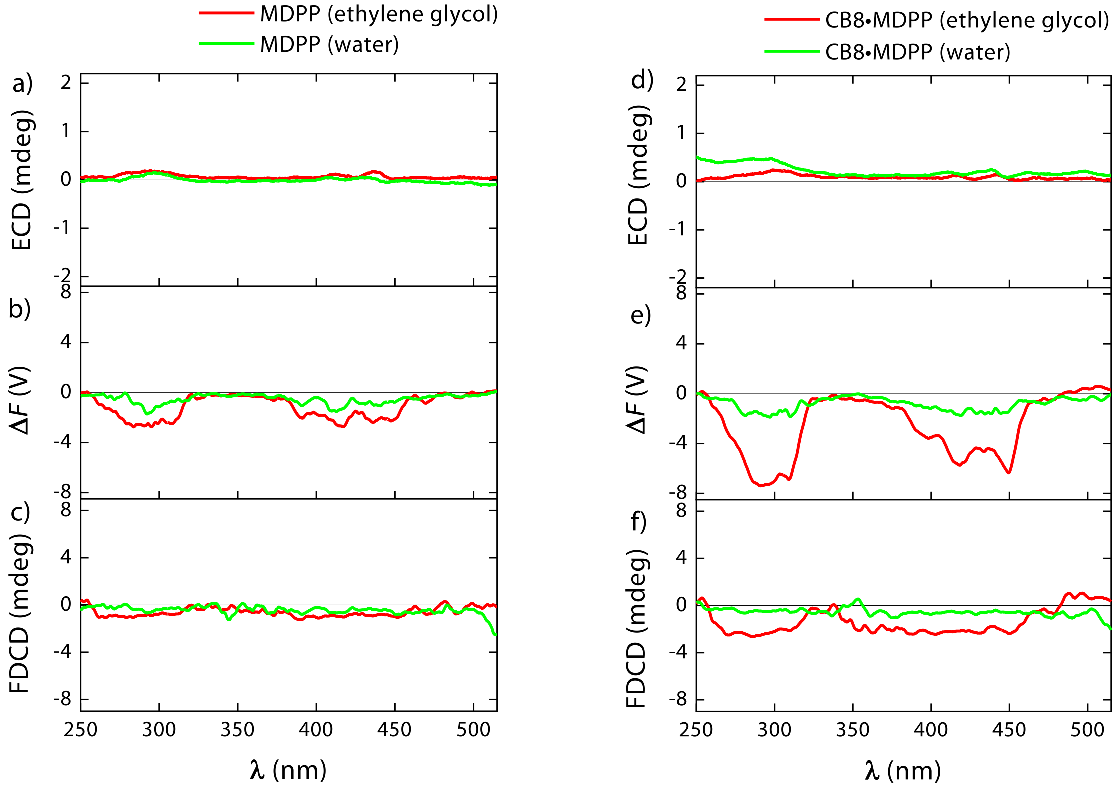

Figure S 57: (a) ECD, (b) Δ*F* and (c) FDCD spectra of MDPP (20 μM) in ethylene glycol and DI-Water. (c) ECD, (d) Δ*F* and (e) FDCD spectra of **CB8•MDPP** (20 μM) in ethylene glycol and DI-Water. Parameters used: HT = 520 V, BW = 4 nm, Acc = 20, LP-Filter = 515 nm

9.1.1. Correction of signal artefacts in FDCD

1- In most of the representative supramolecular host-guest systems, the photoselection artefacts, if any, present for the achiral receptor molecules, arise mainly near the excitation peak maxima of the fluorophore and are often clearly distinct from the induced chiroptical peaks that are observed in the presence of the chiral analyte. Besides, they are independent from the chirality of the guest or host. Hence the photoselection artefacts do not pose any obstacle for FDCD sensing applications.

2- However in cases where the signal artefacts arising from the achiral receptor molecule alone overlaps with the induced chiroptical peaks that are observed in the presence of the chiral analyte (for *e.g.*, in case of the Δ*F* signals from **CB8•MDPP** receptor), the signals were corrected by subtracting the signal artefacts arising from the receptor alone in all the cases. This is verified in case of the example below.

The racemisation reaction between D-Phe and L-Phe in the presence of **CB8•MDPP** receptor was studied using both ECD and Δ*F.* An addition of a 1:1 equivalent of D-Phe and L-Phe to the **CB8•MDPP** receptor results in a racemic mixture and hence should exhibit no ECD and Δ*F* signals. However, the racemic mixture still showed the signal artefacts in the Δ*F* spectrum similar to the spectrum of the receptor alone (Figure S 58 (a, b)). Hence the corrected Δ*F* spectrum is obtained by subtracting the signal artefacts arising from the receptor alone (Figure S 58 (c)). Overall, photoselection artefacts do not strongly interfere with the FDCD signals when accounted for properly.

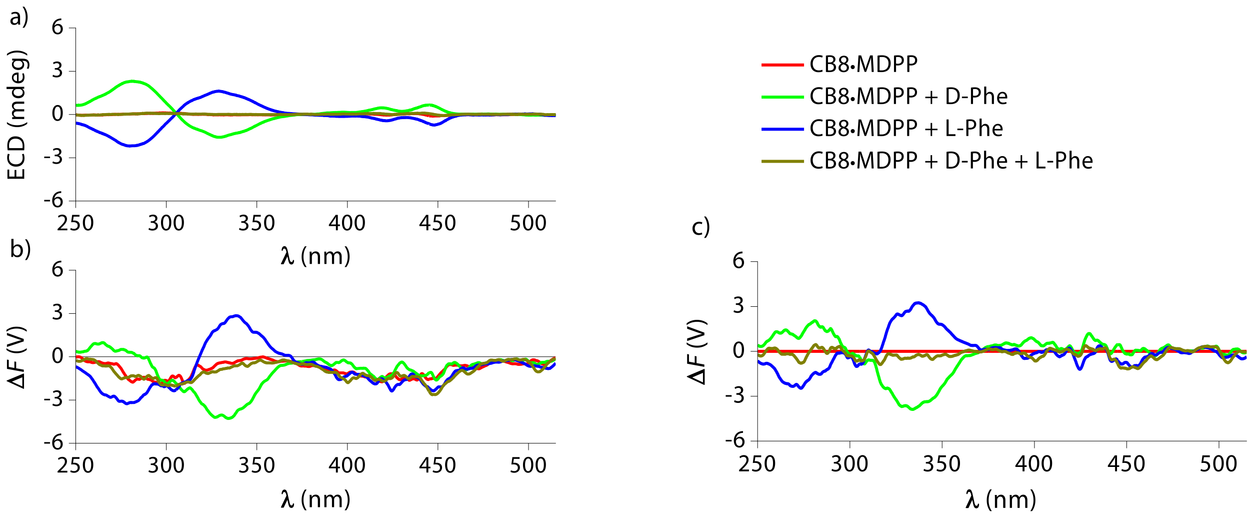

Figure S 58: (a) ECD and (b) Δ*F* spectra (not corrected for the signal artefacts arising from the receptor alone) of **CB8•MDPP** (20 µM) in the presence of D-Phe (50 µM), L-Phe (50 µM) and a 1:1 mixture of D-Phe and L-Phe (50 µM) in DI-Water. The red line represents the Δ*F* signals arising from the **CB8•MDPP** (20 µM) alone. (c) The corrected Δ*F* spectra obtained after subtracting the signal artefacts arising from the receptor alone. Parameters used: HT = 520 V, BW = 4 nm, Acc = 20, LP-Filter = 515 nm

9.2. Comparison of FDCD signal for spectra measured at different spectrometers

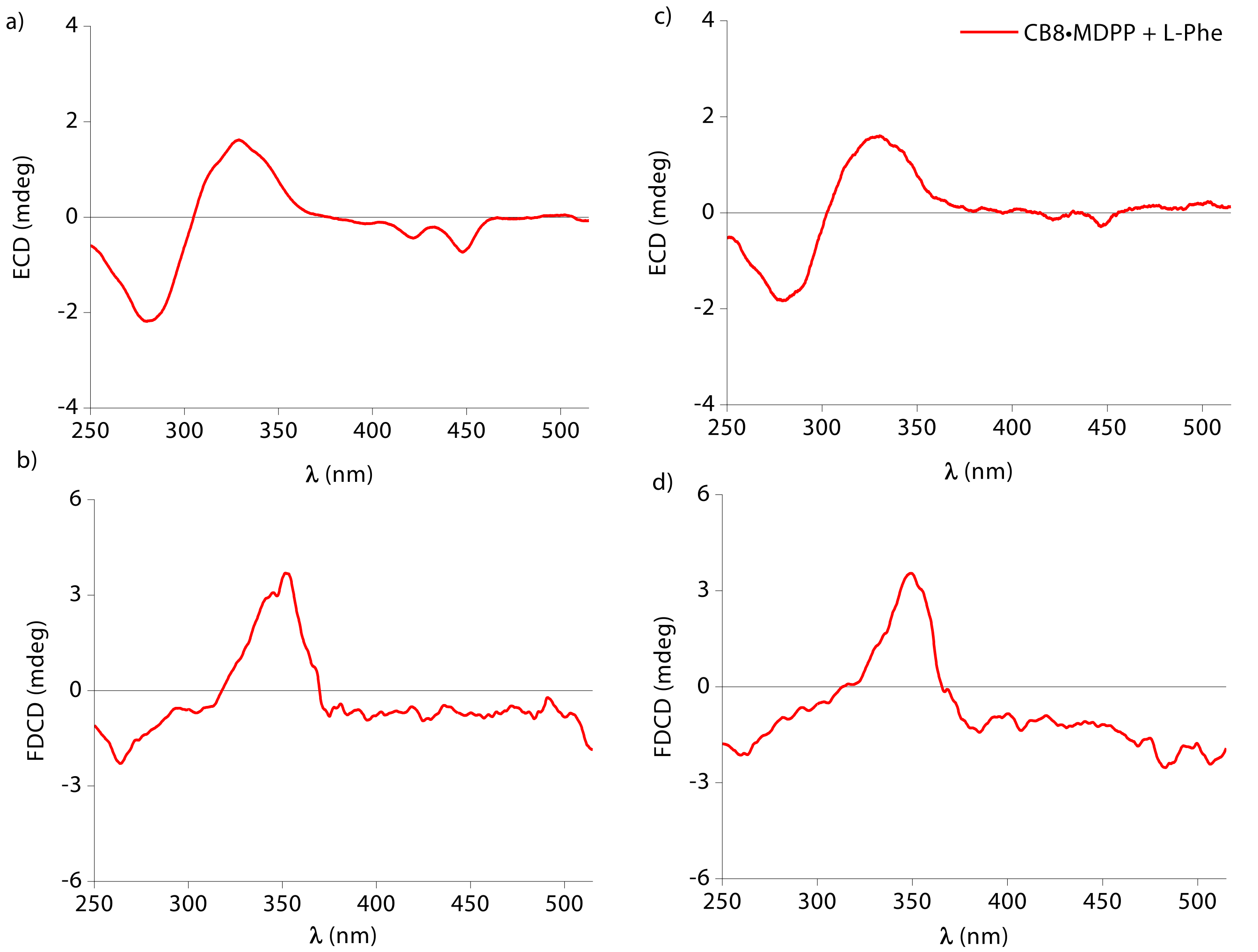

Figure S 59: (a) ECD and (b) FDCD spectra of **CB8•MDPP** (20 µM) in the presence of the L-Phe (50 µM) in DI-Water measured at the standard FDCD spectrometer at INT laboratory. Parameters used: HT = 520 V, BW = 4 nm, Acc = 20, LP-Filter = 515 nm, T = 25°C. (c) ECD and (d) FDCD spectra of **CB8•MDPP** (20 µM) in the presence of the L-Phe (50 µM) in DI-Water measured at the standard FDCD spectrometer at a JASCO facility in Pfungstadt. Parameters used: HT = 400 V, BW = 4 nm, Acc = 20, LP-Filter = 515 nm, T = 25°C.

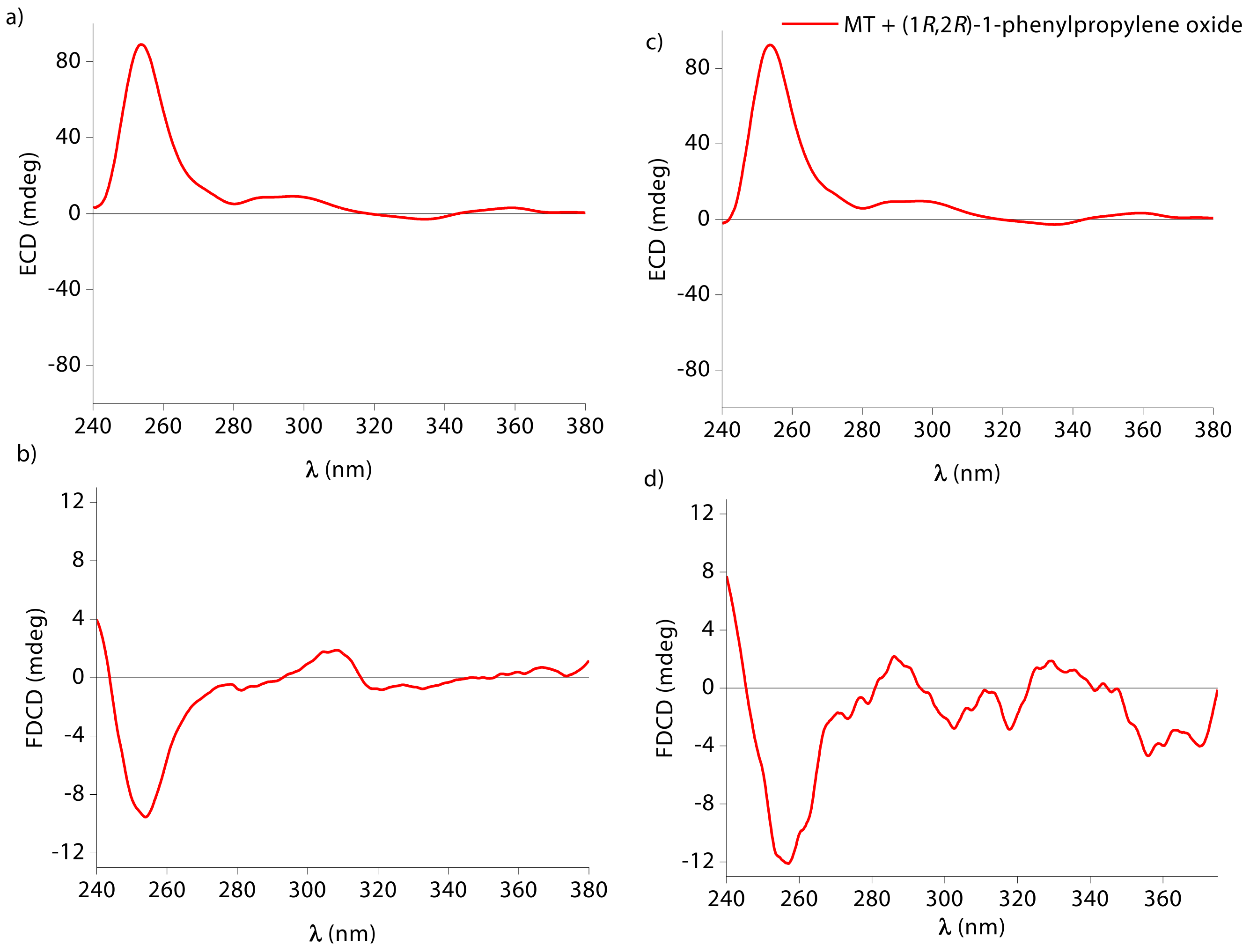

Figure S 60: (a) ECD and (b) FDCD spectra of freshly prepared **MT** receptor (100 µM) in the presence of (1*R*,2*R*)-1-phenylpropylene-oxide (500 µM) in DI-Water measured at the standard FDCD spectrometer at INT laboratory. Parameters used: HT = 630 V, BW = 4 nm, Acc = 20, LP-Filter = 380 nm, T = 25°C. (c) ECD and (d) FDCD spectra of **MT** receptor (100 µM) in the presence of (1*R*,2*R*)-1-phenylpropylene-oxide (500 µM) in DI-Water measured at the standard FDCD spectrometer at a JASCO facility in Pfungstadt. Parameters used: HT = 650 V, BW = 4 nm, Acc = 20, LP-Filter = 380 nm, T = 25°C.

10. References

1. J. Kim, I.-S. Jung, S.-Y. Kim, E. Lee, J.-K. Kang, S. Sakamoto, K. Yamaguchi and K. Kim, *J. Am. Chem. Soc.*, 2000, **122**, 540-541.
2. A. N. Basuray, H. P. Jacquot de Rouville, K. J. Hartlieb, T. Kikuchi, N. L. Strutt, C. J. Bruns, M. W. Ambrogio, A. J. Avestro, S. T. Schneebeli, A. C. Fahrenbach and J. F. Stoddart, *Angew. Chem. Int. Ed.*, 2012, **51**, 11872-11877.
3. C. Braun, E. Spuling, N. B. Heine, M. Cakici, M. Nieger and S. Bräse, *Adv. Synth. Catal.*, 2016, **358**, 1664-1670.
4. C. Braun, Ph.D, 2017.
5. M. Toda, Y. Inoue and T. Mori, *ACS Omega*, 2018, **3**, 22-29.
6. C. Rosini, R. Ruzziconi, S. Superchi, F. Fringuelli and O. Piermatti, *Tetrahedron: Asymmetry*, 1998, **9**, 55-62.
7. S. Sinn, E. Spuling, S. Brase and F. Biedermann, *Chem. Sci.*, 2019, **10**, 6584-6593.
8. C. Braun, S. Bräse and L. L. Schafer, *Eur. J. Org. Chem.*, 2017, **13**, 1760-1764.
9. A. Prabodh, D. Bauer, S. Kubik, P. Rebmann, F. G. Klärner, T. Schrader, L. Delarue Bizzini, M. Mayor and F. Biedermann, *Chem. Commun.*, 2020, **56**, 4652-4655.
10. F. Biedermann and W. M. Nau, *Angew. Chem. Int. Ed.*, 2014, **53**, 5694-5699.
11. PhotoChemCAD, (accessed 01-11-2019, DOI: https://omlc.org/spectra/PhotochemCAD).
12. C. N. Pace, F. Vajdos, L. Fee, G. Grimsley and T. Gray, *Protein Sci.*, 1995, **4**, 2411-2423.
13. D. H. Turner, I. Tinoco and M. Maestre, *J. Am. Chem. Soc.*, 1974, **96**, 4340-4342.
14. I. Tinoco and D. H. Turner, *J. Am. Chem. Soc.*, 1976, **98**, 6453-6456.
15. K. Tanaka, G. Pescitelli, K. Nakanishi and N. Berova, *Monatsh. Chem.*, 2005, **136**, 367-395.
16. E. W. Lobenstine, W. C. Schaefer and D. H. Turner, *J. Am. Chem. Soc.*, 1981, **103**, 4936-4940.
17. T. Nehira, C. A. Parish, S. Jockusch, N. J. Turro, K. Nakanishi and N. Berova, *J. Am. Chem. Soc.*, 1999, **121**, 8681-8691.
18. N. Berova, L. Di Bari and G. Pescitelli, *Chem. Soc. Rev.*, 2007, **36**, 914-931.
19. F. Biedermann, D. Hathazi and W. M. Nau, *Chem. Commun.*, 2015, **51**, 4977-4980.
20. L. Cao, M. Sekutor, P. Y. Zavalij, K. Mlinaric-Majerski, R. Glaser and L. Isaacs, *Angew. Chem. Int. Ed.*, 2014, **53**, 988-993.
21. Y. Yokoyama, H. Hikawa and Y. Murakami, *J. Chem. Soc., Perkin Trans. 1*, 2001, **12**, 1431-1434.
22. M. I. Childers, J. M. Longo, N. J. Van Zee, A. M. LaPointe and G. W. Coates, *Chem. Rev.*, 2014, **114**, 8129-8152.
23. E. N. Jacobsen, *Acc. Chem. Res.*, 2000, **33**, 421-431.
24. C. J. Thibodeaux, W. C. Chang and H. W. Liu, *Chem. Rev.*, 2012, **112**, 1681-1709.
25. R. E. Parker and N. S. Isaacs, *Chem. Rev.*, 1959, **59**, 737-799.
26. S. Bonollo, D. Lanari and L. Vaccaro, *Eur. J. Org. Chem.*, 2011, **2011**, 2587-2598.
27. J. Jiao, G. R. Douglas, J. D. Gingerich and L. M. Soper, *Mutat. Res-Fund. Mol. M.*, 1996, **372**, 141-145.
28. M. E. Dolle, H. J. Martus, M. Novak, N. J. van Orsouw and J. Vijg, *Mutagenesis*, 1999, **14**, 287-293.
29. Y. Sang, D. Yang, P. Duan and M. Liu, *Chem. Sci.*, 2019, **10**, 2718-2724.
30. L. L. Wang, Z. Chen, W. E. Liu, H. Ke, S. H. Wang and W. Jiang, *J. Am. Chem. Soc.*, 2017, **139**, 8436-8439.
31. S. Mondal, S. Ghosh and S. P. Moulik, *J. Photochem. Photobiol. B*, 2016, **158**, 212-218.
32. W. I. Goldburg, *Am. J. Phys.*, 1999, **67**, 1152–1160.
33. Polydispersity – What does it mean for DLS and chromatography?, (accessed 11-05-2020, DOI: https://www.materials-talks.com/blog/2017/10/23/polydispersity-what-does-it-mean-for-dls-and-chromatography/).
34. S. M. Patil, D. A. Keire and K. Chen, *The AAPS Journal*, 2017, **19**, 1760-1766.
35. B. Ehrenberg and I. Z. Steinberg, *J. Am. Chem. Soc.*, 1976, **98**, 1293-1295.
36. I. Tinoco, B. Ehrenberg and I. Z. Steinberg, *J. Chem. Phys.*, 1977, **66**, 916-920.
37. E. W. Lobenstine and D. H. Turner, *J. Am. Chem. Soc.*, 1979, **101**, 2205-2207.
38. E. W. Lobenstine and D. H. Turner, *J. Am. Chem. Soc.*, 1980, **102**, 7786-7787.
39. T. Nehira, *Monatsh. Chem.*, 2005, **136**, 477-487.
40. T. Nehira, K. Tanaka, T. Takakuwa, C. Ohshima, H. Masago, G. Pescitelli, A. Wada and N. Berova, *Appl. Spectrosc.*, 2005, **59**, 121-125.
41. Circular Dichroism Spectrometer J-1000 Series, (accessed 20-04-2020, DOI: https://www.jasco.de/uploads/files/J-1000-Series-Brochure.pdf).
42. J. R. Lakowicz, *Principles of Fluorescence Spectroscopy*, Springer, Boston, Massachusetts, 2006.
43. D. M. Jameson and J. A. Ross, *Chem. Rev.*, 2010, **110**, 2685-2708.
